# Undescribed Amaryllidaceae Alkaloids from *Zephyranthes citrina* and Their Cytotoxicity

**DOI:** 10.1021/acs.jnatprod.4c00825

**Published:** 2024-09-04

**Authors:** Jana Křoustková, Eliška Kohelová, Darina Muthná, Jiří Kuneš, Radim Havelek, Rudolf Vrabec, Milan Malaník, Daniela Suchánková, Jakub Chlebek, Jaroslav Jenčo, Štefan Kosturko, Lucie Cahlíková

**Affiliations:** ^a^Department of Pharmacognosy and Pharmaceutical Botany, ^c^Department of Bioorganic and Organic Chemistry, Faculty of Pharmacy, Charles University, Heyrovského 1203, 500 03 Hradec Králové, Czech Republic; b Department of Medical Biochemistry, Faculty of Medicine in Hradec Králové, Charles University, Šimkova 870, 500 03 Hradec Králové, Czech Republic; d Department of Natural Drugs, Faculty of Pharmacy, Masaryk University, Palackého třída 1946/1, 61200 Brno, Czech Republic

## Abstract

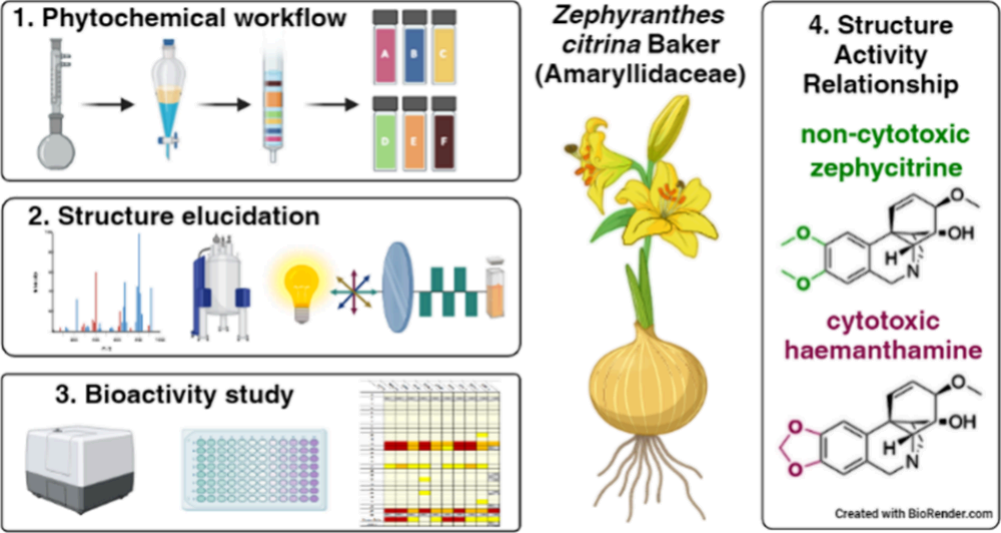

This phytochemical study presents the isolation of eight alkaloids from *Zephyranthes citrina* Baker. The structures of the new alkaloids, zephycitrine (**1**) and 6-oxonarcissidine (**2**), were established by analysis of spectroscopic and spectrometric data. Processing the EtOH extract under acid–base conditions yielded the unreported isolation artifacts **3** and **4**. This work also provides analytical data for alkaloids not properly described in the literature (**5** and **6**). The hippeastidine/zephyranine scaffolds in derivatives **3**, **4**, and **8**–**10** are also thoroughly discussed. Furthermore, a cytotoxicity screening of 25 Amaryllidaceae alkaloids isolated from *Z. citrina* was performed. Only the known alkaloids haemanthamine (**12**), haemanthidine (**13**), and lycorine (**27**) showed significant cell growth inhibition.

*Zephyranthes* Herb. is a genus of bulbous plants belonging to the Amaryllidaceae family, native to tropical and subtropical regions. Several species are cultivated due to their gorgeous flowers and are known by plant breeders as rain-lilies, owing to their tendency to flower after a rainy period. Species in the genus are the top 20 cultivated plants in the world and have been used as a folk medicine in many countries because of their high antioxidant, antiviral, antimicrobial, antimitotic, anticholinesterase, and cytotoxic activities.^1^

*Zephyranthes citrina* Baker was described in 1882 and is native to southeast Mexico and from Cuba to Haiti.^2^ The databases Plants of the World Online (POWO) and World Flora Online (WFO) also list several synonyms for *Z. citrina* (*Atamosco eggersiana* (Urb.) Britoon, *Z. sulphurea* Noter, and *Z. eggersiana* Urb.).^3^ The first phytochemical study on this genus was described by Boit and co-workers in 1957,^4^ and reported the isolation of the most cytotoxic of Amaryllidaceae alkaloids (AAs), lycorine and haemanthamine. Additional 25 alkaloids have been isolated and identified from this plant, sometimes called yellow rain lily.^3^

Cytotoxic activities of some AAs are already described in the literature.^5−7^ However, the cytotoxic activities of many AAs remain unknown. Alkaloids have historically played a pivotal role in drug discovery and development, providing novel drug scaffolds, particularly in the field of antitumor drugs.^8^ Recent findings highlight the potential of haemanthamine as an antitumor drug candidate. Mechanistically, this alkaloid functions as a protein synthesis inhibitor, interfering with the initial step of translation elongation.^5^ This action inhibits cell proliferation and leads either to the accumulation of Jurkat acute leukemic T-cells in the G1 and G2 phases of the cell cycle or induces cytotoxicity through apoptosis.^7^ In terms of structure–activity relationship investigations, there is a single, recently published report on the anticancer effect of haemanthamine derivatives using esterification in semisynthesis.^9^

## Results and Discussion

As a result of an ongoing phytochemical study on alkaloids of *Z. citrina*, eight additional alkaloids are herein reported, four of which are new (**1**–**4**). Known isolated compounds include eugenine (**5**), narcissidine (**6**), norlycoramine (**7**), and zephyranine C (**8**). All isolated compounds were identified by MS, NMR analyses, and specific rotation measurements. A complete description of NMR data is missing in the literature for alkaloids **5** and **6**. Therefore, we also discuss the data of **5** and **6**.
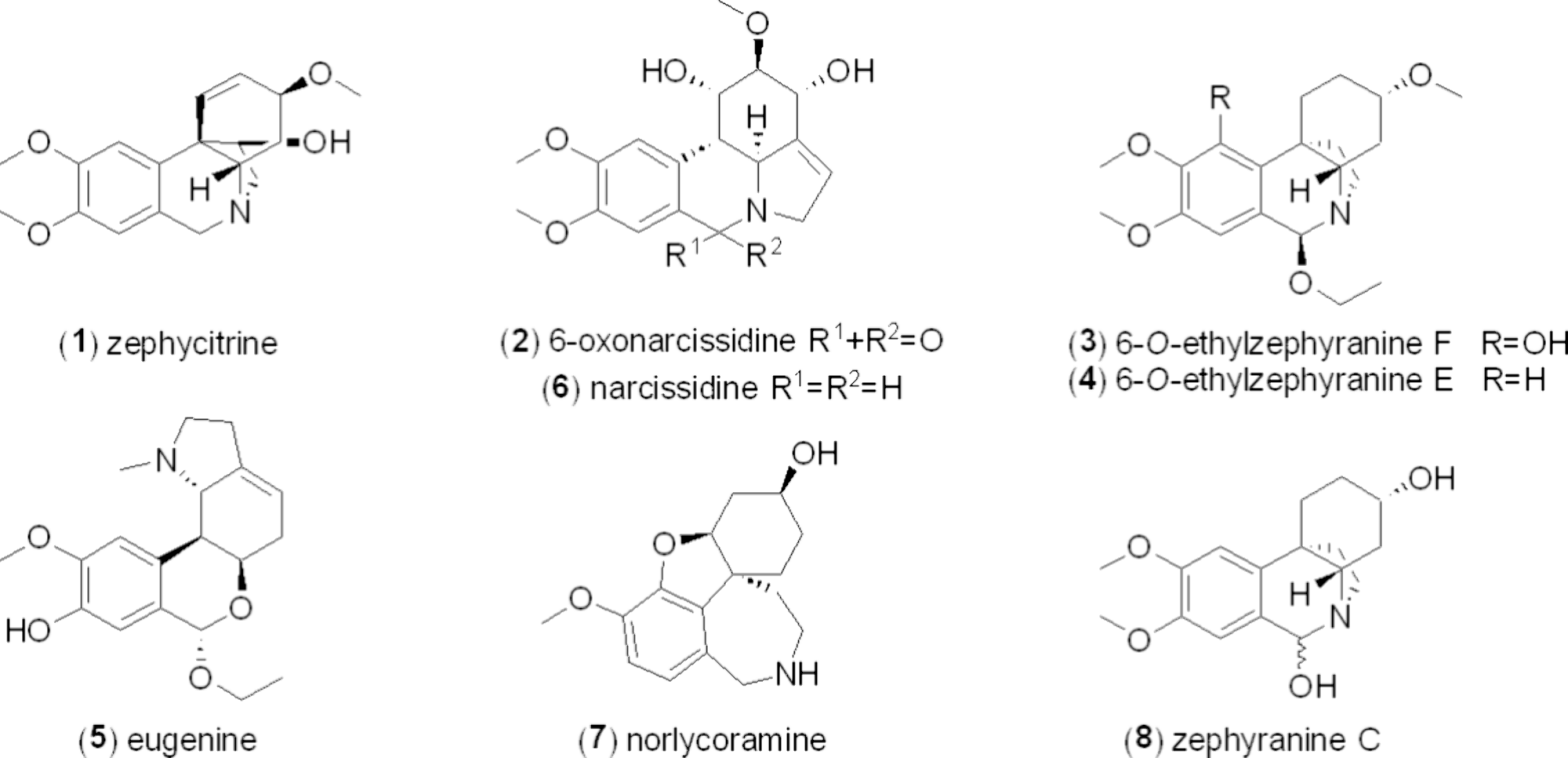


**Figure 1:**
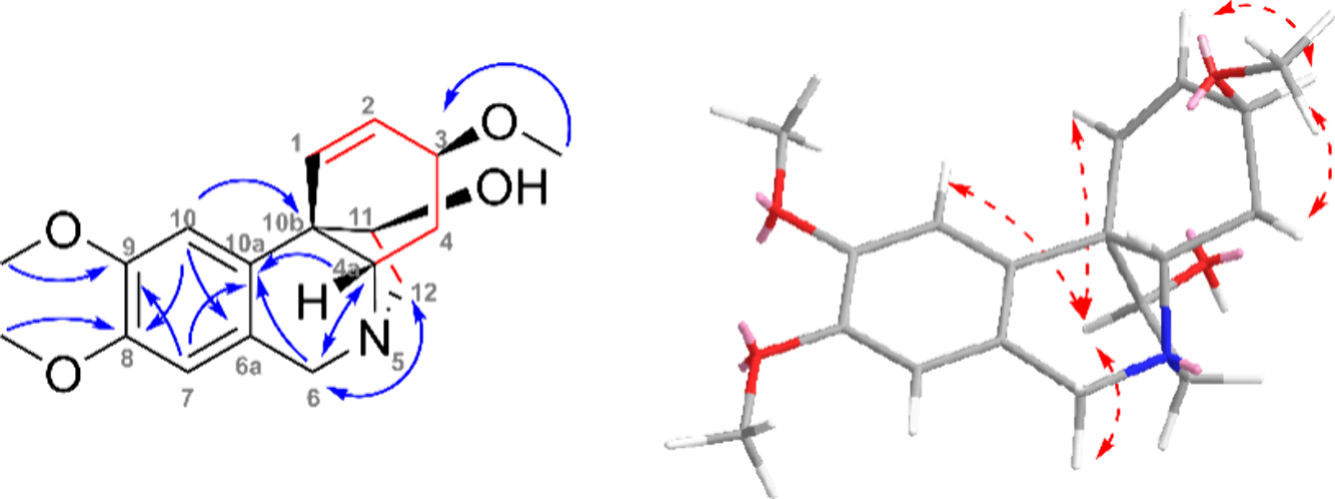
**Left:** Key HMBC (blue arrows), COSY and H2BC (red bonds) correlations of zephycitrine (**1**). **Right:** Key NOESY (red dashed arrows) correlations are shown for the energy-minimized structure of **1**.

The first compound was isolated as a yellowish powder. The HRESIMS of **1** showed a molecular ion peak [M + H]^+^ at *m*/*z* 318.1710 (calcd 318.1700), corresponding to the formula C_18_H_24_NO_4_^+^. The ^1^H NMR spectrum revealed 22 protons distributed in four signals in the aromatic region (δ_H_ 6.84, 6.52, 6.52, and 6.41), diastereomeric doublets of an N-benzyl group with a spin–spin coupling of 16.5 Hz (δ_H_ 4.42 and 3.79), two multiplets of deshielded OCH groups (δ_H_ 4.06–4.01 and 3.91–3.86), three deshielded OCH_3_ groups (δ_H_ 3.88, 3.83, and 3.38), two multiplets of N-deshielded protons (δ_H_ 3.48–3.41 and 3.40–3.35), and a multiplet for two protons in the aliphatic region (δ_H_ 2.22–2.03) (Table 1). Eight carbon resonances were found in the aromatic region of the ^13^C NMR spectrum and another ten in the aliphatic region. Analysis of HSQC, ^1^H, and ^13^C NMR data allowed the assignment of CH, CH_2_, and CH_3_ groups (see Table 1). By chemical shifts and cross-peaks found in the HMBC spectrum, two OCH_3_ groups (δ_C_ 55.9, 8-OCH_3_; and 56.1, 9-OCH_3_) were placed to a 1,2,4,5-tetrasubstituted aromatic ring. Cross-peaks of H-7/C-6 and H-6/C-7, respectively, located the N-benzylic position, and then from the cross-peaks H-6/C-10a and H-6/C-4a the whole tetrahydroisoquinoline moiety was revealed (see Figure 1). Correlations found in the COSY and H2BC (Heteronuclear 2-Bond Correlation) spectra supported the location of the CH and CH_2_ groups in the spin–spin system in cyclohexene connected to tetrahydroisoquinoline. The 5,10b-ethylene bridge was also identified using HMBC and COSY experiments. The relative configuration was established by NOESY experiment, e.g., the configuration of H-11 was confirmed by NOESY interaction with H-1 and H-10 (see Figure 1). This thorough analysis of the 2D NMR spectra introduced a molecule structurally close to the well-known haemanthamine, whose NMR data and optical rotation were compared with **1** establishing the absolute configuration 3*S*,4a*S*,10b*S*,11*R* for **1** (see Table S1). Another structurally related alkaloid was found, narcidine, for which only low-resolution MS and ^1^H NMR data have been published, together with specific rotation.^10^ Therefore, compound **1** was named zephycitrine.

**Table 1:** ^1^H NMR (500 MHz) and ^13^C NMR (125.7 MHz) Data of Compounds **1**–**5**

	**1**^a^		**2**^a^		**3**^a^		**4**^a^		**5**^b^	
no.	δ_C_, type	δ_H_, mult. (*J* in Hz)	δ_C_, type	δ_H_, mult. (*J* in Hz)	δ_C_, type	δ_H_, mult. (*J* in Hz)	δ_C_, type	δ_H_, mult. (*J* in Hz)	δ_C_, type	δ_H_, mult. (*J* in Hz)
1	126.9, CH	6.52, d (10.0)^c^	67.6, CH	4.78, bs	26.4, CH_2_	3.22–3.09, m	26.2, CH_2_	2.50–2.44, m		
						1.80–1.68, m		1.73, td (13.6, 4.3)		
2	132.4, CH	6.41, dd (10.0, 4.9)	80.6, CH	3.88, t (2.1)	27.7, CH_2_	2.07–1.97, m	27.4, CH_2_	2.17–2.08, m	57.7, CH_2_	3.21, ddd (10.3, 7.7, 2.8)
						1.50–1.39, m		1.55–1.47, m		2.42–2.32, m
3	72.7, CH	3.91–3.86, m^c^	67.9, CH	4.72, bs	77.7, CH	3.22–3.09, m	77.9, CH	3.18, tt (10.9, 3.9)	28.7, CH_2_	2.58–2.48, m
3a			138.5, C						140.4, C	
3a′			59.4, CH	4.85, d (13.3)						
4	28.1, CH_2_	2.22–2.03, m	122.9, CH	5.95–5.92, m	33.4, CH_2_	2.07–1.97, m	32.9, CH_2_	2.17–2.08, m	117.9, CH	5.55–5.50, m
						1.25–1.17, m		1.32–1.26, m		
4a	62.9, CH	3.48–3.41, m			61.1, CH	3.22–3.09, m	60.1, CH	3.30–3.23, m		
5			52.5, CH_2_	4.67, ddd (16.2, 4.4, 2.1)					32.6, CH_2_	2.69–2.60, m
				4.41–4.36, m						2.32–2.25, m
5a									68.0, CH	4.28, dd (5.7, 1.8)
6	60.9, CH_2_	4.42, d (16.5)			95.0, CH	4.53, s	94.9, CH	4.72, s		
		3.79, d (16.5)								
6a	125.2, C				129.9, C		125.2, C			
7	109.7, CH	6.52, s^c^	162.5, C		103.9, CH	6.38, s	111.5, CH	6.74, s	98.4, CH	5.57, s
7a			124.9, C						127.7, C	
8	148.0, C		111.6, CH	7.61, s	150.4, C		147.6, C		115.5, CH	6.75, s
9	147.9, C		148.0, C		135.2, C		148.9, C		147.1, C	
10	106.3, CH	6.84, s	151.9, C		146.3, C		105.5, CH	6.67, s	148.7, C	
10a	133.8, C				126.4, C		140.9, C			
10b	49.9, C				43.1, C		42.0, C			
11	80.0, CH	4.06–4.01, m	105.6, CH	6.81, s	33.1, CH_2_	2.23–2.14, m	34.6, CH_2_	2.27–2.18, m	113.6, CH	6.95, s
						1.80–1.68, m		1.63–1.55, m		
11a			131.3, C						130.0, C	
11b			43.2, CH	3.20, dd (13.3, 1.2)					44.4, CH	2.45, dd (9.7, 1.8)
11c									69.1, CH	2.86, d (9.7)
12	63.3, CH_2_	3.48–3.41, m			47.4, CH_2_	3.35–3.25, m	47.1, CH_2_	3.47–3.42, m		
		3.40–3.35, m				2.64–2.56, m		2.76–2.66, m		
2-OCH_3_			58.4, CH_3_	3.49, s						
3-OCH_3_	56.7, CH_3_	3.38, s^c^			55.7, CH_3_	3.39, s	55.85, CH_3_	3.40, s		
6-OCH_2_CH_3_					63.7, CH_2_	4.06, dq (9.5, 7.1)	63.7, CH_2_	4.19–4.20, m		
						3.72–3.64, m		3.80–3.72, m		
6-OCH_2_CH_3_					15.6, CH_3_	1.30, t (7.1)	15.6, CH_3_	1.30 t (7.0)		
7-OCH_2_CH_3_									64.6, CH_2_	3.93–3.83, m^c^
										3.70, dq (9.7, 7.1)
7-OCH_2_CH_3_									15.7, CH_3_	1.26, t (7.1)
8-OCH_3_	55.9, CH_3_	3.83, s			55.7, CH_3_	3.85, s	55.79, CH_3_	3.87, s		
9-OCH_3_	56.1, CH_3_	3.88, s^c^	56.2, CH_3_	3.95, s	60.9, CH_3_	3.86, s	56.0, CH_3_	3.86, s		
10-OH						6.05, bs				
10-OCH_3_			56.2, CH_3_	3.97, s					56.5, CH_3_	3.88, s^c^
NCH_3_									44.1, CH_3_	2.14, s

a In CDCl_3_.

b In CD_3_OD.

c Overlapped.

**Figure 2:**
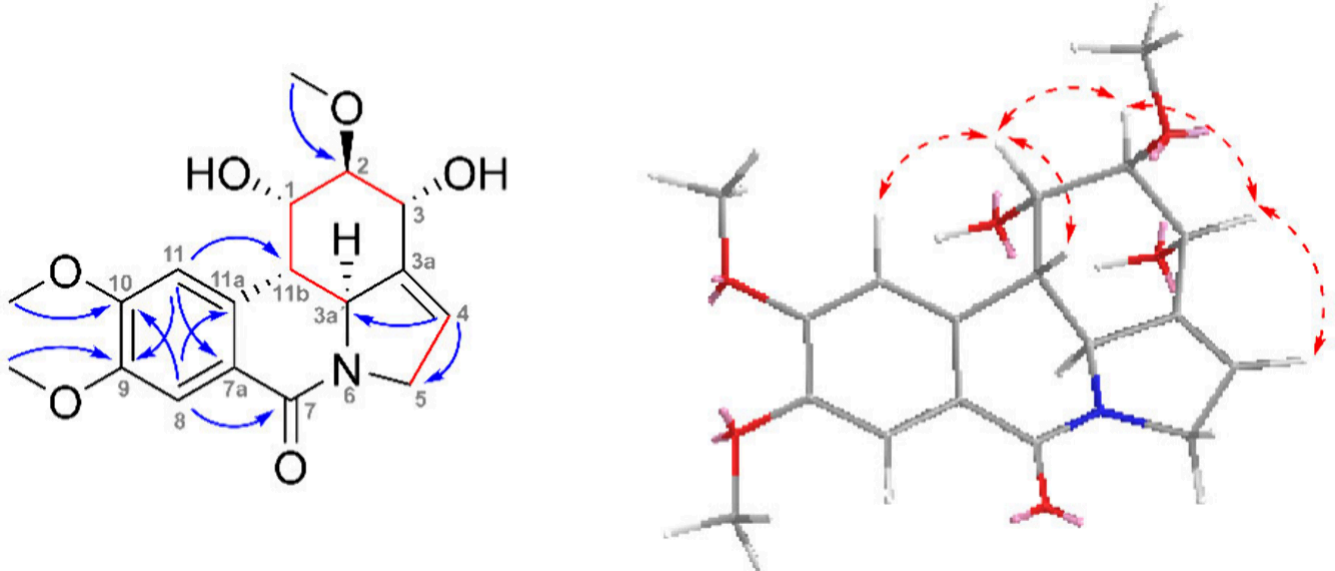
**Left**: Key HMBC (blue arrows), COSY, and H2BC (red bonds) correlations of 6-oxonarcissidine (**2**). **Right**: Key NOESY (red dashed arrows) correlations are shown for the energy-minimized structure of **2**.

Alkaloid **2** was isolated as white crystals. The HRESIMS showed a protonated molecule [M + H]^+^ at *m*/*z* 348.1446 (calcd 348.1441) corresponding to a molecular formula C_18_H_21_NO_6_, when taken together with ^1^H and ^13^C NMR data. The ^1^H NMR spectrum showed three signals in an aromatic region (δ_H_ 7.61, s; 6.81, s; and 5.95–5.92, m) and ten signals in a deshielded aliphatic region, where three singlets corresponded to OCH_3_ groups (δ_H_ 3.97, 3.95, and 3.49) (Table 1). The ^13^C NMR spectrum revealed the presence of nine sp^2^-carbons and nine sp^3^-carbons. The cross-peaks found in the HSQC spectrum assigned the protons to their respective carbons, presenting the CH/CH_2_/CH_3_ groups (see Table 1). Employing the HMBC experiment, H-8 (δ_H_ 7.61, s) and H-11 (δ_H_ 6.81, s) were assigned to the 1,2,4,5-tetrasubstituted benzene ring. H-8 showed interactions with C-7 (δ_C_ 162.5), C-10 (δ_C_ 151.9), and C-11a (δ_C_ 131.3), whereas H-11 showed cross-peaks with C-7a (δ_C_ 124.9), C-9 (δ_C_ 148.0), and C-11b (δ_C_ 43.2). In the COSY experiment, spin–spin systems were recognized, establishing connections for H-3a′/H-11b/H-1/H-2/H-3 and H-4/H-5. Then in the HMBC spectrum, a cross-peak of H-4 (δ_H_ 5.95–5.92) with C-3a′ (δ_C_ 59.4) revealed the location of the isolated double bond in positions 3a and 4 (see Figure 2). The configuration was established from the NOESY experiment, *J*-couplings, and a comparison with the chemical shifts of structurally related compounds – narcissidine (**6**) and 1-*O*-acetyl-3-*O*-methyl-6-oxonarcissidine,^11^ which is shown in Table S2. In the NOESY spectrum, cross-peaks were observed between H-11b/H-1, H-1/H-2, and H-2/H-3, but there was no interaction between H3a′ and H-11b. Together with spin–spin splitting patterns, a *trans*-diaxial position was established for H-3a′ and H-11b (*J* = 13.3 Hz), a *trans*-diequatorial arrangement for H-1, H-2, and H-3, and an axial–equatorial orientation for H-1 and H-11b (Figure 2). Also, a comparison with structurally related compounds (Table S2) shows that compound **2** has a negative optical rotation value, which follows the trend of these compounds. Thus, the absolute configuration was set as 1*S*,2*R*,3*R*,3a′*S*,11*S* for 6-oxonarcissidine (**2**).

**Figure 3:**
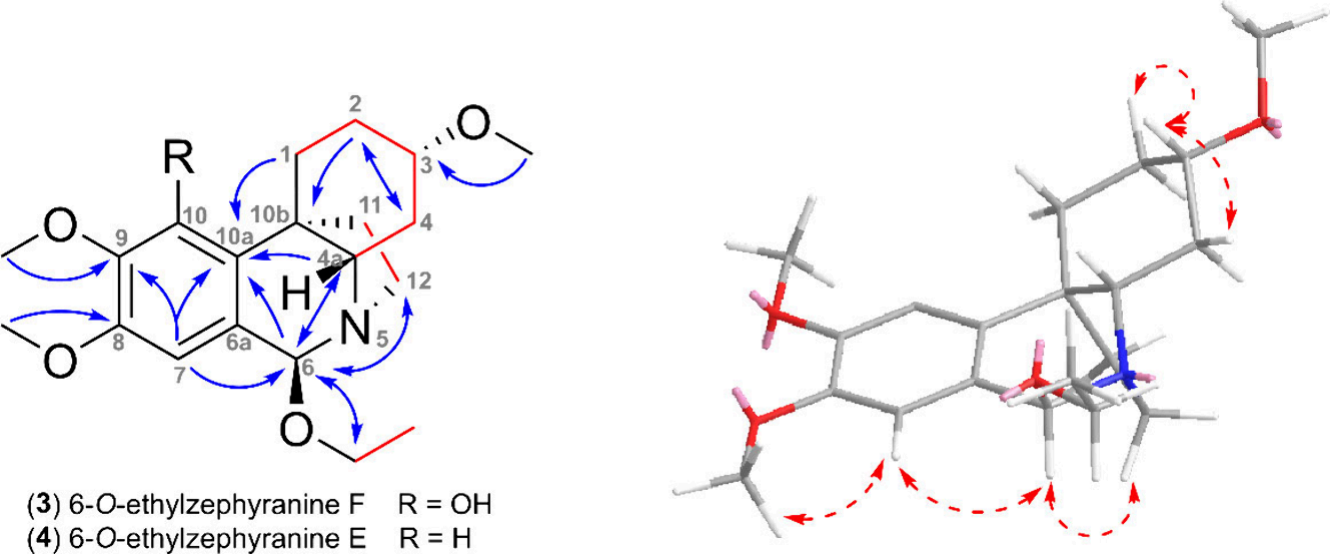
**Left**: Key HMBC (blue arrows), COSY, and H2BC (red bonds) correlations of compounds **3** and **4**. **Right:** Key NOESY (red dashed arrows) correlations are shown for the energy-minimized structures of compound **3** and **4**.

Compound **3** was obtained as a pale white amorphous solid. A protonated molecule [M + H]^+^ at *m*/*z* 364.2120 (calcd 364.2119) was found in the HRESIMS. The ^1^H NMR spectrum showed 29 protons, of which only two signals, one sharp singlet (δ_H_ 6.38) and one broad singlet (δ_H_ 6.05, Ar-OH), were in the aromatic region, then three deshielded singlets of OCH_3_ groups (δ_H_ 3.86, 3.85, and 3.39), together with other deshielded signals, multiplets in the aliphatic region closed by the presence of a triplet corresponding to three protons (δ_H_ 1.30, t, *J* = 7.1 Hz) (Table 1). The ^13^C NMR spectrum showed only six carbons in the aromatic region, one strongly deshielded sp^3^ carbon (δ_H_ 95.0), and twelve carbons in the aliphatic region. Analysis of HSQC, ^1^H, and ^13^C NMR data allowed the assignment of CH, CH_2_, and CH_3_ groups (see Table 1). From chemical shifts and cross-peaks found in the HMBC spectrum, two OCH_3_ groups (δ_C_ 55.7, 8-OCH_3_; and 60.9, 9-OCH_3_) were placed to the 1,2,3,4,5-pentasubstituted aromatic ring, of which H-7 (δ_H_ 6.38) correlated with carbon C-6 (δ_C_ 95.0). H-6 (δ_H_ 4.53, s) had cross-peaks with C-4a (δ_C_ 61.1), C-10a (δ_C_ 126.4), C-12 (δ_C_ 47.4), and a carbon of the O**C**H_2_CH_3_ group (δ_C_ 63.7). Correlations found in the COSY and H2BC spectra supported the location of the CH and CH_2_ groups in the spin–spin system of the cyclohexane moiety of this octahydrophenanthridine (Figure 3). The only quaternary sp^3^-carbon (δ_C_ 43.1, C-10b) was the binding site of the ethylene bridge from the tertiary nitrogen. The second isolation artifact **4** was obtained as a pale white amorphous solid. The HRESIMS showed a protonated molecule [M + H]^+^ at *m*/*z* 348.2177 (calcd 348.2170), corresponding to a molecular formula C_20_H_29_NO_4_, when taken together with ^1^H and ^13^C NMR data. The 1D NMR data were similar to that of **3**, except for the aromatic region. HMBC cross-peaks of H-7 (δ_H_ 6.74, s) with C-6 (δ_C_ 94.9), C-9 (δ_C_ 148.9), and C-10a (δ_C_ 140.9), together with cross-peaks of H-10 (δ_H_ 6.67, s) with C-6a (δ_C_ 125.2), C-8 (δ_C_ 147.6), and C-10b (δ_C_ 42.0) determined the presence of a 1,2,3,4-tetrasubstituted benzene ring instead of a pentasubstituted one, as in **3** (Figure 3).

The relative configuration of compounds **3** and **4** was established from data comparison and correlations found in the NOESY experiment and *J*-couplings (Table 1, Figure 3). The cross-peak of H-6 with H-12 supported the orientation on the same side. The absence of NOESY interaction between H-4a and H-6 suggested their opposite orientation on the cycle. Although the NOESY experiment was not applicable for H-3 (too close to the H-4a signal), its configuration was recognized from *J*-couplings. The splitting pattern of this triplet of triplets (*J* = 10.9 and 3.9 Hz) suggested its axial position on the cycle. Therefore, a relative configuration of 3*S*,4a*S*,6*S*,10b*S* was established for **3** and **4** (the absolute configuration is discussed below).

These two compounds were recognized as isolation artifacts due to the ethyl substitution in the hemiaminal ether group. Although similar alkaloids with a 6-hydroxy substitution are usually reported as a diastereomeric mixture of 6-epimers, e.g., haemanthidine,^12^ zephyranines C-F,^13^ and 6-hydroxyhippeastidine,^14^ compounds **3** and **4** were obtained as only one diastereomer. The ECD analysis of these two compounds is later discussed, along with the parent molecules, elucidating the absolute configuration. Thus, compound **3** was named 6-*O*-ethylzephyranine F and compound **4** 6-*O*-ethylzephyranine E.

**Figure 4:**
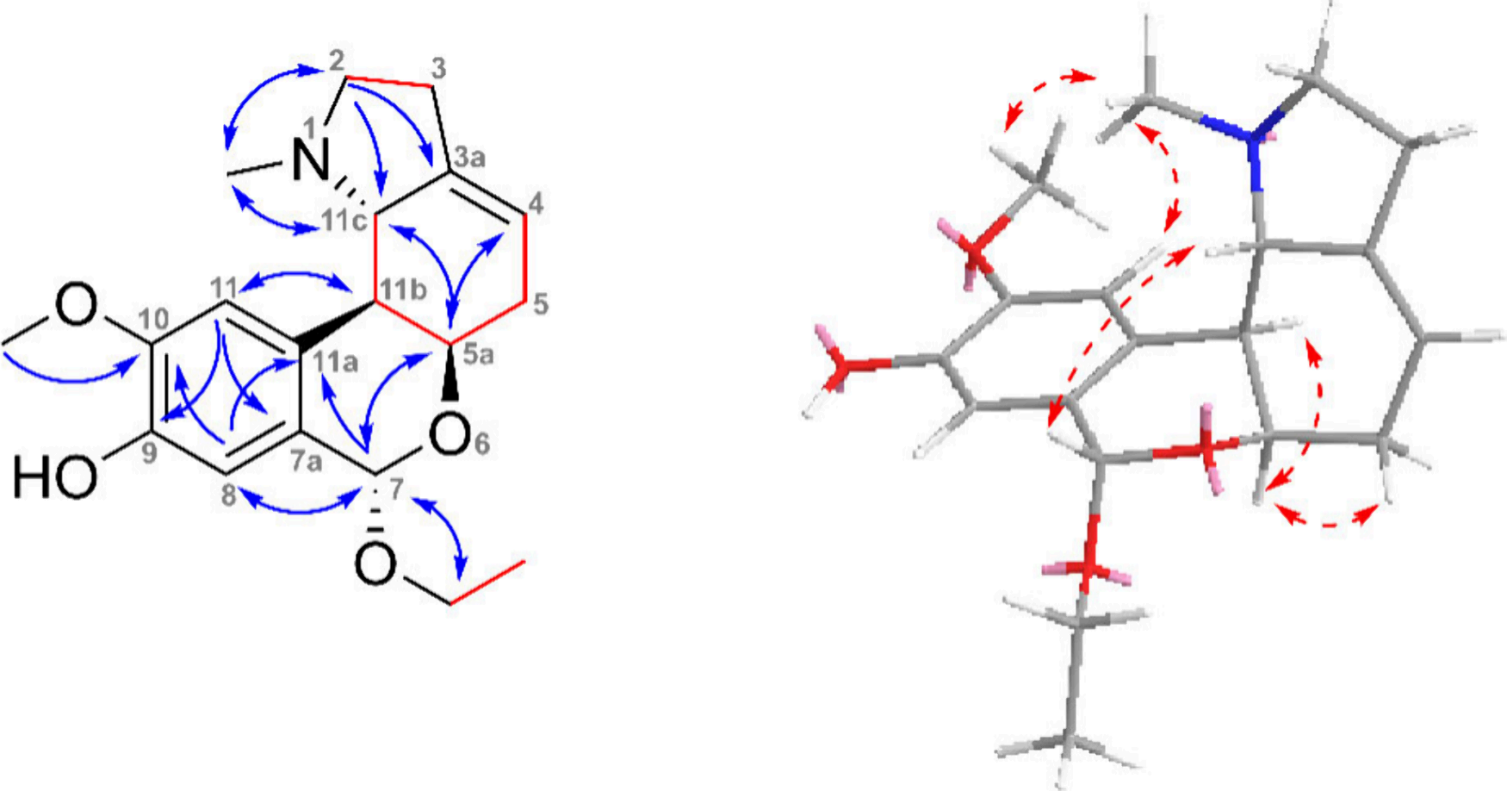
**Left:** Key HMBC (blue arrows), COSY, and H2BC (red bonds) correlations of compound **5**. **Right:** Key NOESY (red dashed arrows) correlations are shown for the energy-minimized structure.

The third isolation artifact identified in this work was eugenine (**5**), previously reported in an extract of *Narcissus eugeniae* (currently recognized as *Narcissus confusus* Pugsley in WFO).^15^ Although this alkaloid was described in the literature, the only data reported are from X-ray crystallography (both enantiomers in an orthorhombic crystal).^16^ This article was probably supposed to link the data sets with the article “Bastida et al. (1989) *J. Nat. Prod.* In press”. But, with our best efforts, we have not been able to track down such a paper, and since our work also focuses on the availability of structural analysis data for future phytochemical works, we report a complete set of HRMS, NMR, and polarimetry data for **5**. The compound was isolated as a yellowish powder. The HRESIMS of **5** showed a molecular ion peak [M + H]^+^ at *m*/*z* 332.1868 (calcd 332.1856), corresponding to the formula C_19_H_26_NO_4_^+^. Table 1 shows ^1^H and ^13^C NMR chemical shifts unambiguously assigned in CH/CH_2_/CH_3_ groups according to interactions found in the HSQC spectrum. The positions of each group were proven in COSY, H2BC, and HMBC experiments. The relative configuration was elucidated by the NOESY experiment, together with the evaluation of *J*-couplings (Figure 4, Table 1). Since Via and co-workers pointed out the presence of a mixture of enantiomers, this molecule was verified using Pirkel’s reagent by an addition of four equivalents of (*R*)-1-(9-anthryl)-2,2,2-trifluoroethanol into the CDCl_3_ solution. Apart from a slight shift in the positions of the signals, there was no apparent doubling of signals, indicating the presence of a single enantiomer (see S28). Another ambiguity discovered during our research is the existence of a structurally different compound with the same name. In particular, the name “eugenin” was already introduced in 1946 as a derivative of chromenone.^17^

Compound **6** was identified as narcissidine, which was previously isolated from *Zephyranthes*,^2^ but a full NMR description was reported last year using DMSO-*d*_6._^18^ Since this solvent tends to be the last choice for analysis, we decided to compare all the ^1^H and ^13^C chemical shifts with our experimental data obtained from CDCl_3_ and CD_3_OD, which are more frequently chosen solvents, if solubility is possible (Table S3). UV, MS, and NMR data are provided in the SI, along with the ECD spectrum, which we publish for the first time (see S30–S36). Regarding compound **7** as norlycoramine and compound **8** as zephyranine C,^13^ the latter was reported in 2023 in a phytochemical study on *Z. candida* (Lindl.) Herb., but for compound **7**, which has not yet been isolated from the genus *Zephyranthes*, we found a far more complicated story considering its name when searching the scientific databases. Although this alkaloid was isolated already in 1956,^19^ and whose characterization has been described several times in the literature,^20−25^ yet in 2024, Chaichompoo and co-workers reported this structure as “a first naturally occurring alkaloid” and named it *N*-demethyldihydrogalanthamine in their extensive phytochemical work on *Crinum latifolium* L.^26^ Why first naturally occurring, one might ask? Because they listed only a reference to Philipova and co-workers who presented a synthesis of compound **7**. However, even that paper named this molecule norlycoramine, along with the IUPAC name.^25^

Our initial work on *Z. citrina* has already described compounds **9** and **10** as 10-deoxy-6-hydroxyhippeastidine and 6-hydroxyhippeastidine, respectively. However, two years later, the article by Zhan and co-workers shed the most light on determining the also absolute configuration of **9** and **10**, and, therefore, for **3** and **4** (see page S38–S39). Their article reports computed and experimental ECD spectra for new alkaloids zephyranine C-F,^13^ which might be related to hippeastidine derivatives (see SI). Our experimental ECD data for **9** and **10** showed the same appearances as zephyranines, with two negative Cotton effects at approximately 240 and 280 nm (see S23–S24). Therefore, based on ECD analysis, zephyranine E (**9**) was found to be the parent molecule of **4**, whose spectra had the very same pattern (S23). Unfortunately, we could not similarly verify **3** due to a lack of compound. Considering that these two compounds (**3**, **4**) have almost comparable values of rotation, we aim to describe the absolute configuration of **3** to be the same as that of **4**, i.e., 3*S*,4a*S*,6*S*,10b*S* (see all the facts summarized in the SI, page S38–S39).

### Biological Activity of Isolated Compounds

All compounds isolated from *Z. citrina* Baker in sufficient quantity were subjected to evaluation of their cytotoxicity (**1**, **3**–**27**). Our previous study presented 21 alkaloids to focus on their effect on the *h*AChE and *h*BChE enzymes, which identified narcielline (**18**) as the most promising alkaloid for Alzheimer’s disease research isolated from this plant so far (IC_50_*h*AChE = 18.66 ± 2.28 μM and IC_50_*h*BChE = 1.34 ± 0.31 μM).^27^Table S4 shows the inhibitory effect of compounds **1**–**8** on *h*AChE and *h*BChE. Only compound **7**, a derivative of the clinically approved drug galanthamine, showed a negligible inhibition of *h*AChE (IC_50_ = 154.53 ± 2.57 μM).
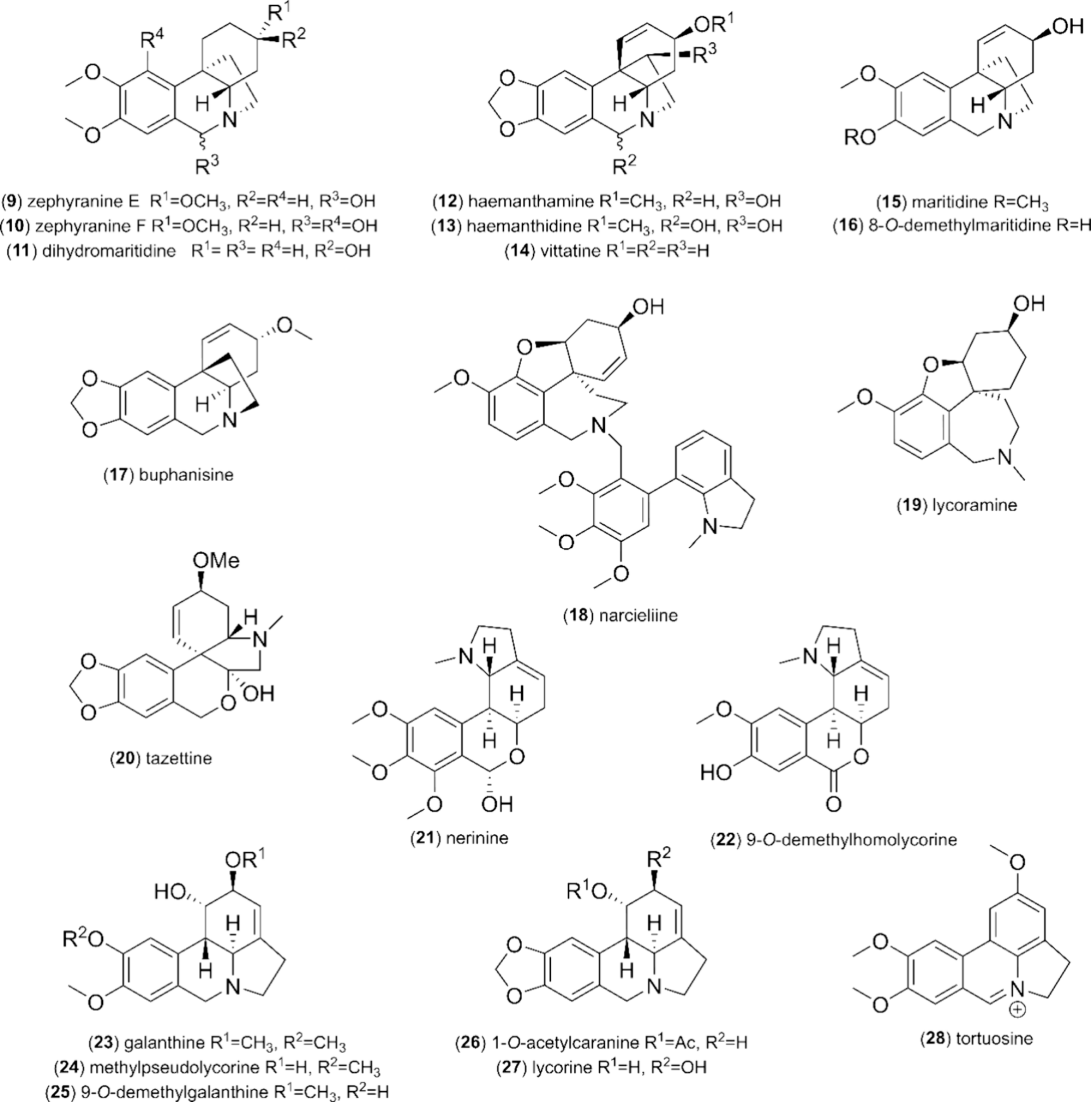


Concerning the cytotoxicity of alkaloids isolated from *Z. citrina*, initially, Prakash and Vedanayaki investigated the MeOH extract of the bulbs for its *in vitro* cytotoxic activity using the MTT assay against three human cancer cell lines from different tissue origins: cervical cancer (HeLa), breast cancer (MCF-7), and oral squamous carcinoma (SCC-9). The findings revealed that exposure for 24 h to MeOH bulb extract exhibited significant cytotoxic effects on the SCC-9 cell line, with an IC_50_ value of 88.79 μg/mL.^28^ In this context, and to assess potential negative effects on human cell cultures, compounds **1**, and **3**–**27** were screened in our study for cytotoxicity using a 10 μM single-dose regimen. The results were compared with those using the clinically approved antineoplastic agent doxorubicin at 1 μM. The effect on cell growth was investigated on nine human tumor and one nontumor cell lines, namely Jurkat (acute T-cell leukemia), MOLT-4 (acute lymphoblastic leukemia), A549 (lung carcinoma), HT-29 (colorectal adenocarcinoma), PANC-1 (pancreas epithelioid carcinoma), A2780 (ovarian carcinoma), HeLa (cervix adenocarcinoma), MCF-7 (breast adenocarcinoma), SAOS-2 (osteosarcoma) and MRC-5 (nontumor lung fibroblasts). Compounds **1**, **3**–**11**, and **14**–**26** did not show any considerable cytotoxicity (GP value > 50%) across the tested cell lines during the 48-h interval. No toxicity is valuable information, especially for compound **18**, as it is a potent inhibitor of *h*AChE and *h*BChE.^27^

Haemanthamine (**12**), haemanthidine (**13**), and lycorine (**27**) were the compounds which significantly exhibited cancer cells growth. Compound **27** showed a significant boost in antiproliferative activity against the human acute T-cell leukemia Jurkat (GP value 5%) cell culture model, maintaining a very strong inhibitory activity on HT-29 (GP value 5%) and MOLT-4 (GP value 9%) cells. Compound **13** demonstrated exceptionally high activity against HT-29 (GP value 7%) cells and MOLT-4 (GP value 11%). Furthermore, compounds **12**, **13**, and **27** exceeded the growth-inhibitory activity of doxorubicin applied at 1 μM. The AAs **12** and **27** exhibited similar cytotoxicity against all cell lines in the study, including nontumor lung fibroblasts (MRC-5), showing no signs of significant cytotoxic specificity.

Building upon our previous research determining the selectivity indexes (SI) for compounds, those for **12**, **13**, and **27** were obtained using the same panel of cell lines and under identical treatment period conditions.^29^ The calculated SI values ranged from 0.05 to 1.67 for compound **12**, from 0.14 to 1.08 for compound **13**, and from 0.62 to 1.14 for compound **27**. An SI value exceeding 2 indicates selective cytotoxic activity of the compound. Conversely, an SI value below 2 suggests a broader, nonselective cytotoxic effect on cells.^30^ Considering the determined SI values, **12**, **13**, and **27** had very low selectivity toward cancer cells.

To the best of our knowledge, the results of our cytotoxicity screening represent the first documentation of the bioactivity of certain AAs (**1**, **3**–**5**, **8**–**11**, **14, 18**, **21**, **22**, **24**) against selected cancer histotypes.

Furthermore, the cytotoxicity results of alkaloids that have already been published (**7**, **12**, **13**, **16**, **17**, **19**, **20**, **23, 25**–**27**) correlate with the results from this study, except for maritidine (**15**).^9,31−37^ Even though initial studies demonstrated that **15** exhibited pronounced *in vitro* antineoplastic activity against KB human oral epidermoid carcinoma, with ED_50_ values of 0.51 μg/mL, our results showed a lack of inhibitory activity for this alkaloid.^38^ Hence, the absence of data for certain less abundant AAs and the presence of such contradictory results emphasizes the necessity for further investigation into the bioactivity of lesser-known alkaloids that have received limited exploration thus far.

With 12 haemanthamine derivatives in hand (**1**, **3**, **4**, **8**–**16** - α-crinane scaffold), it is possible to unravel the structure–activity relationship since they are essentially homologues. Compound **12** is a potent inhibitor of the cell life cycle (see Table 2), but if the methylenedioxy group is replaced by either two methoxy groups (in **1**, **3**, **4**, **8**–**11**, **15**) or hydroxy and methoxy groups (**16**), then the inhibitory potency drops crucially. Also, the effect of a missing 11-OH and methyl substitution on 3-OH leads to the elimination of cytotoxicity (**14**). Interestingly, when position 6 in **12** is substituted by a hydroxyl (**13**), the effect gets only slightly affected (see Table 2). Another possible modification seems to be the configuration of 3-OH since crinamine (3-epimer of **12**) was reported to have cytotoxic properties.^39−41^ In 2009, Sun and co-workers isolated crinamine from bulbs of *Crinum asiaticum* L. var. *sinicum* Baker, and this alkaloid was tested for its cytotoxic activity against a mini-collection of human tumor cell lines by the standard MTT method after 72 h of exposure. In this study, crinamine exhibited a significant inhibitory effect on lung adenocarcinoma A549 cells, with an IC_50_ value of 15.9 ± 5.5 μg/mL, demonstrating comparable bioactivity to the 3-epimer, compound **12**.^41^ On the other hand, compound **17**, a structural type often referred to as β-crinane, showed a slight inhibition of growth of MOLT-4 and MCF-7 cell lines, even without an 11-OH group. The importance of the isolated double bond is not entirely clear, as only one study compared 1,2-unsaturated and 1,2-saturated derivatives (HL-60 and HSC-2 cells only), where unsaturated derivatives were significantly more effective.^42^ Apparently, cytotoxicity is quite closely related to the specific haemanthamine scaffold.

**Table 2:** Cytotoxicity Screening of Alkaloids Isolated from *Z. citrina* Baker^a^

compd^b^	Jurkat	MOLT-4	A549	HT-29	PANC-1	A2780	HeLa	MCF-7	SAOS-2	MRC-5
**1**	111	99	100	101	102	98	97	106	101	107
**2**	n.t.	n.t.	n.t.	n.t.	n.t.	n.t.	n.t.	n.t.	n.t.	n.t.
**3**	108	101	103	103	101	110	108	97	99	104
**4**	90	101	100	91	88	85	109	96	98	99
**5**	98	87	91	91	98	104	92	102	89	110
**6**	111	101	113	108	102	101	123	103	101	101
**7**	103	99	103	100	103	97	98	97	97	98
**8**	109	98	117	121	110	105	117	112	111	100
**9**	96	79	95	95	98	111	106	108	77	100
**10**	89	78	81	88	77	113	82	99	73	97
**11**	99	95	88	97	100	107	98	104	97	104
**12**	**18**	**6**	**33**	**5**	**37**	**38**	**16**	**17**	**34**	**32**
**13**	**23**	**11**	**38**	**7**	**38**	52	**25**	**24**	**35**	n.t.
**14**	91	92	80	84	84	98	100	79	79	83
**15**	104	100	95	93	91	94	101	98	94	104
**16**	102	110	106	103	101	101	114	105	98	97
**17**	52	32	60	71	77	55	75	**49**	76	82
**18**	103	95	110	106	111	88	90	115	106	111
**19**	102	105	96	83	102	92	82	101	97	n.t.
**20**	93	95	83	57	76	101	84	94	92	n.t.
**21**	91	93	101	83	90	95	112	92	105	101
**22**	100	100	109	106	92	94	96	102	103	93
**23**	98	103	93	64	97	113	93	114	97	n.t.
**24**	109	103	104	114	109	99	116	104	98	96
**25**	91	88	83	94	83	125	110	102	71	92
**26**	132	119	113	107	119	141	98	131	95	n.t.
**27**	**5**	**9**	**27**	**5**	**36**	**39**	**15**	**27**	**27**	**25**
**28**	n.t.	n.t.	n.t.	n.t.	n.t.	n.t.	n.t.	n.t.	n.t.	n.t.
doxorubicin^c^	0	0	66	77	59	5	7	41	73	40
DMSO^d^	113	115	106	87	95	98	89	99	102	n.t.

a The growth percent (GP) values from the intervals 0–49% of the proliferation decrease are highlighted in bold.

b *c* = 10 μM.

c A positive control in *c* = 1 μM.

d A 0.1% DMSO negative vehicle control; n.t. = not tested.

In summary, four undescribed compounds (**1**–**4**) were isolated from the EtOH extract prepared from fresh bulbs of *Z. citrina* Baker. Their structures were determined by spectroscopic analysis. In addition, NMR data and optical rotation were described for known compounds (**5**, **6**) that were not accurately reported in the literature. The acid–base conditions used during the extraction and separation process led to the formation of the isolation artifacts possessing either an ethyl in the hemiaminal ether (**3**, **4**) or hemiacetal ether (**5**) group. When searching for an accurate name for **3** and **4**, two options were found. A comparison of available data was the keystone for this decision. Furthermore, the problematic evolution of the name for compound **7** was discussed, revealing that recent data did not refer to the existing alkaloid, which has been known for quite a long time.

After identifying the compounds’ structures, the newly isolated compounds **1**–**8** were tested for their effect on human cholinesterases. However, no significant inhibition of either *h*AChE or *h*BChE was detected (IC_50_ > 100 μM).

Then, all compounds isolated from *Z. citrina* in this project were screened for inhibition of the growth of nine tumor and one nontumor cell lines. The screening brought new data for ten known, not previously tested compounds. Unfortunately, no new cytotoxic alkaloid was identified, as the most active alkaloids were haemanthamine (**12**) and haemanthidine (**13**) (α-crinane type of AAs), along with lycorine (**27**), which are notoriously known cytotoxic Amaryllidaceae alkaloids. Thanks to all other screened alkaloids from the α-crinane group (**1**, **3**, **4**, **8**–**16**), it was possible to define the scaffold maintaining cytotoxicity. The tetrahydroisoquinoline must be fused with dioxole (also characteristic of structures of lycorine and pancratistatin), and the C-11 should bear a hydroxyl group. Then, a hydroxyl at position 6 introduces a labile hemiaminal ether, but does not significantly affect the cytotoxic effect. Our structure–activity relationship summary reports that almost any intervention in the structure of **12** leads to a loss of cytotoxicity.

## Experimental Section

### General Experimental Procedures

Optical rotation was measured on a KRÜSS optronic P3000 automatic polarimeter. The UV spectra were analyzed using a Waters’ high-performance liquid chromatography system (Milford, USA). The HPLC apparatus consisted of Waters Sample Manager 2767, System Fluidics organizer, Waters 2545 Binary Gradient module, Waters 2998 Photodiode array detector, and Waters Acquity qDa detector. Samples were analyzed at ambient temperature using an XSelect CSH C18 OBD reverse phase column (100 mm × 4.6 mm i.d., 5 μm) (Milford, USA). The ECD spectra were recorded on a JASCO J-815 CD spectrometer in MeOH. The NMR experiments were recorded on a VNMR S500 (Varian) spectrometer at 500 MHz for proton nuclei and 125.7 MHz for carbon nuclei at 25 °C. CD_3_OD was referenced to 3.30 ppm for ^1^H NMR data and 49.0 ppm for ^13^C NMR data, and CDCl_3_ was referenced to 7.27 ppm for ^1^H NMR data and 77.0 ppm for ^13^C NMR data. The HRESIMS were acquired using an Acquity UPLC-I Class UHPLC system (Waters, Milford, USA) coupled with a Synapt G2-Si high-resolution mass spectrometer (Waters, Manchester, UK) based on a Q-TOF platform. Chromatographic separation was carried out by gradient elution on a BEH C18 analytical column (2.1 mm × 50 mm, 1.7 μM) and a mobile phase composition of 0.1% formic acid in water (A) and acetonitrile (B), with a flow rate 0.4 mL/min. Electrospray ionization was set up in positive mode. The HRESIMS were acquired in the mass range of 50–1200 *m*/*z* using leucine-enkephalin for internal and sodium formate for external calibration.

### Plant Material

The fresh bulbs of *Z. citrina* Baker were obtained from the herbal distributor Lukon Glads (Sadská, Czech Republic). Botanical identification was performed by Professor Lubomír Opletal. A voucher specimen is deposited in the Herbarium of the Faculty of Pharmacy in Hradec Králové under number: CUFPH-16130/AL-212.^27^

### Extraction and Isolation

Fresh bulbs (35 kg) were minced and extracted with EtOH (96%, v/v, 3×) under reflux for 30 min. The extract was filtered and evaporated to dryness under reduced pressure. The crude extract (485 g) was acidified to pH 1–2 with 5% HCl (7 L) and the volume of the suspension was made up to 10 L with H_2_O. The suspension was filtered, and the pH of the filtrate was adjusted to 9–10 with a 10% solution of Na_2_CO_3_ and extracted with CHCl_3_ (3 × 12 L). The organic layer was evaporated to give 312 g of dark brown residue. This alkaloid summary extract was again dissolved in 2% HCl (5 L), defatted with Et_2_O (3 × 3 L) and the pH was adjusted to 9–10 with 10% Na_2_CO_3_. The H_2_O layer was extracted with EtOAC (4 × 5 L), and CHCl_3_ (2 × 4 L). Both Dragendorff positive parts were evaporated and pooled together. A concentrated alkaloid extract (151 g) in the form of brown syrup was obtained. This alkaloidal residue was subjected to column chromatography, and subsequent chromatographic techniques led to the isolation of 21 alkaloids.^27^

For the purposes of the presented study, the remaining unprocessed alkaloidal material (approximately 5 g) was used for further isolation of potentially bioactive compounds. These residues, consisting mainly of mother liquors and unused subfractions, were pooled together after the first isolation study and subjected to further open column chromatography on aluminum oxide (Al_2_O_3_; 200 g), with a similar gradient elution as previously described.^27^ Collected 50 mL fractions were combined based on their similar TLC and GC-MS profiles, yielding **fractions A–F** (3.3 g). To isolate further compounds, these fractions were purified using various chromatographic and crystallization techniques described in the following paragraphs.

**Fraction A** (220 mg) was separated by preparative TLC (petroleum ether–CHCl_3_–Et_2_NH, 90:30:5, v/v), yielding 11 mg of 6-*O*-ethylzephyranine F (**3**), and 9.5 mg of 6-*O*-ethylzephyranine E (**4**), isolation artifacts.

**Fraction B** (530 mg) was subjected to initial separation by prep. TLC (Me_2_CO–MeOH–NH_4_OH, 90:7:3, v/v). After purification in another mobile phase (EtOAc–MeOH–H_2_O, 85:15:10, v/v), 60 mg of norlycoramine (**7**) and 87 mg of zephycitrine (**1**) were obtained.

**Fraction C** (339 mg) was initially purified by prep. TLC (cyclohexane–EtOAc– Et_2_NH, 30:60:10, v/v). The obtained alkaloidal residue was crystallized in EtOH, leading to a yield of 68.3 mg of narcissidine (**6**). This alkaloid was also obtained in our previous study.^27^

**Fraction D** (288.8 mg) was separated by prep. TLC (EtOH–MeOH– Et_2_NH, 90:10:3, v/v) into two subfractions, D1 and D2. Subfraction D2 (25 mg), after purification by TLC (ACN–MeOH–TFA, 40:10:0.1), gave a yield of 2.1 mg of an undescribed compound, 6-oxonarcissidine (**2**).

**Fraction E** (540 mg) was separated by prep. TLC (cyclohexane–Me_2_CO–NH_4_OH, 10:90:2, × 2, v/v) resulting in two alkaloidal compounds. The first was purified by recrystallization from EtOAc to obtain 210 mg of narcissidine (**6**), while the second was purified by prep. TLC using the same mobile phase to yield 15 mg of eugenine (**5**).

**Fraction F** (1.4 g), after purification by prep. TLC (toluene–EtOAc– Et_2_NH, 60:30:10, × 2, v/v) yielded 860 mg of zephyranine C (**8**), as a mixture of 6-epimers (*dr* 75:25).

#### Zephycitrine (**1**)

yellow-brown powder; [α]_D_^24^ = +46 (*c* 0.62, CHCl_3_); for ^1^H and ^13^C NMR data see Table 1; HRESIMS *m*/*z* 318.1710 [M + H]^+^ (calcd for C_18_H_24_NO_4_^+^, 318.1700). See Supplementary data for HRESIMS and NMR spectra, pages S4–S7.

#### 6-Oxonarcissidine (**2**)

white crystals; [α]_D_^24^ = −84 (*c* 0.10, MeOH); UV (MeOH) λ_max_ = 223, 265, 301 nm; for ^1^H and ^13^C NMR data see Table 1; HRESIMS *m*/*z* 348.1446 [M + H]^+^ (calcd for C_18_H_22_NO_6_^+^, 348.1441). See SI for UV, HRESIMS, and NMR spectra, pages S9–S12.

#### 6-*O*-Ethylzephyranine F (**3**)

pale white amorphous solid; [α]_D_^24^ = +44 (*c* 0.10, MeOH); UV (MeOH) λ_max_ = 244, 283 nm; for ^1^H and ^13^C NMR data see Table 1; HRESIMS *m*/*z* 364.2120 [M + H]^+^ (calcd for C_20_H_30_NO_5_^+^, 364.2119). See SI for UV, HRESIMS, and NMR spectra, pages S14–S17.

#### 6-*O*-Ethylzephyranine E (**4**)

pale white amorphous solid; [α]_D_^24^ = +47 (*c* 0.23, MeOH); UV (MeOH) λ_max_ = 242, 281 nm; ECD (0.1 mg/ml, MeOH): Δε_241_ = – 1.94, Δε_277_ = – 1.76; for ^1^H and ^13^C NMR data see Table 1; HRESIMS *m*/*z* 348.2177 [M + H]^+^ (calcd for C_20_H_30_NO_4_^+^, 348.2170). See SI for NMR, HRESIMS, and ECD spectra, pages S18–S23.

#### Eugenine (**5**)

yellowish powder; [α]_D_^24^ = +113 (*c* 0.18, CHCl_3_); UV(MeOH) λ_max_ = 237, 283 nm; for ^1^H and ^13^C NMR data see Table 1; HRESIMS *m*/*z* 332.1868 [M + H]^+^ (calcd for C_19_H_26_NO_4_^+^, 332.1856). See SI for HRESIMS and NMR spectra, pages S25–S29.

#### Narcissidine (**6**)

colorless crystals; [α]_D_^24^ = −56 (*c* 0.10, MeOH); UV (MeOH) λ_max_ = 237, 280 nm; ECD (0.1 mg/ml, MeOH): Δε_231_ = – 8.92; for ^1^H and ^13^C NMR data see Table S3; HRESIMS *m*/*z* 334.1650 [M + H]^+^ (calcd for C_18_H_24_NO_5_^+^, 334.1649). See SI for UV, ECD, NMR, HRESIMS data, pages S30–S36.

## Biological Assays

### Cytotoxicity

#### Cell Culture and Culture Conditions

Selected human tumor and nontumor cell lines Jurkat (acute T-cell leukemia), MOLT-4 (acute lymphoblastic leukemia), A549 (lung carcinoma), HT-29 (colorectal adenocarcinoma), PANC-1 (pancreas epithelioid carcinoma), A2780 (ovarian carcinoma), HeLa (cervix adenocarcinoma), MCF-7 (breast adenocarcinoma), SAOS-2 (osteosarcoma) and MRC-5 (nontumor lung fibroblasts) were purchased from European Collection of Authenticated Cell Cultures (ECACC, Salisbury, UK) and cultured according to the provider’s culture method guidelines. All cell lines were maintained at 37 °C in a humidified 5% carbon dioxide and 95% air incubator. The cells in low passage number (nontumor primary cell line MRC-5 was used for a maximum of 10 passages and cancer cell lines were used for a maximum of 20 passages) and in an exponential growth phase were used for this study.

#### WST-1 Cell Proliferation Assay and Growth Percent Calculation

Each cell line was seeded at a previously established optimal density (1 × 10^3^ to 50 × 10^3^ cells per well) in a 96-well plate (TPP, Trasadingen, Switzerland) and cells were allowed to settle overnight. During screening tests for cytotoxicity, cells were treated with alkaloids at a final concentration of 10 μM for 48 h. Doxorubicin (Sigma-Aldrich, St. Louis, USA), at a concentration of 1 μM, was used as a positive control. At the end of the culture period, the WST-1 proliferation assay (Roche, Basel, Switzerland) was performed according to the manufacturer’s protocol. The absorbance was determined using a Tecan Spark microplate reader (Tecan, Männedorf, Switzerland). Each value is the mean of three independent experiments and represents the percentage of the proliferation of control, nontreated cells (100%). The growth percent (GP) value as the mean of the proliferation decrease was calculated for each cell line and derivative tested.

#### *h*AChE and *h*BChE Inhibition Assay

The *h*AChE and *h*BChE activities were determined using a modified method of Ellman, as described by Hrabinova and co-workers against recombinant AChE and recombinant BChE (purchased from University Hradec Kralove, Department of Chemistry, Faculty of Science).^43^ Other used chemicals were phosphate buffer solution (PBS, pH = 7.4), 5,5′-dithio-bis(2-nitrobenzoic) acid (Ellman’s reagent, DTNB), acetylthiocholine iodide (ATChI), butyrylthiocholine iodide (BTChI), and dimethyl sulfoxide (DMSO) (all chemicals were purchased from Sigma-Aldrich, Prague, Czech Republic). The results are expressed as IC_50_ (the concentration of the compound that is required to reduce 50% of cholinesterase activity).

Briefly, 8.3 μL of the corresponding enzyme, 283 μL of 5 mM DTNB, and 8.3 μL of the sample dilution in DMSO in different concentrations were added to the microplate. These solutions were incubated for 5 min at 37 °C. Then, 33.3 μL of 10 mM substrate (ATChI or BTChI) was added to initiate the reaction, and the whole reaction mixture was incubated for 2 min at 37 °C. After 2 min, the increase of absorbance (ΔA) at 412 nm was measured for 1 min at 37 °C using a spectrophotometer (SynergyTM HT Multi-Detection Microplate Reader). This difference after 1 min was used to calculate the percentage of inhibitory potential of the given derivative. Each measurement was repeated three times for every concentration of the compound. 8.3 μL DMSO was used as a blank. The % inhibition was calculated according to the following formula:
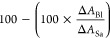
where Δ*A*_Bl_ is the increase of absorbance of the blank sample and Δ*A*_Sa_ is the increase of absorbance of the measured sample.

## Data Availability

The NMR data for **1**–**6** have been deposited in the Natural Products Magnetic Resonance Database (NP-MRD; www.np-mrd.org) and can be found at NP0333726 (compound **1**), NP0333727 (compound **2**), NP0333728 (compound **3**), NP0333729 (compound **4**), and NP0333730 (compound **5**), NP0066777 (compound **6**).

## Supporting Information Available

The UV, ECD, NMR and HRESIMS data of compounds (PDF)
