# Supplementary material for: Undescribed Amaryllidaceae Alkaloids from Zephyranthes citrina and Their Cytotoxicity
Source: J Nat Prod. 2024 Sep 4;87(9):2317–26. doi: 10.1021/acs.jnatprod.4c00825 (PMC11443518; doi:10.1021/acs.jnatprod.4c00825)
Supplement: Supplementary file 1 — np4c00825_si_001.pdf [file np4c00825_si_001.pdf]

Supporting Information for:

Undescribed Amaryllidaceae Alkaloids from *Zephyranthes citrina* and their Cytotoxicity

Jana Křoustková,[a,*] Eliška Kohelová,[a] Darina Muthná,[b] Jiří Kuneš,[c] Radim Havelek,[b] Rudolf Vrabec,[a] Milan Malaník,[d] Daniela Suchánková,[a] Jakub Chlebek,[a] Jaroslav Jenčo,[a] Štefan Kosturko,[a] Lucie Cahlíková[a]

[a]*Department of Pharmacognosy and Pharmaceutical Botany,* [c]*Department of Bioorganic and Organic Chemistry, Faculty of Pharmacy, Charles University, Heyrovského 1203, 500 03 Hradec Králové, Czech Republic*

[b]*Department of Medical Biochemistry, Faculty of Medicine in Hradec Králové, Charles University, Šimkova 870, Hradec Králové 500 03, Czech Republic*

[d]*Department of Natural Drugs, Faculty of Pharmacy, Masaryk University, Brno, Czech Republic*

*Corresponding author:

PharmDr. Jana Křoustková, Ph.D.

E-mail address: marikoj2@faf.cuni.cz

Orcid ID: 0000-0001-9758-2067

Table of contents

HRESIMS spectrum of zephycitrine (**1**) ... S4

^{1}H NMR spectrum (500 MHz) of zephycitrine (**1**) in $CDCl_3$ ... S4

^{13}C NMR spectrum (125.7 MHz) of zephycitrine (**1**) in $CDCl_3$ ... S4

HSQC spectrum of zephycitrine (**1**) in $CDCl_3$ ... S5

COSY spectrum of zephycitrine (**1**) in $CDCl_3$ ... S5

H2BC spectrum of zephycitrine (**1**) in $CDCl_3$ ... S6

HMBC spectrum of zephycitrine (**1**) in $CDCl_3$ ... S6

NOESY spectrum of zephycitrine (**1**) in $CDCl_3$ ... S7

Table S1. Comparison of ^{1}H and ^{13}C NMR chemical shifts ($CDCl_3$) and optical rotation of zephycitrine (**1**) and haemanthamine (**12**) ... S8

HRESIMS spectrum of 6-oxonarcissidine (**2**) ... S9

^{1}H NMR spectrum (500 MHz) of 6-oxonarcissidine (**2**) in $CDCl_3$ ... S9

^{13}C NMR spectrum (125.7 MHz) of 6-oxonarcissidine (**2**) in $CDCl_3$ ... S9

HSQC spectrum of 6-oxonarcissidine (**2**) in $CDCl_3$ ... S10

COSY spectrum of 6-oxonarcissidine (**2**) in $CDCl_3$ ... S10

H2BC spectrum of 6-oxonarcissidine (**2**) in $CDCl_3$ ... S11

HMBC spectrum of 6-oxonarcissidine (**2**) in $CDCl_3$ ... S11

NOESY spectrum of 6-oxonarcissidine (**2**) in $CDCl_3$ ... S12

UV spectrum of 6-oxonarcissidine (**2**) in MeOH ... S12

Table S2. Comparison of ^{1}H and ^{13}C NMR chemical shifts ($CDCl_3$) and optical rotation of 6-oxonarcissidine (**2**), 1-*O*-acetyl-3-*O*-methyl-6-oxonarcissidine (**2a**), and narcissidine (**6**) ... S13

HRESIMS spectrum of 6-*O*-ethylzephyranine F (**3**) ... S14

^{1}H NMR spectrum (500 MHz) of 6-*O*-ethylzephyranine F (**3**) in $CDCl_3$ ... S14

^{13}C NMR spectrum (125.7 MHz) of 6-*O*-ethylzephyranine F (**3**) in $CDCl_3$ ... S14

HSQC spectrum of 6-*O*-ethylzephyranine F (**3**) in $CDCl_3$ ... S15

COSY spectrum of 6-*O*-ethylzephyranine F (**3**) in $CDCl_3$ ... S15

H2BC spectrum of 6-*O*-ethylzephyranine F (**3**) in $CDCl_3$ ... S16

HMBC spectrum of 6-*O*-ethylzephyranine F (**3**) in $CDCl_3$ ... S16

NOESY spectrum of 6-*O*-ethylzephyranine F (**3**) in $CDCl_3$ ... S17

UV spectrum of 6-*O*-ethylzephyranine F (**3**) in MeOH ... S17

HRESIMS spectrum of 6-*O*-ethylzephyranine E (**4**) ... S18

^{1}H NMR spectrum (500 MHz) of 6-*O*-ethylzephyranine E (**4**) in $CDCl_3$ ... S18

^{13}C NMR spectrum (125.7 MHz) of 6-*O*-ethylzephyranine E (**4**) in $CDCl_3$ ... S18

HSQC spectrum of 6-*O*-ethylzephyranine E (**4**) in $CDCl_3$ ... S19

COSY spectrum of 6-*O*-ethylzephyranine E (**4**) in $CDCl_3$ ... S19

H2BC spectrum of 6-*O*-ethylzephyranine E (**4**) in $CDCl_3$ S20
HMBC spectrum of 6-*O*-ethylzephyranine E (**4**) in $CDCl_3$ S20
NOESY spectrum of 6-*O*-ethylzephyranine E (**4**) in $CDCl_3$ S21
UV spectrum of 6-*O*-ethylzephyranine E (**4**) in MeOH S22
ECD spectra of 6-*O*-ethylzephyranine E (**4**), zephyranine C (**8**), zephyranine E (**9**), and zephyranine F (**10**) in MeOH S23
HRESIMS spectrum of eugenine (**5**) S25
^{1}H NMR spectrum (500 MHz) of eugenine (**5**) in CD_3OD S25
^{13}C NMR spectrum (125.7 MHz) of eugenine (**5**) in CD_3OD S25
HSQC spectrum of eugenine (**5**) in CD_3OD S26
COSY spectrum of eugenine (**5**) in CD_3OD S26
H2BC spectrum of eugenine (**5**) in CD_3OD S27
HMBC spectrum of eugenine (**5**) in CD_3OD S27
NOESY spectrum of eugenine (**5**) in CD_3OD S28
Experiment with eugenine (**5**) and Pirkel´s reagent in $CDCl_3$ S28
UV spectrum of eugenine (**5**) in MeOH S29
HRESIMS spectrum of narcissidine (**6**) S30
^{1}H NMR spectrum (500 MHz) of narcissidine (**6**) in $CDCl_3$ S30
^{13}C NMR spectrum (125.7 MHz) of narcissidine (**6**) in $CDCl_3$ S30
HSQC spectrum of narcissidine (**6**) in $CDCl_3$ S31
^{1}H NMR spectrum (500 MHz) of narcissidine (**6**) in CD_3OD S32
^{13}C NMR spectrum (125.7 MHz) of narcissidine (**6**) in CD_3OD S32
HSQC spectrum of narcissidine (**6**) in CD_3OD S33
COSY spectrum of narcissidine (**6**) in CD_3OD S33
H2BC spectrum of narcissidine (**6**) in CD_3OD S34
HMBC spectrum of narcissidine (**6**) in CD_3OD S34
NOESY spectrum of narcissidine (**6**) in CD_3OD S35
UV spectrum of narcissidine (**6**) in MeOH S35
ECD spectrum of narcissidine (**6**) in MeOH S36
Table S3. Narcissidine: A comparison of NMR data measured in CD_3OD, $CDCl_3$, and those found in literature S37
Data summary for compounds structurally related to **3**, **4**, **9**, and **10** S38
Table S4. Screening of compounds **1**–**8** for their cholinesterase inhibition S40
References S41

HRESIMS spectrum of zephycitrine (1)

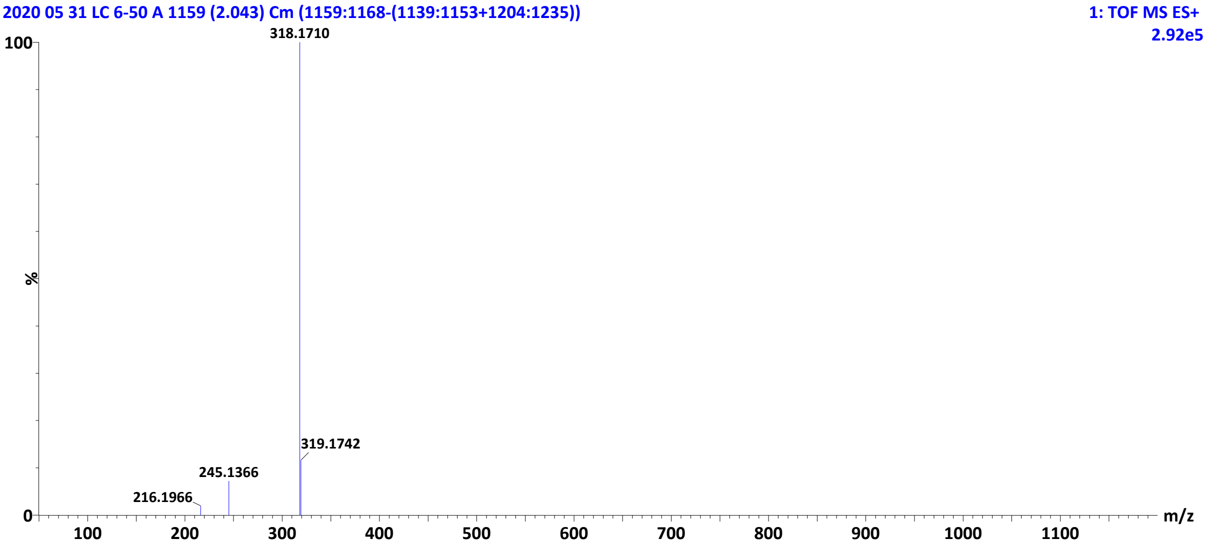

^{1}H NMR spectrum (500 MHz) of zephycitrine (1) in $CDCl_3$

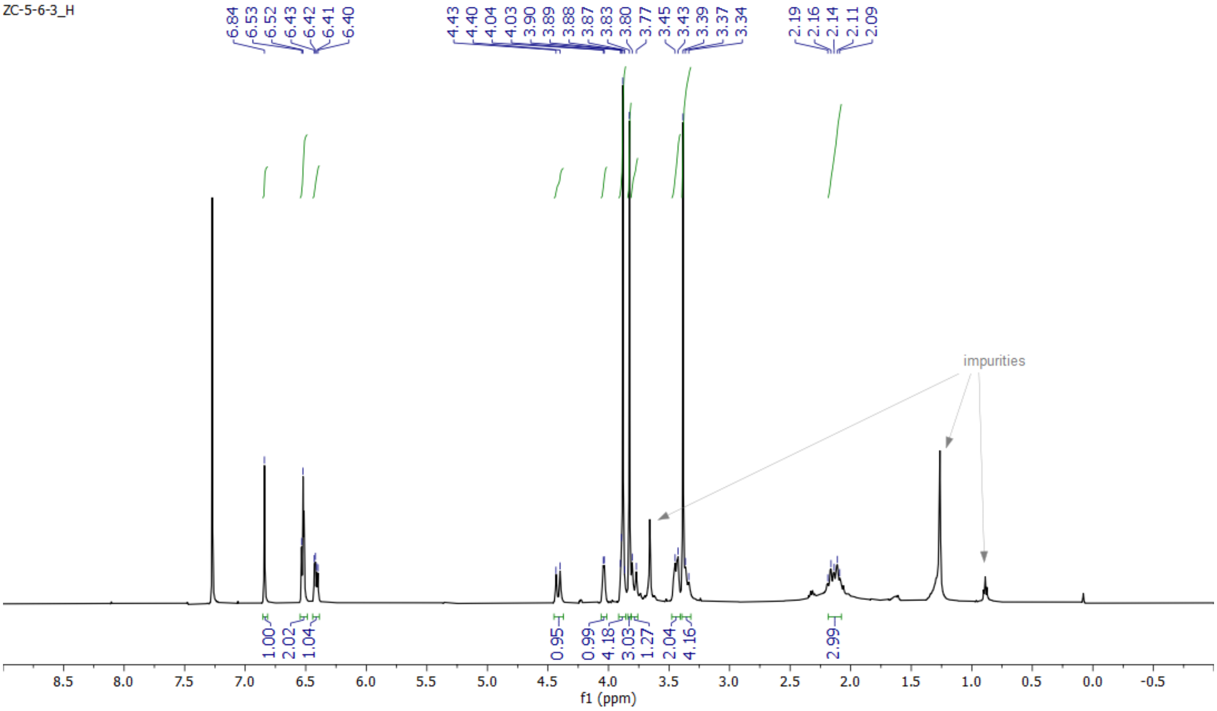

^{13}C NMR spectrum (125.7 MHz) of zephycitrine (1) in $CDCl_3$

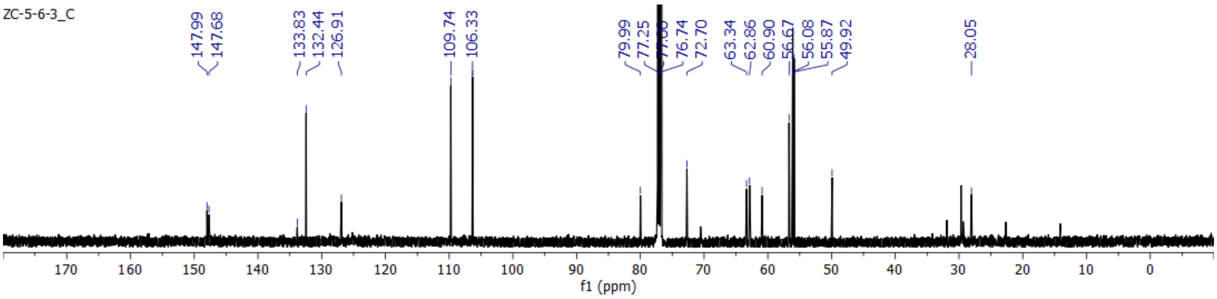

HSQC spectrum of zephycitrine (1) in $CDCl_3$

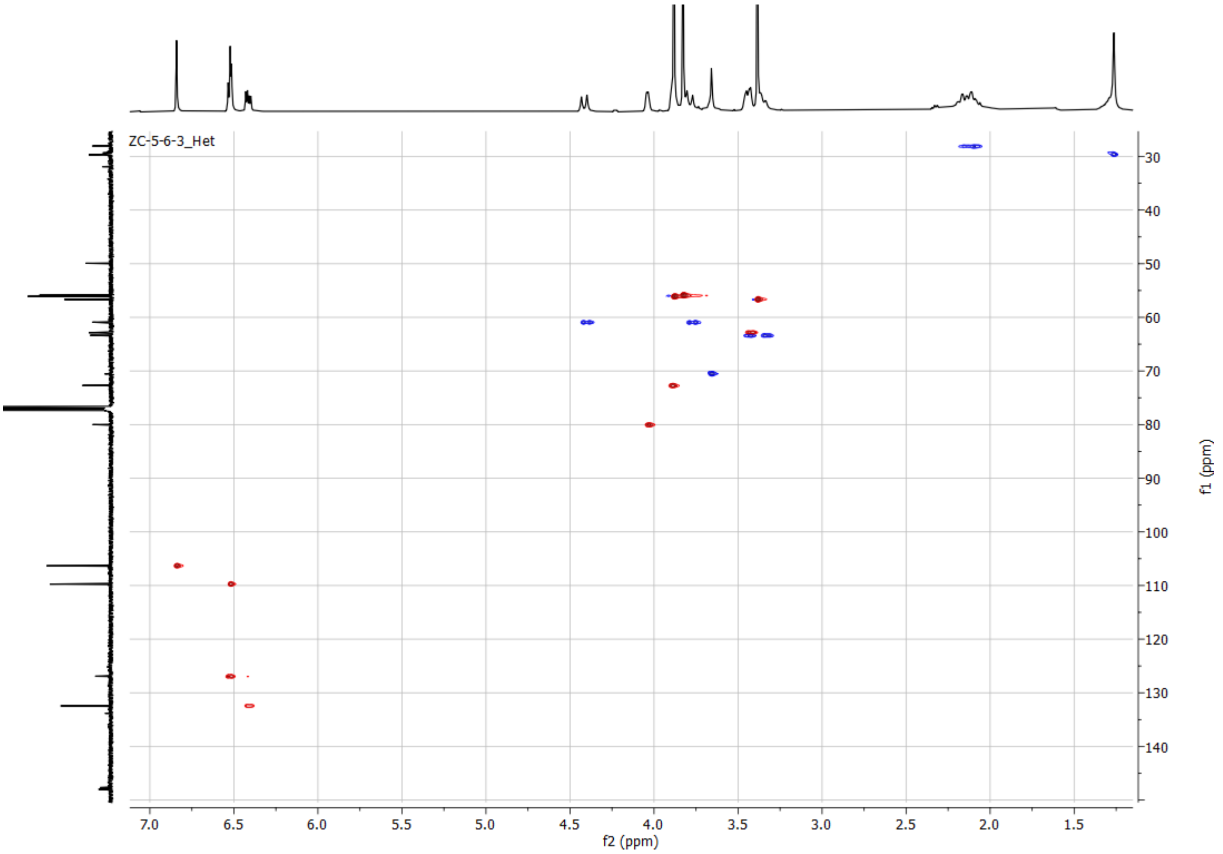

COSY spectrum of zephycitrine (1) in $CDCl_3$

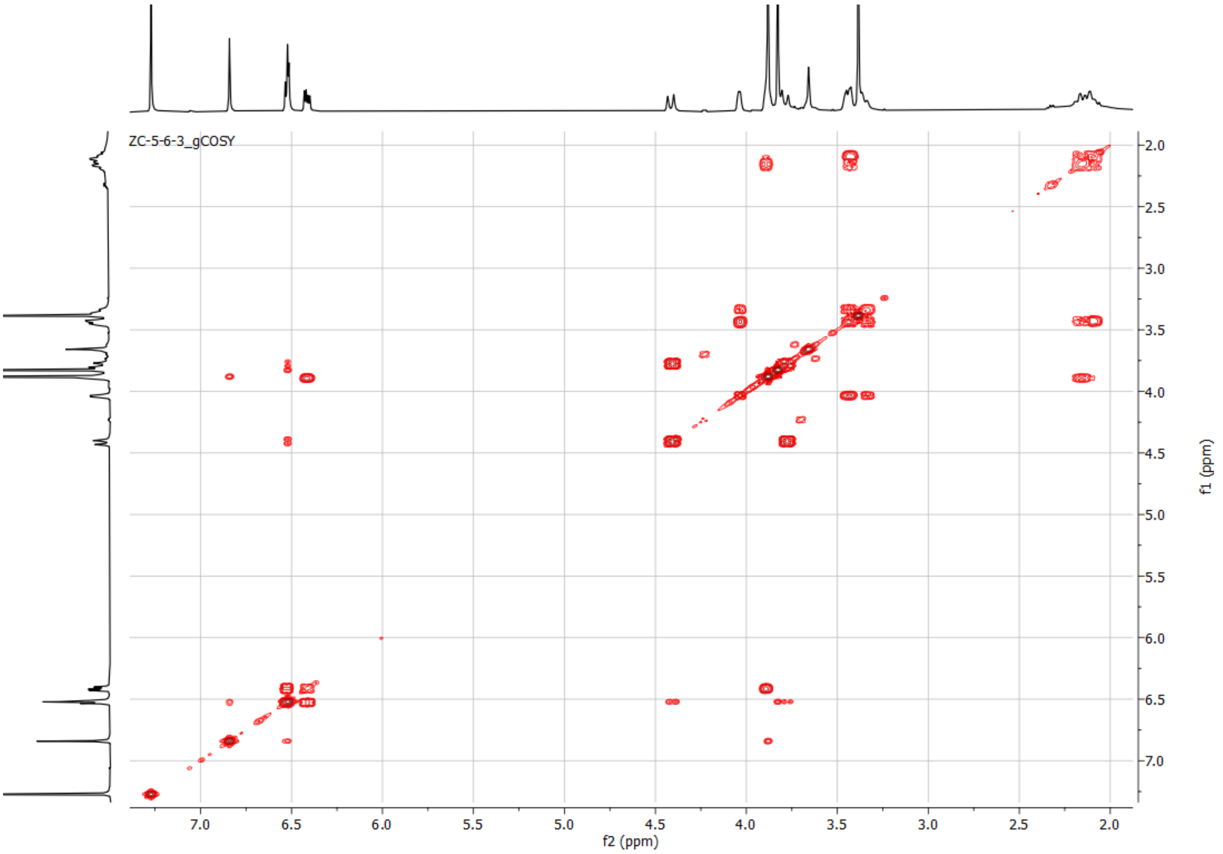

H2BC spectrum of zephycitrine (1) in $CDCl_3$

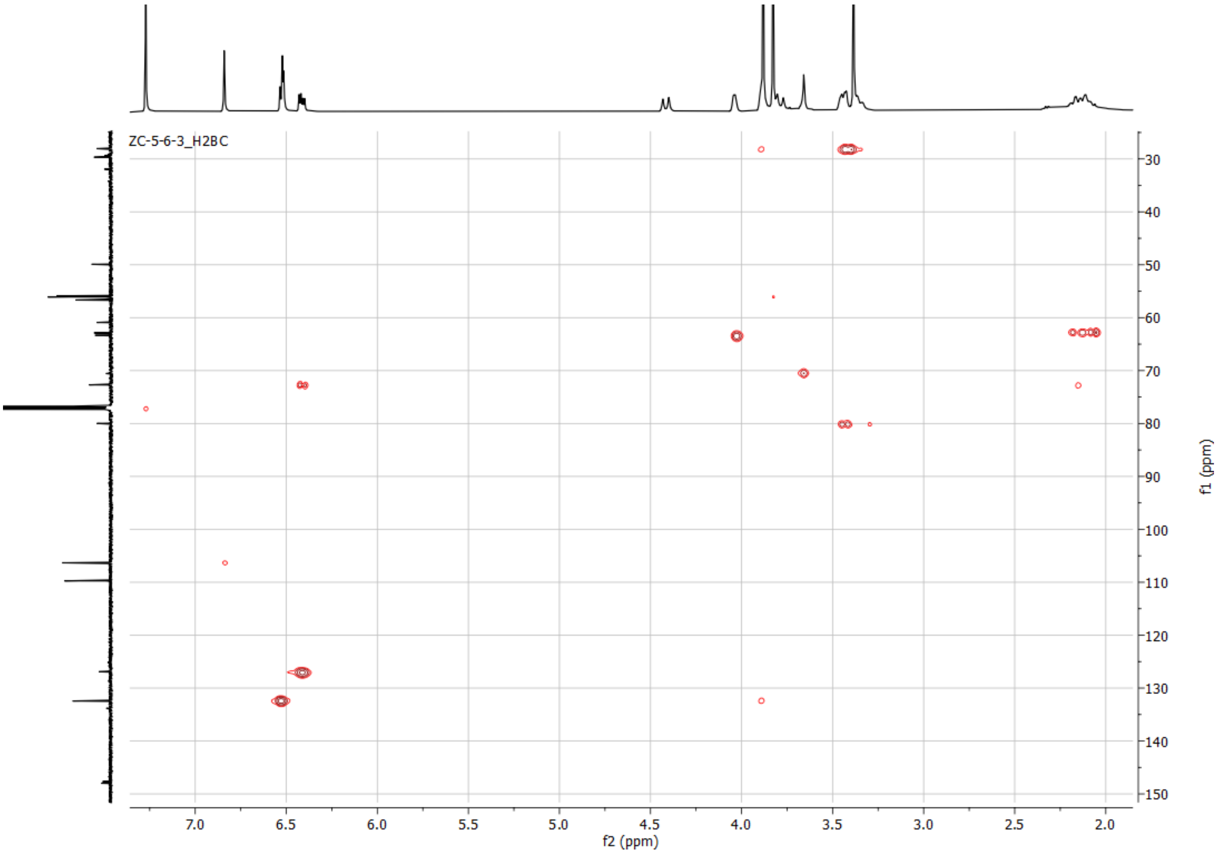

HMBC spectrum of zephycitrine (1) in $CDCl_3$

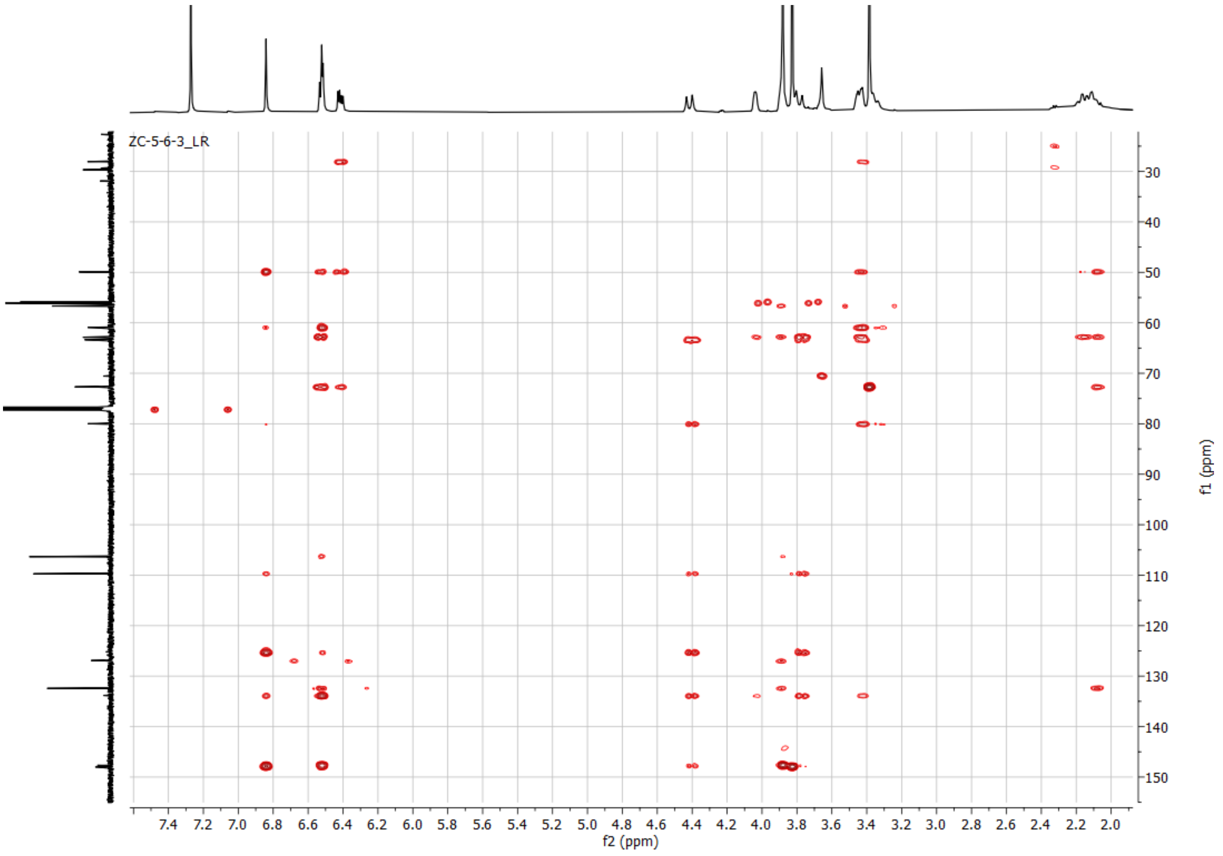

NOESY spectrum of zephycitrine (1) in $CDCl_3$

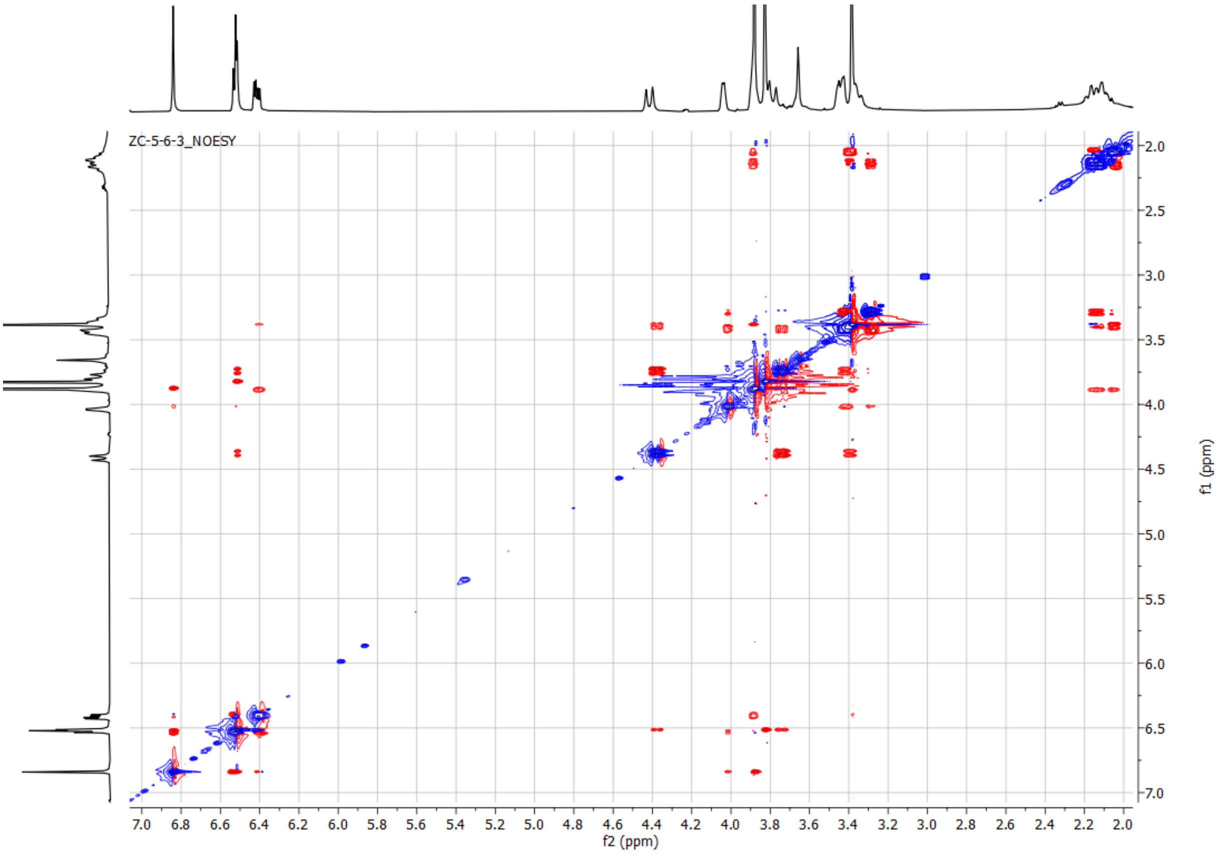

Table S1. Comparison of ^{1}H and ^{13}C NMR chemical shifts ($CDCl_3$) and optical rotation of zephycitrine (**1**) and haemanthamine (**12**) [1]

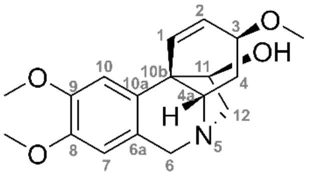

1

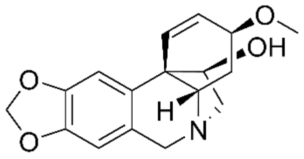

12

	**1**		**12**	
**No.**	δ_C, type	δ_H, mult. (J in Hz)	δ_C, type	δ_H, mult. (J in Hz)
1	126.9, CH	6.52, d, overlap (10.0)	127.3, CH	6.43, d (10.2)
2	132.4, CH	6.41, dd (10.0, 4.9)	132.4, CH	6.36, dd (10.2, 4.7)
3	72.7, CH	3.91–3.86, m, overlap	72.7, CH	3.86, ddd (4.7, 4.0, 1.8)
4	28.1, CH_2	2.22–2.03, m	28.2, CH_2	2.11, ddd (13.8, 5.4, 1.8)<br>2.06–1.97, m
4a	62.9, CH	3.48–3.41, m	62.6, CH	3.43–3.30, m
6	60.9, CH_2	4.42, d (16.5)<br>3.79, d (16.5)	61.3, CH_2	4.32, d (17.1)<br>3.69, d (17.1)
6a	125.2, C		126.7, C	
7	109.7, CH	6.52, s, overlap	106.8, CH	6.47, s
8	148.0, C		146.4, C	
9	147.9, C		146.1, C	
10	106.3, CH	6.84, s	103.3, CH	6.82, s
10a	133.8, C		135.3, C	
10b	49.9, C		50.0, C	
11	80.0, CH	4.06–4.01, m	80.1, CH	3.98, dd (6.7, 3.3)
12	63.3, CH_2	3.48–3.41, m<br>3.40–3.35, m	63.5, CH_2	3.43–3.30, m<br>3.25 dd, (14.1, 3.3)
3-OCH_3	56.7, CH_3	3.38, s, overlap	56.5, CH_3	3.36, s
8-OCH_3	55.9, CH_3	3.83, s	-	-
9-OCH_3	56.1, CH_3	3.88, s, overlap	-	-
-OCH_2O-	-	-	100.8, CH_2	5.89, bs; 5.88, bs
$[\alpha]_D$	+46 ° (c 0.20, $CHCl_3$, 23 °C)		+41 ° (c 0.62, $CHCl_3$, 24 °C)	

HRESIMS spectrum of 6-oxonarcissidine (2)

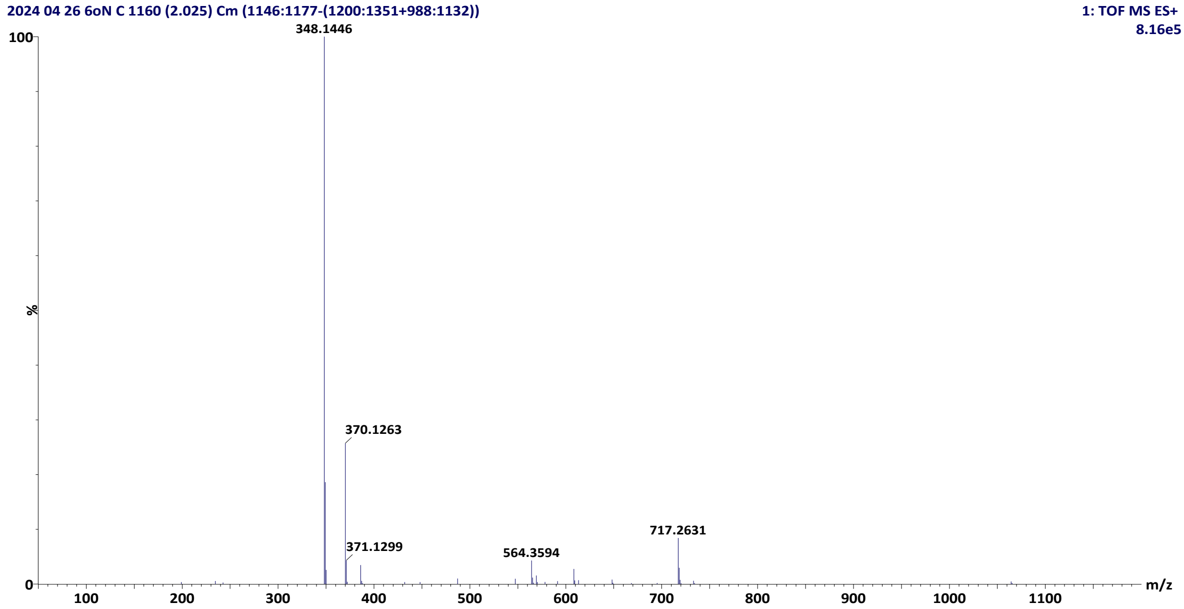

^{1}H NMR spectrum (500 MHz) of 6-oxonarcissidine (2) in $CDCl_3$

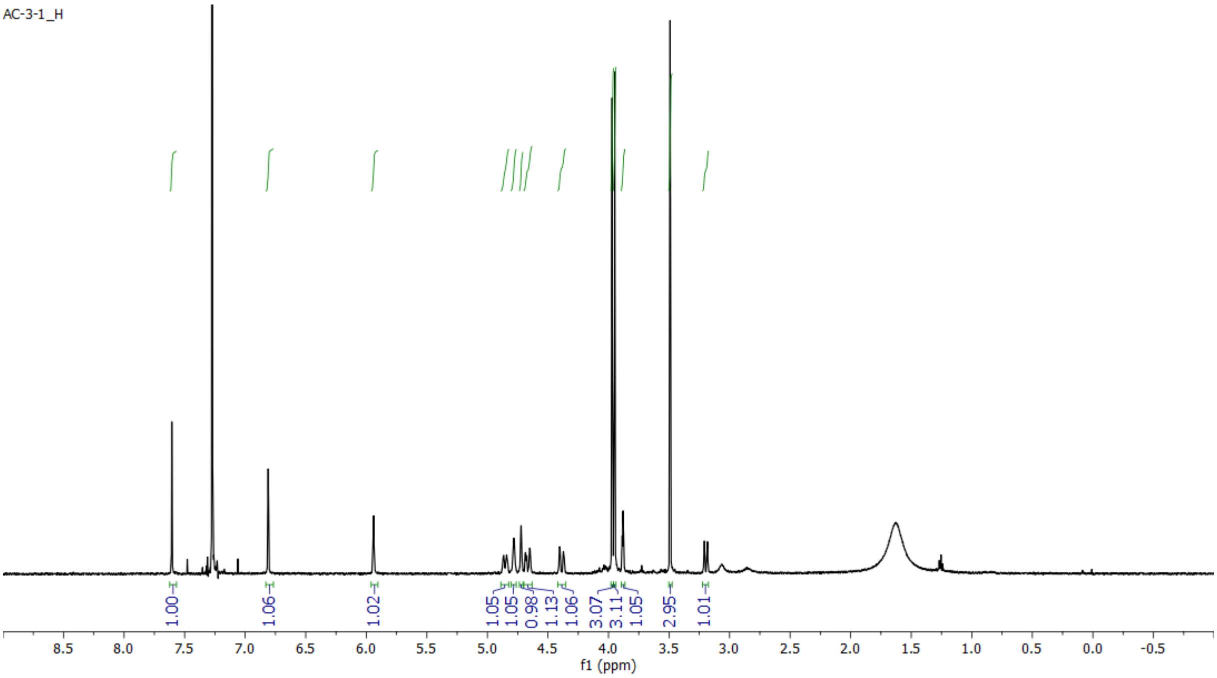

^{13}C NMR spectrum (125.7 MHz) of 6-oxonarcissidine (2) in $CDCl_3$

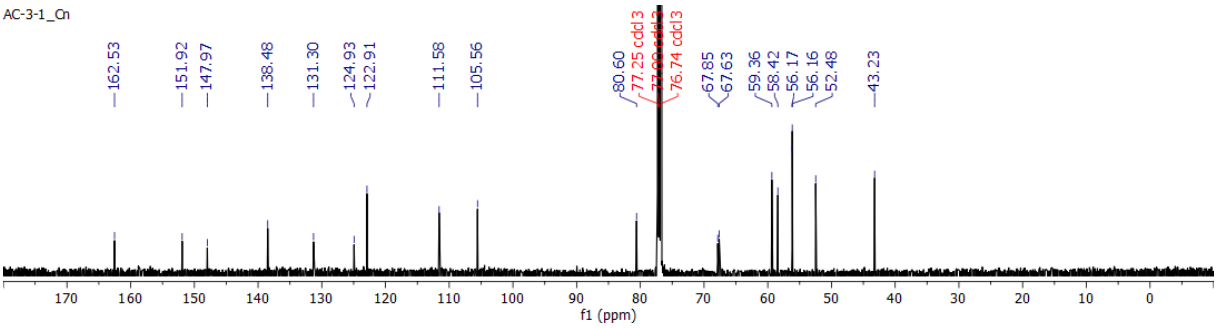

HSQC spectrum of 6-oxonarcissidine (2) in $CDCl_3$

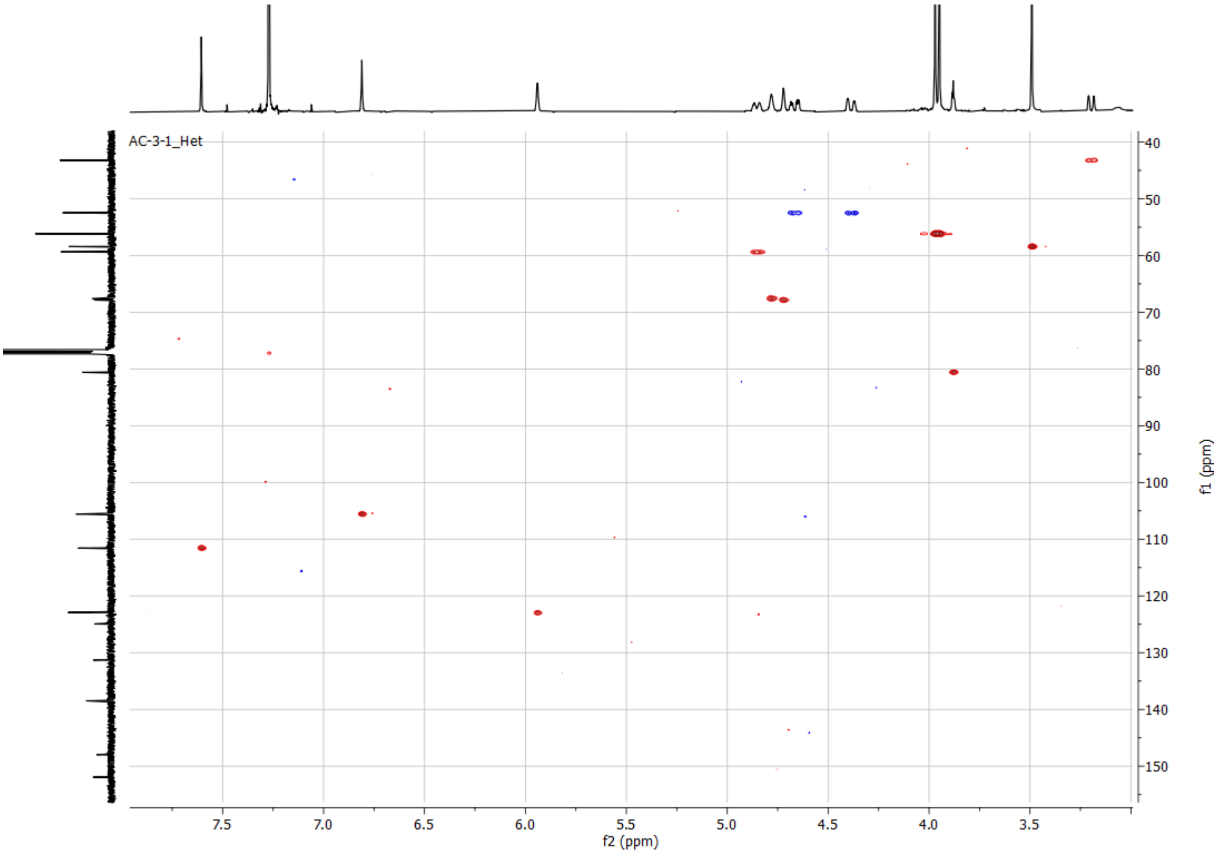

COSY spectrum of 6-oxonarcissidine (2) in $CDCl_3$

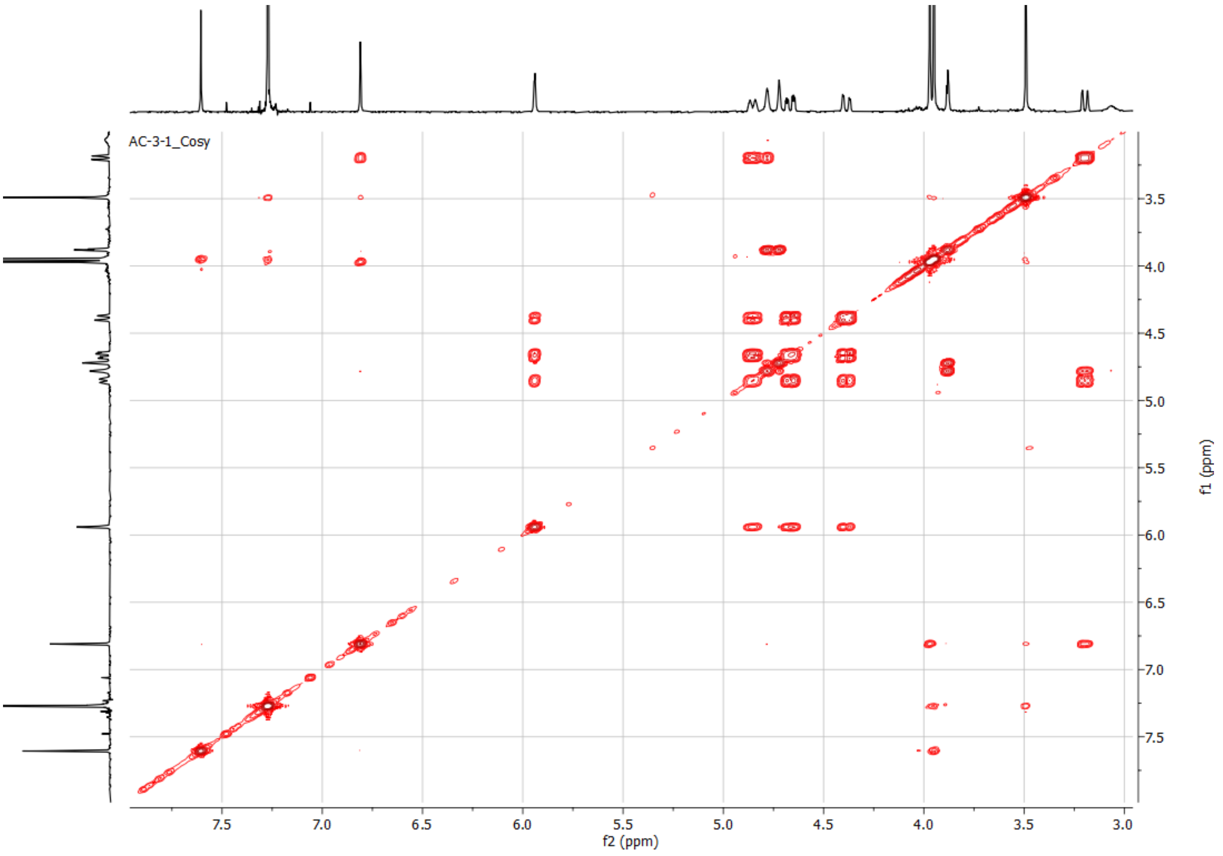

H2BC spectrum of 6-oxonarcissidine (2) in $CDCl_3$

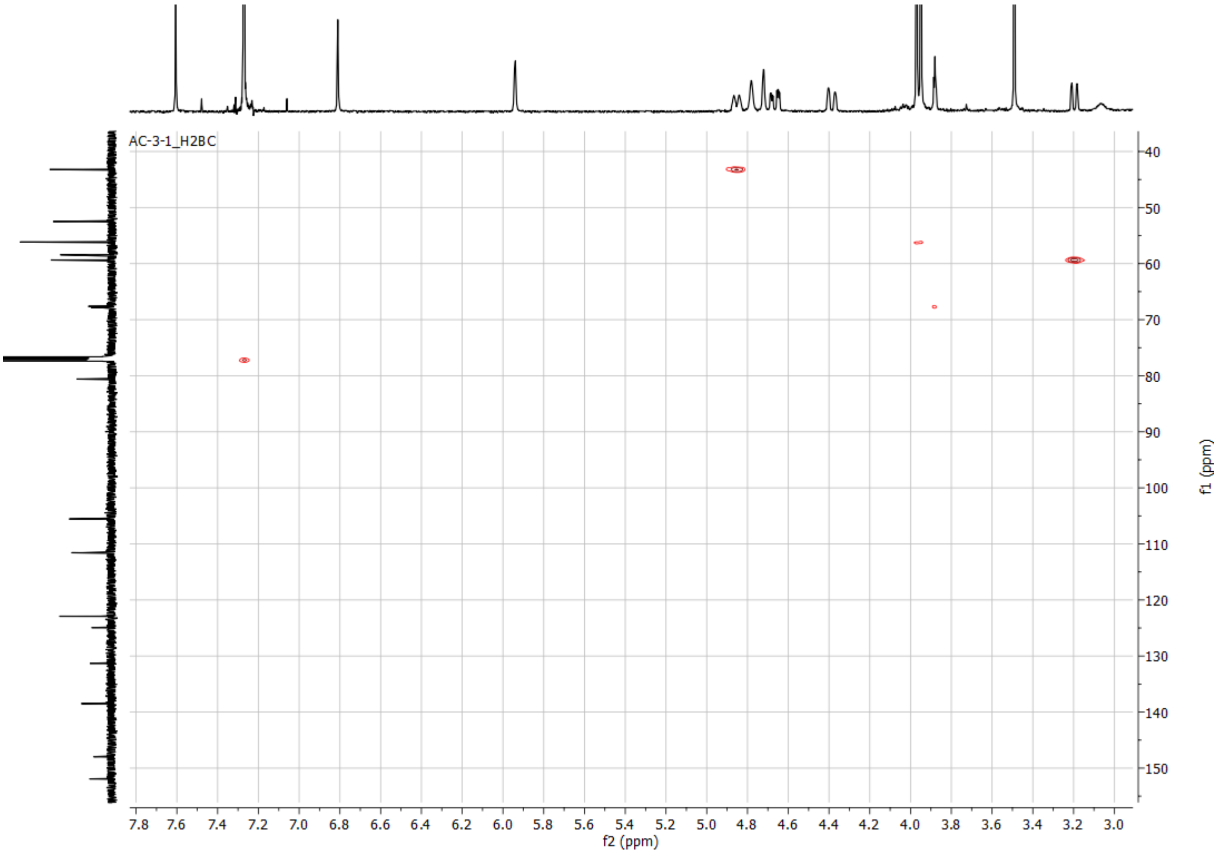

HMBC spectrum of 6-oxonarcissidine (2) in $CDCl_3$

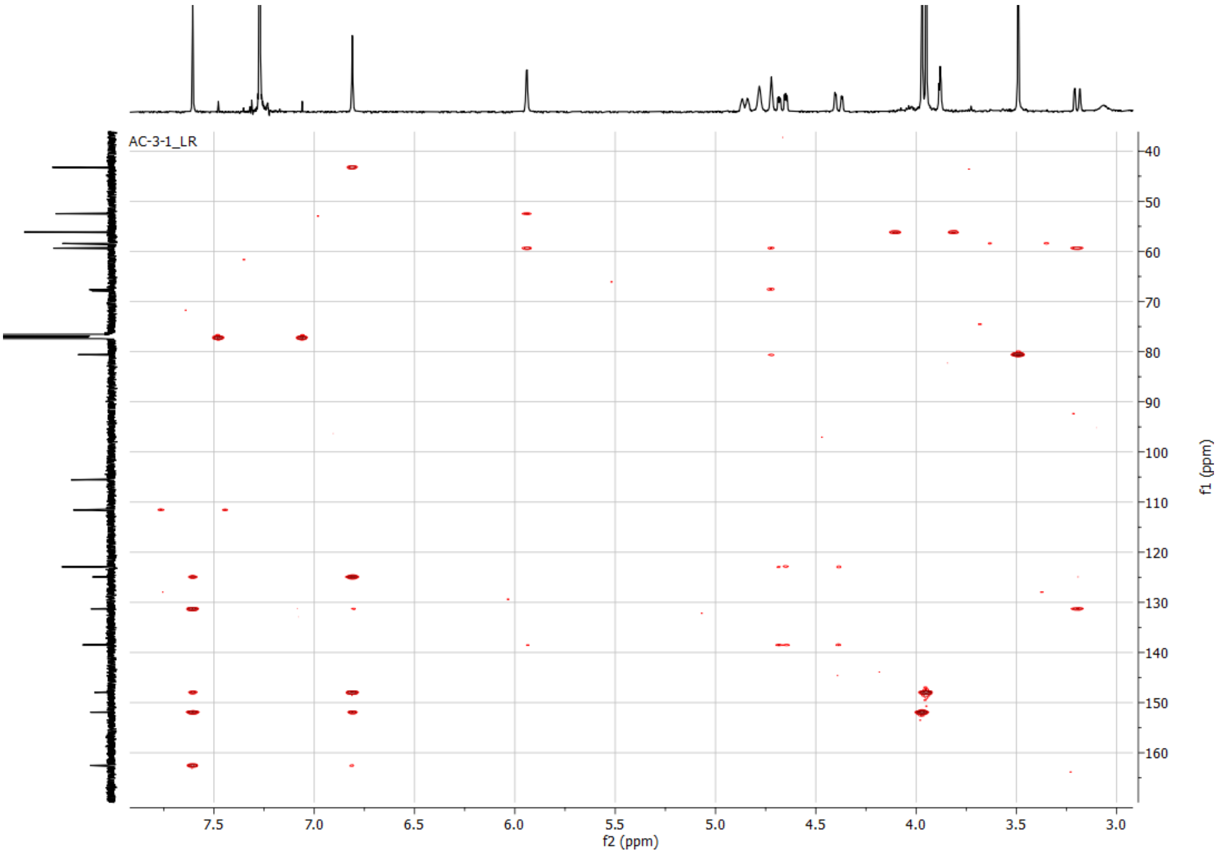

NOESY spectrum of 6-oxonarcissidine (2) in $CDCl_3$

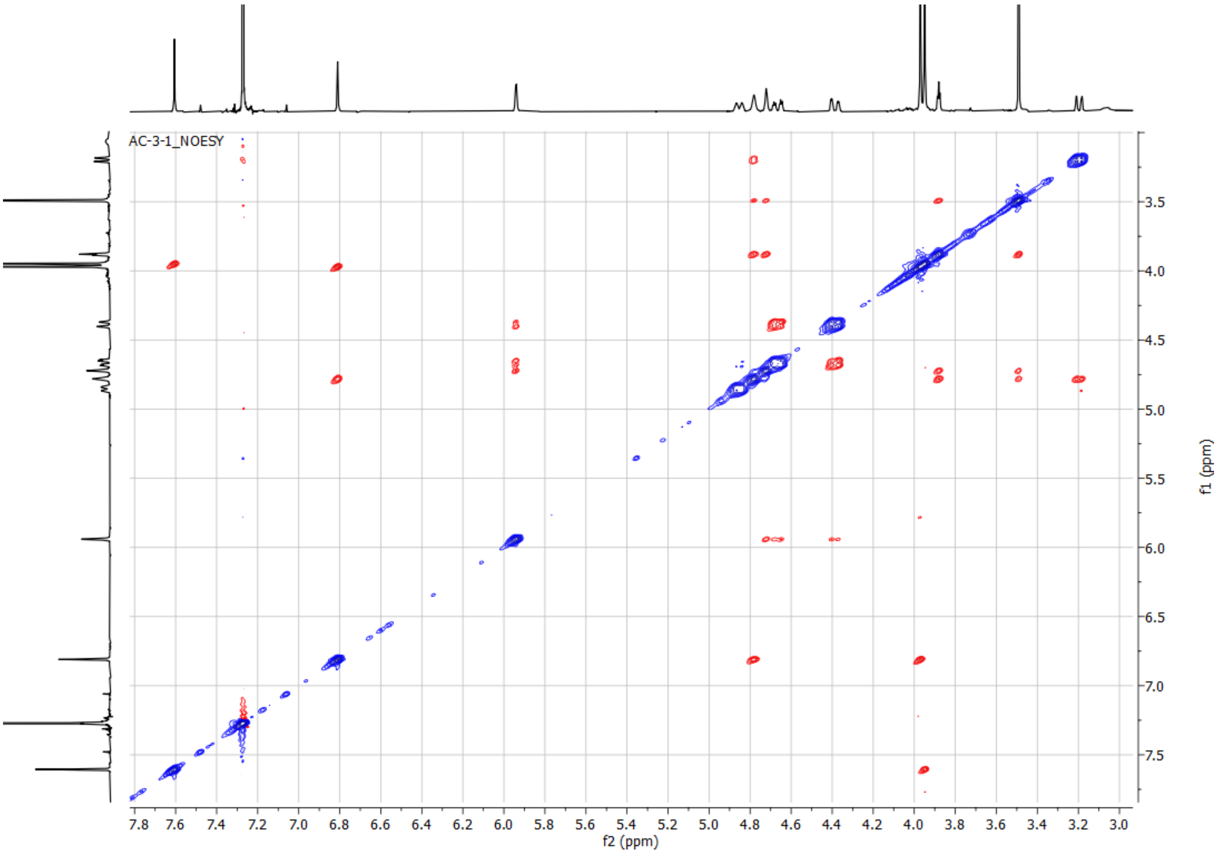

UV spectrum of 6-oxonarcissidine (2) in MeOH

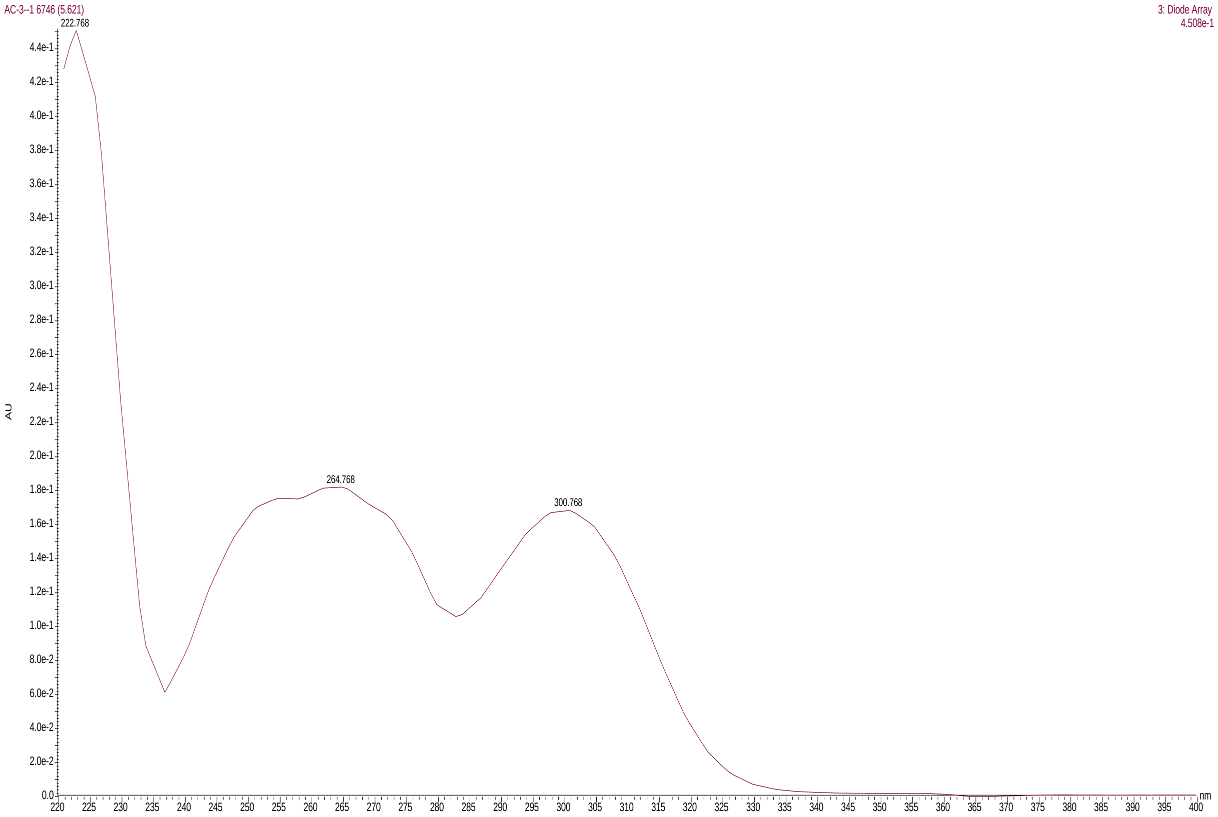

Table S2. Comparison of ^{1}H and ^{13}C NMR chemical shifts ($CDCl_3$) and optical rotation of 6-oxonarcissidine (**2**), 1-*O*-acetyl-3-*O*-methyl-6-oxonarcissidine (**2a**), and narcissidine (**6**)

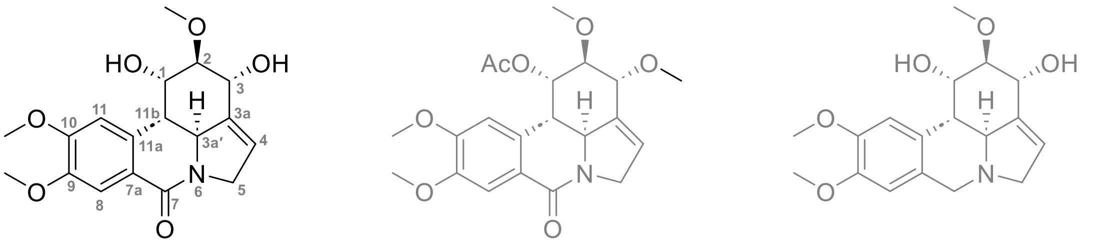

2 **2a** **6**

No.		**2**		**2a**[2]		**6**
	δ_C	δ_H, mult (*J* in Hz)	δ_C	δ_H, mult (*J* in Hz)	δ_C	δ_H, mult (*J* in Hz)
1	67.6	4.78, bs	66.8	5.78, br t (2.7)	68.1	4.70–4.65, m
2	80.6	3.88, t (2.1)	79.1	3.77, dd (2.9, 2.0)	80.6	3.80, dd (3.2, 2.4)
3	67.9	4.72, bs	75.8	4.06, br d (1.9)	69.2	4.70–4.65, m
3a	138.5		136.1		140.8	
3a′	59.4	4.85, d (13.4)	60.1	4.72, m	62.5	3.86–3.82, m, overlap
4	122.9	5.95–5.92, m	125.4	5.97, q (1.8)	121.4	5.59–5.57, m
5	52.5	4.67, ddd (16.2, 4.4, 2.1)<br>4.41–4.36, m	52.6	4.68, ddd (16.1, 5.1, 2.0)<br>4.40, ddd (16.0, 3.2, 1.6)	62.9	4.07–4.04, m, overlap<br>3.58, ddd (14.5, 5.7, 2.0), overlap
7	162.5		162.6		54.6	4.07, d (13.0)<br>3.56, d (13.0)
7a	124.9		130.8		127.4	
8	111.6	7.61, s	111.4	7.57, s	110.6	6.69, s
9	148.0		148.0		147.1	
10	151.9		151.9		148.4	
11	105.6	6.81, s	105.7	6.58, s	107.9	6.90, s
11a	131.3		124.6		129.7	
11b	43.2	3.22–3.17, m	41.6	3.30, ddd (12.8, 2.5, 0.9)	41.8	2.71, dd (11.1, 1.4)
2-OCH_3	58.4	3.49, s	58.9	3.51, s	58.2	3.45, s
3-OCH_3	-	-	56.5	3.27, s	-	-
9-OCH_3	56.2	3.95, s	56.2	3.93, s	56.1	3.83, s, overlap
10-OCH_3	56.2	3.97, s	56.2	3.86, s	56.0	3.88, s
OCO**CH_3**	-	-	21.1	2.01	-	-
O**C**OCH_3	-	-	171.1		-	-
$[\alpha]_D$		–84 ° (*c* 0.10, MeOH, 23 °C)		–123 ° (*c* 0.26, $CHCl_3$, 22 °C)		–56 ° (*c* 0.10, MeOH, 23 °C)

HRESIMS spectrum of 6-*O*-ethylzephyranine F (3)

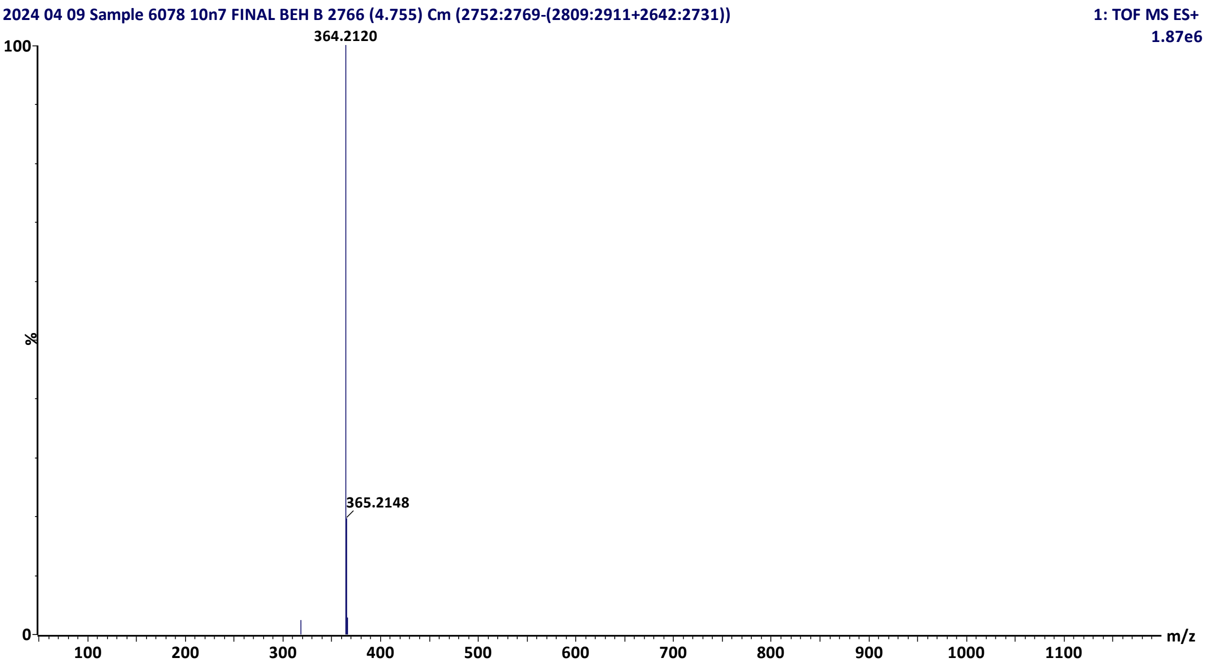

^{1}H NMR spectrum (500 MHz) of 6-*O*-ethylzephyranine F (3) in $CDCl_3$

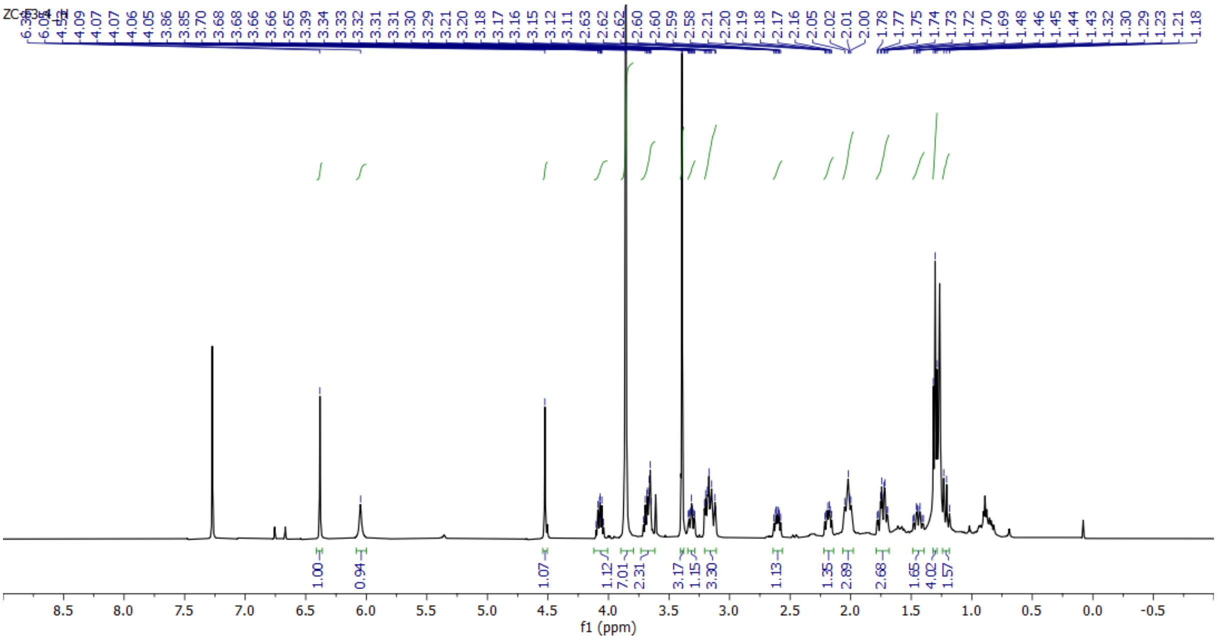

^{13}C NMR spectrum (125.7 MHz) of 6-*O*-ethylzephyranine F (3) in $CDCl_3$

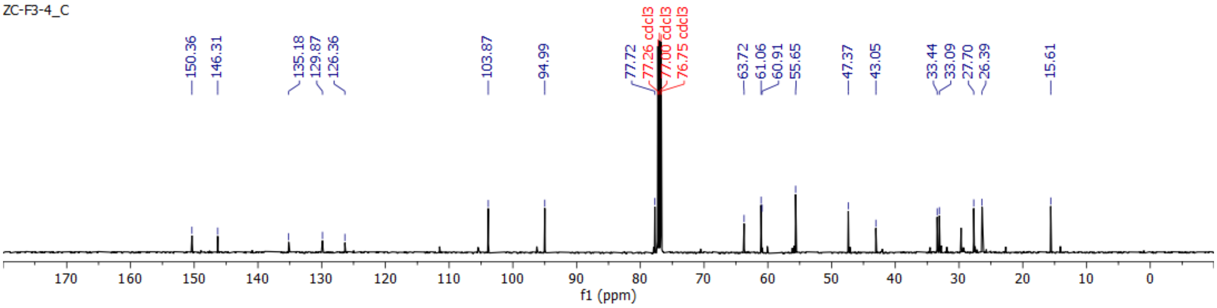

HSQC spectrum of 6-*O*-ethylzephyranine F (3) in $CDCl_3$

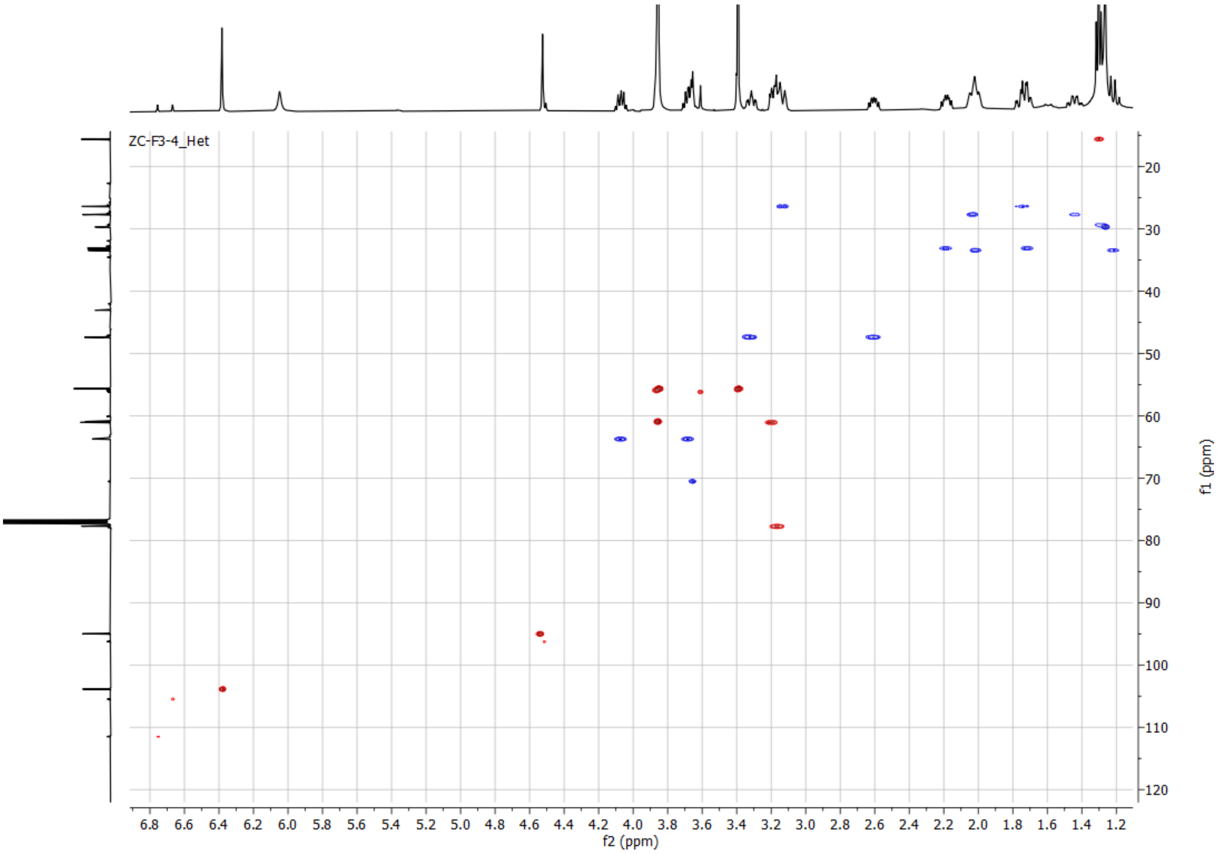

COSY spectrum of 6-*O*-ethylzephyranine F (3) in $CDCl_3$

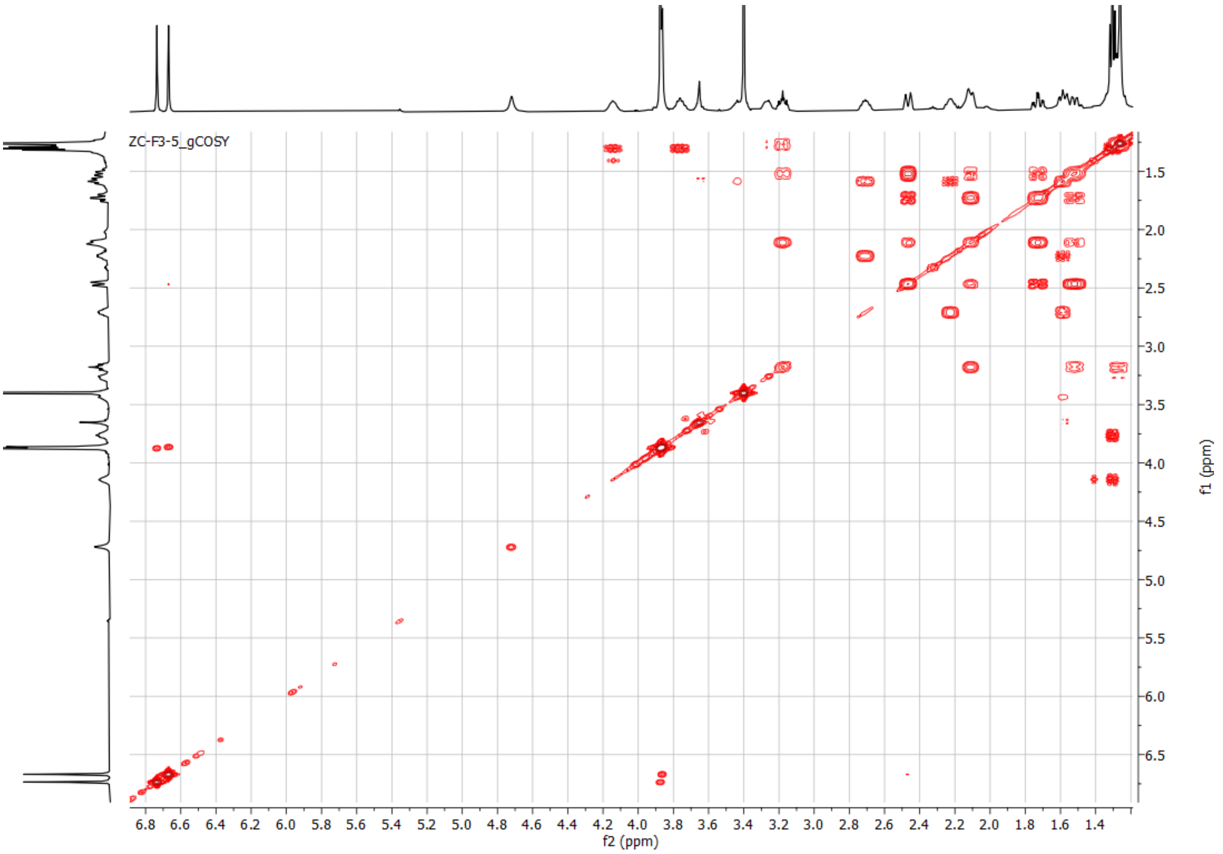

H2BC spectrum of 6-*O*-ethylzephyranine F (3) in $CDCl_3$

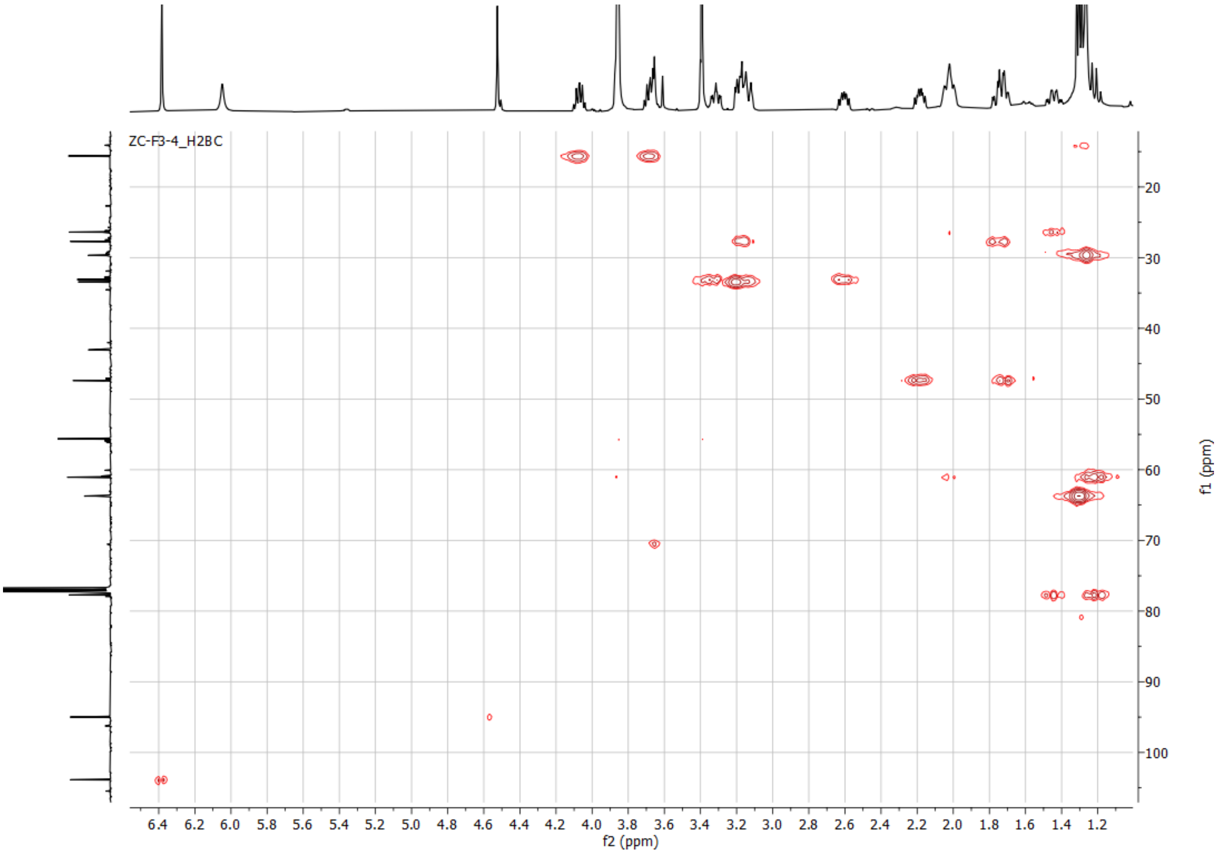

HMBC spectrum of 6-*O*-ethylzephyranine F (3) in $CDCl_3$

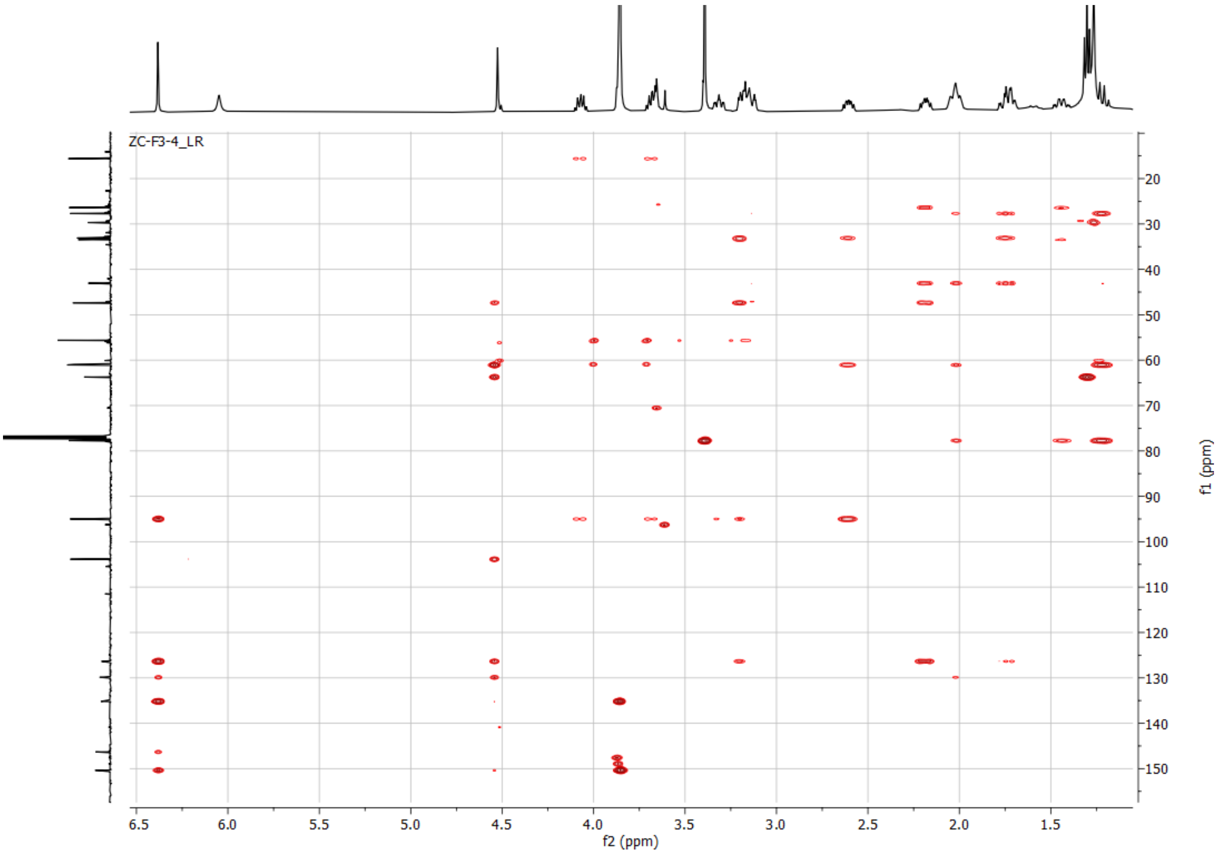

NOESY spectrum of 6-*O*-ethylzephyranine F (3) in $CDCl_3$

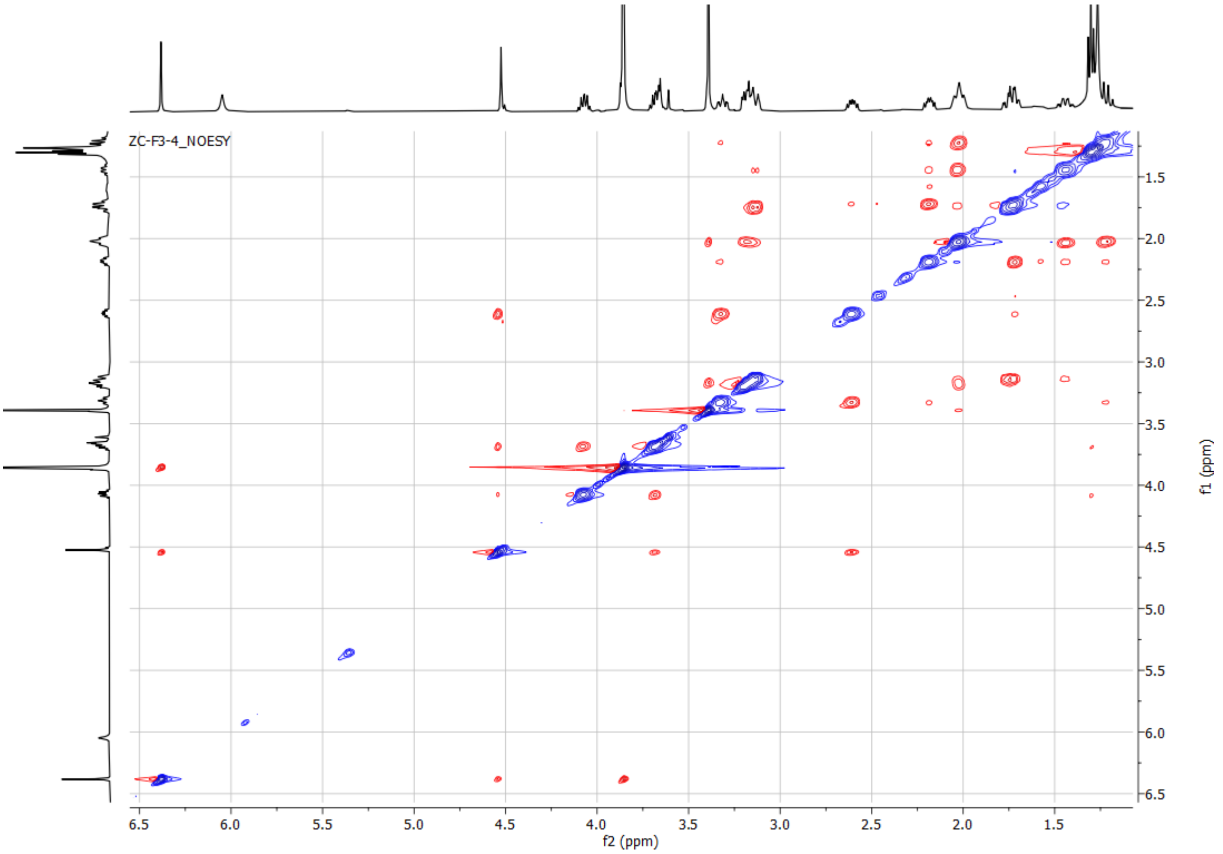

UV spectrum of 6-*O*-ethylzephyranine F (3) in MeOH

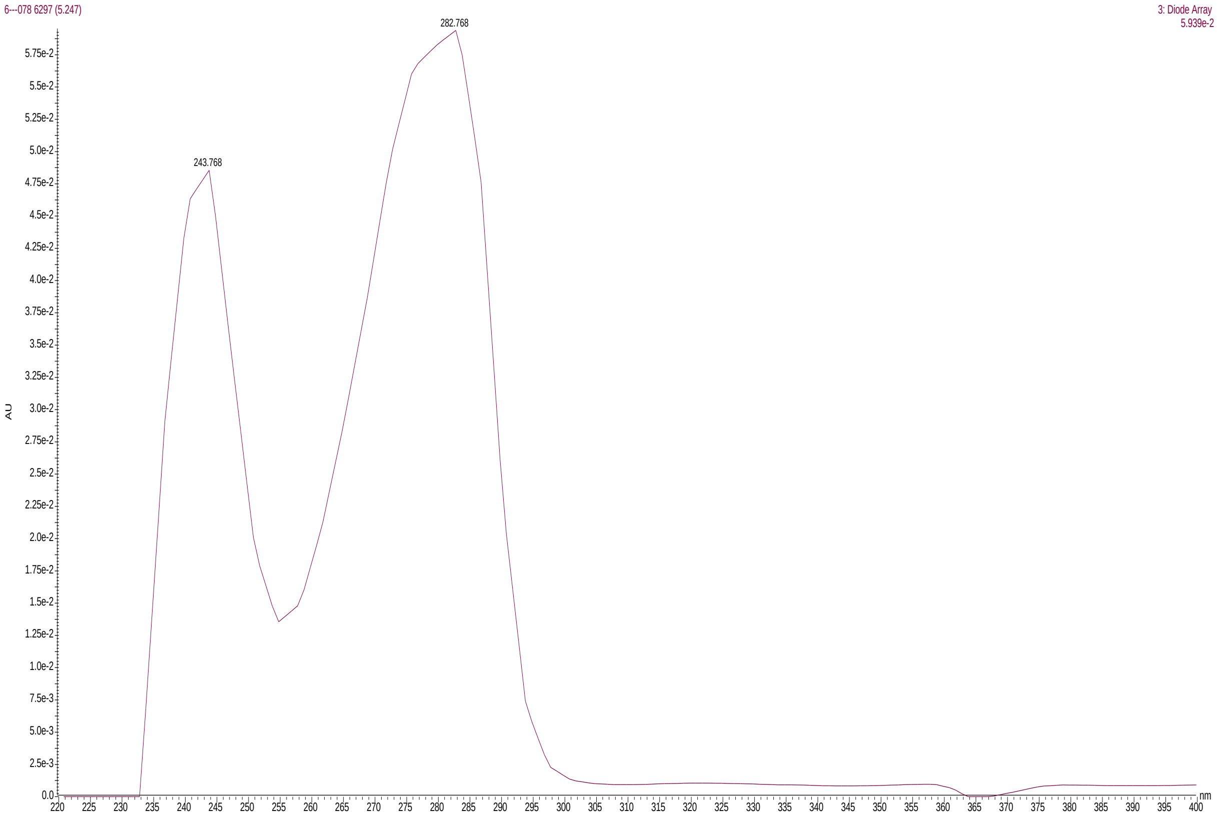

HRESIMS spectrum of 6-*O*-ethylzephyranine E (4)

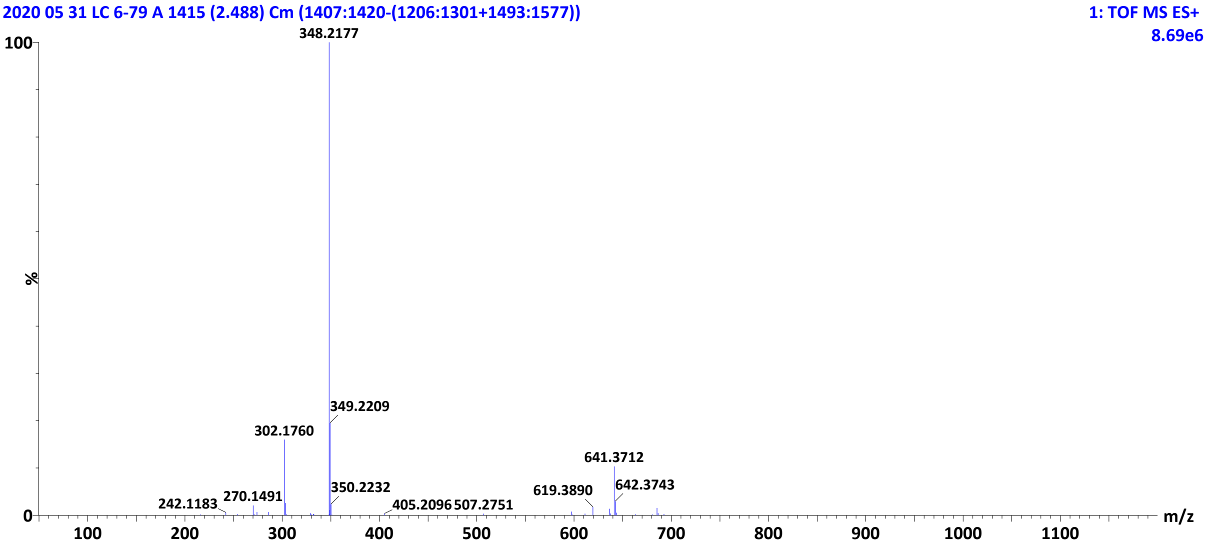

^{1}H NMR spectrum (500 MHz) of 6-*O*-ethylzephyranine E (4) in $CDCl_3$

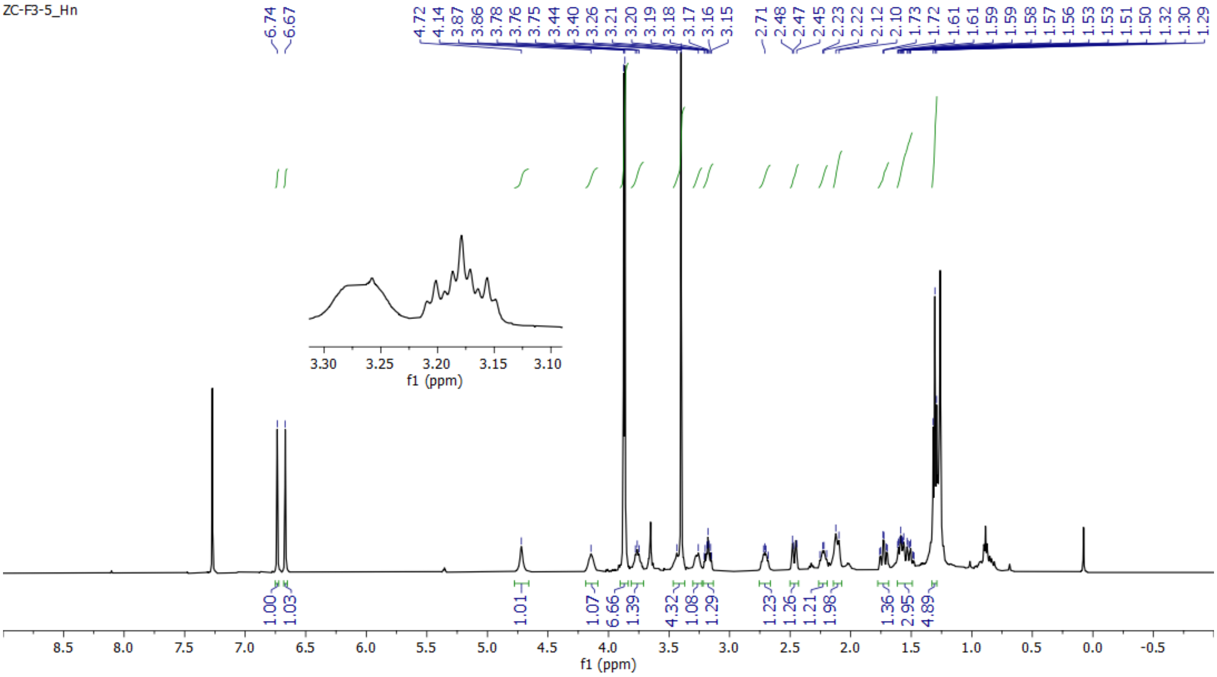

^{13}C NMR spectrum (125.7 MHz) of 6-*O*-ethylzephyranine E (4) in $CDCl_3$

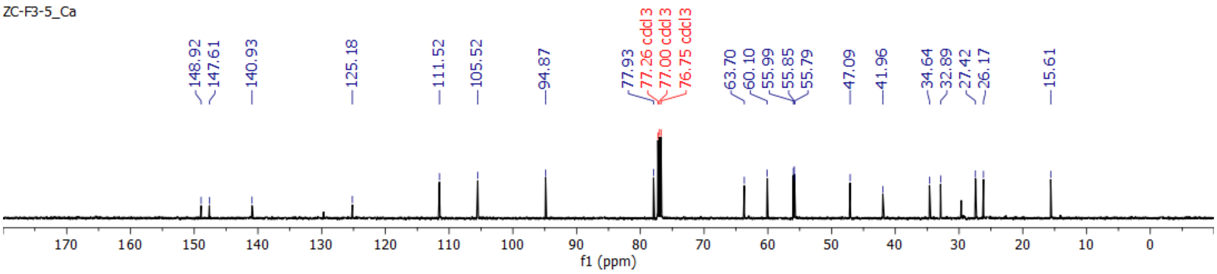

HSQC spectrum of 6-*O*-ethylzephyranine E (4) in $CDCl_3$

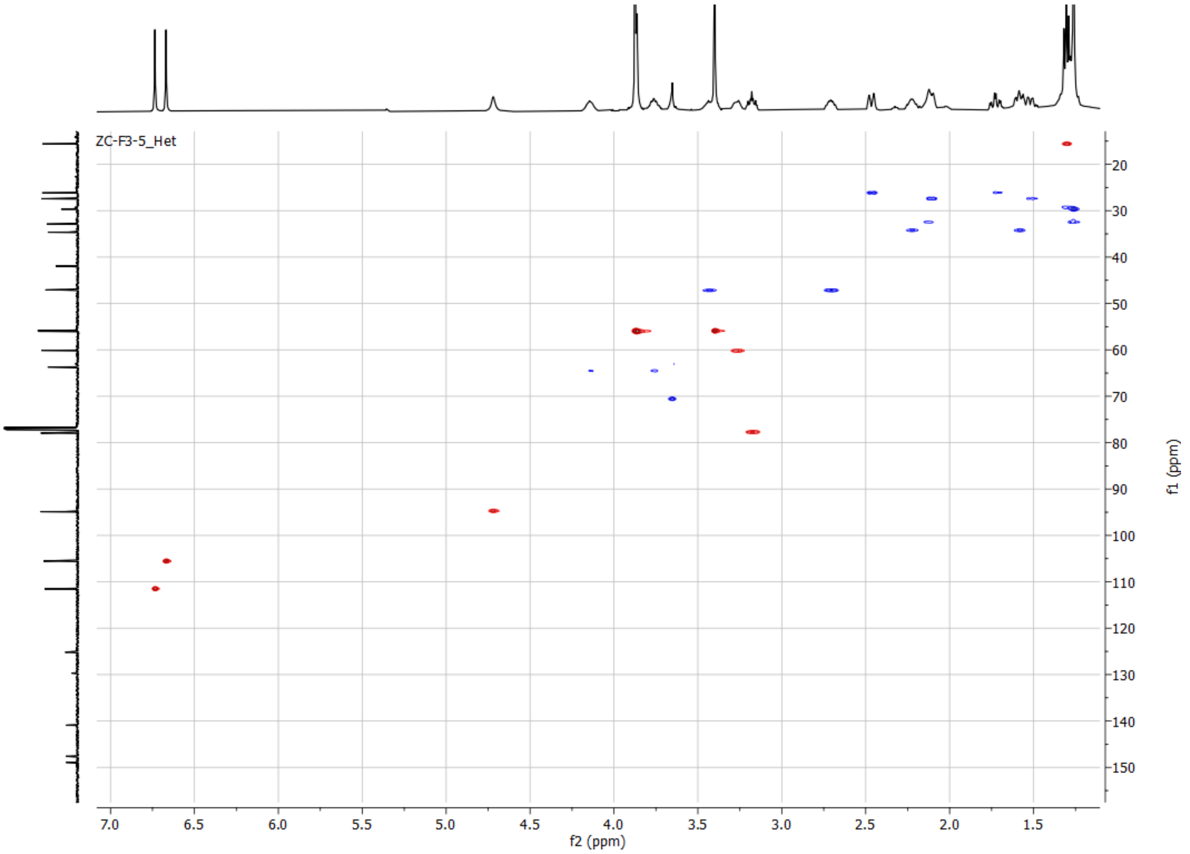

COSY spectrum of 6-*O*-ethylzephyranine E (4) in $CDCl_3$

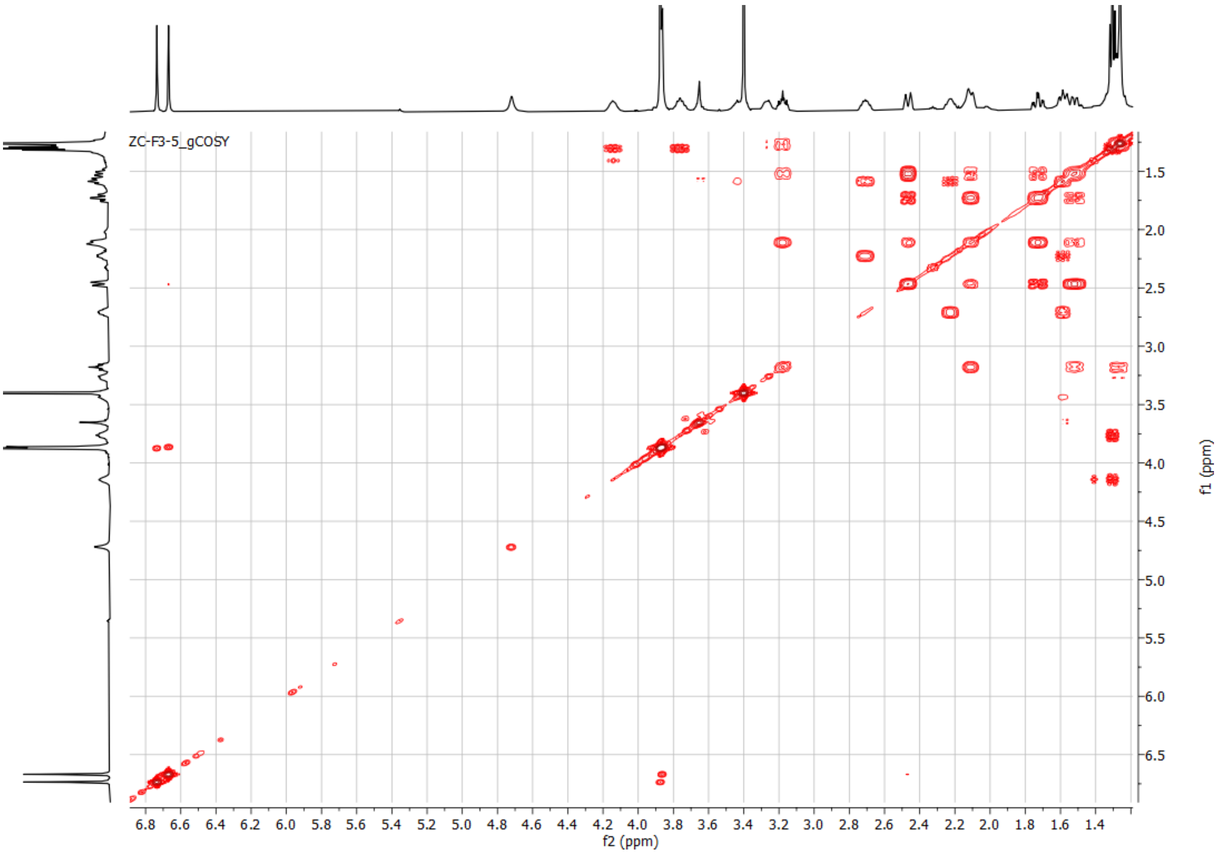

H2BC spectrum of 6-*O*-ethylzephyranine E (4) in $CDCl_3$

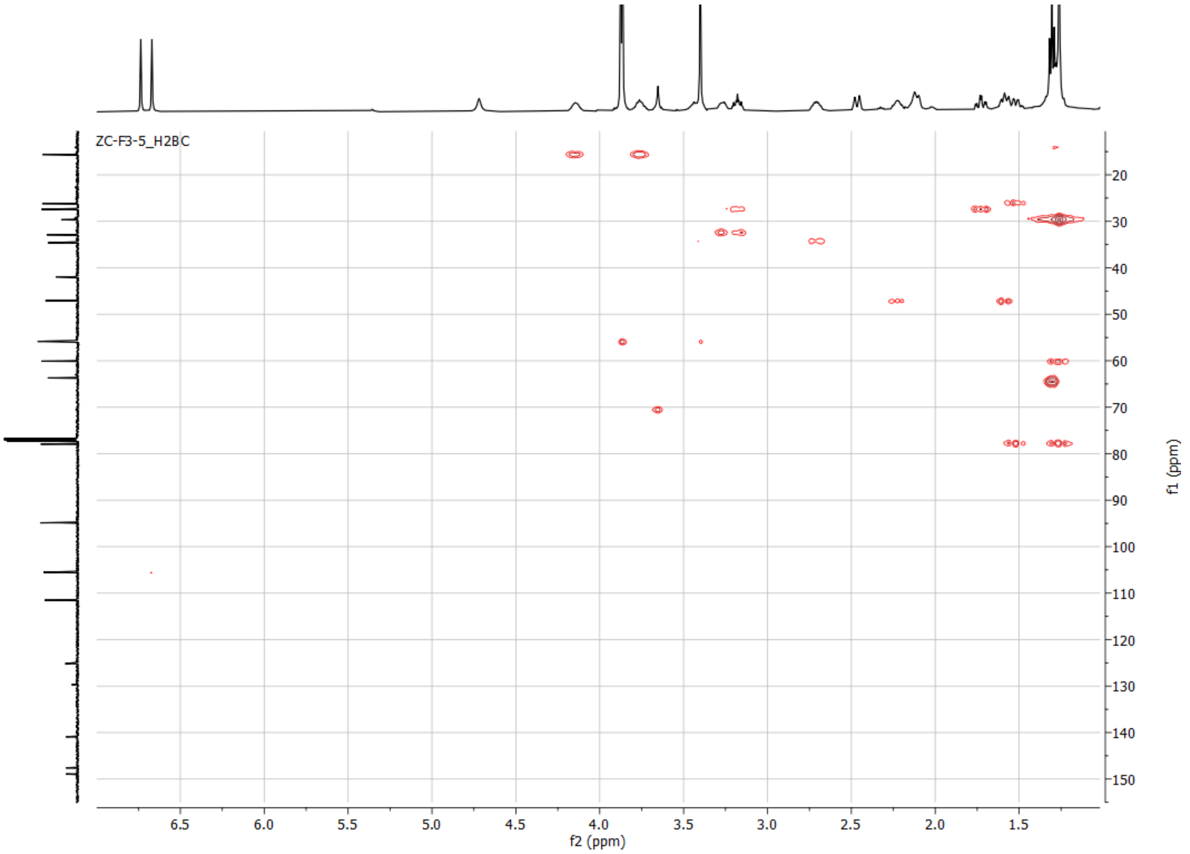

HMBC spectrum of 6-*O*-ethylzephyranine E (4) in $CDCl_3$

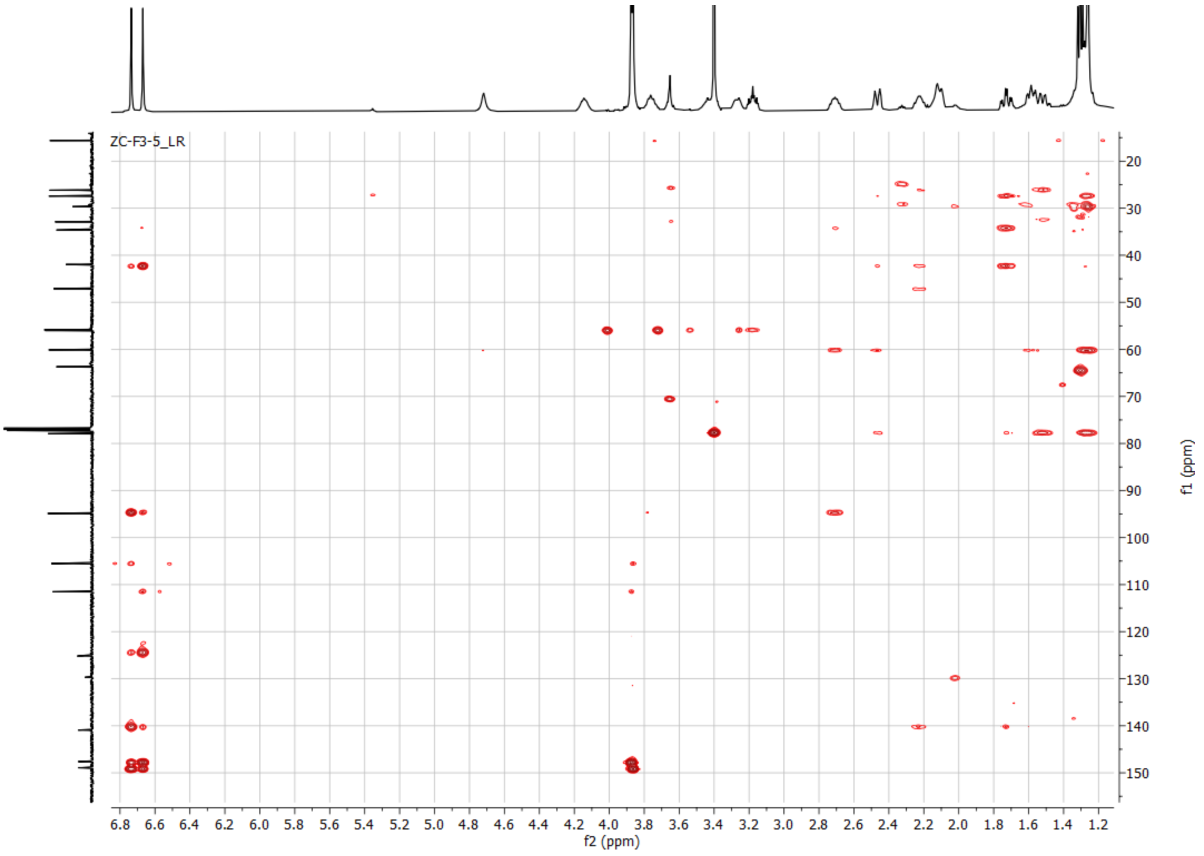

NOESY spectrum of 6-*O*-ethylzephyranine E (4) in $CDCl_3$

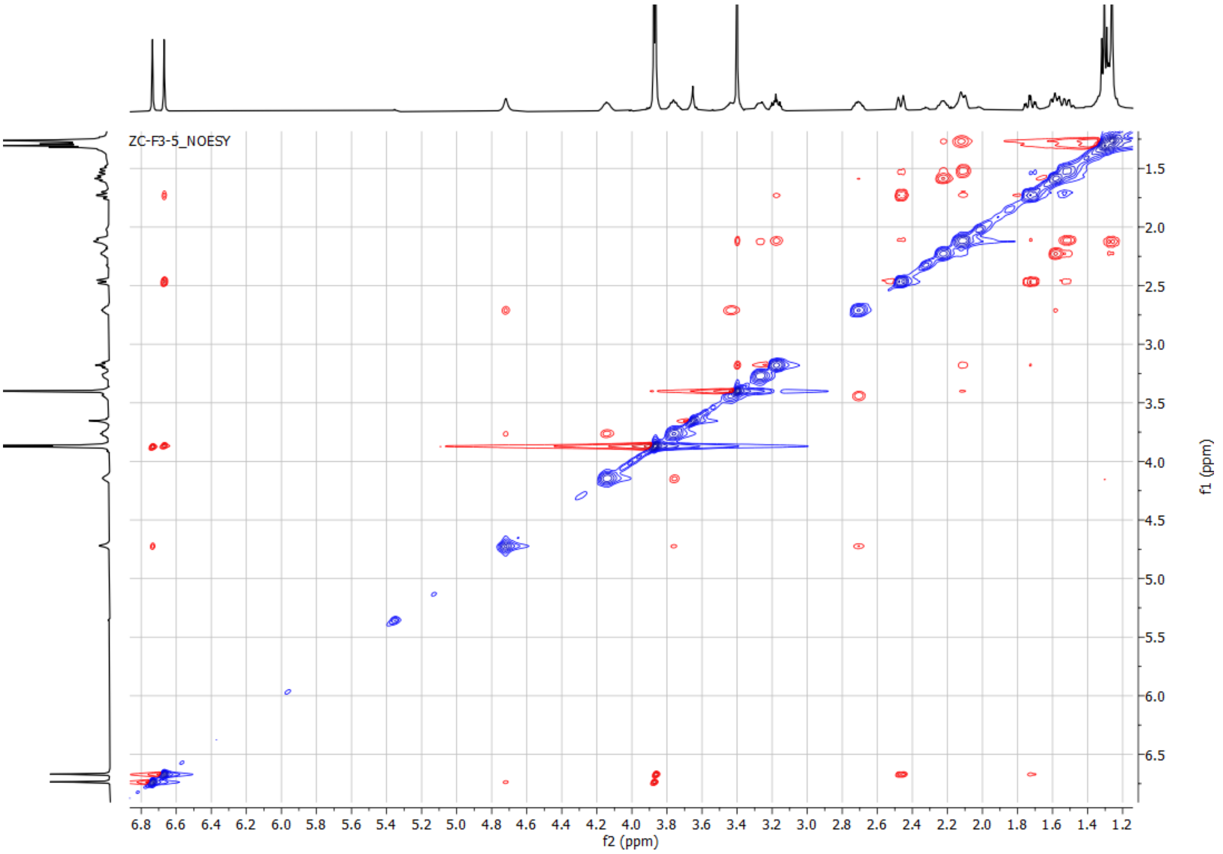

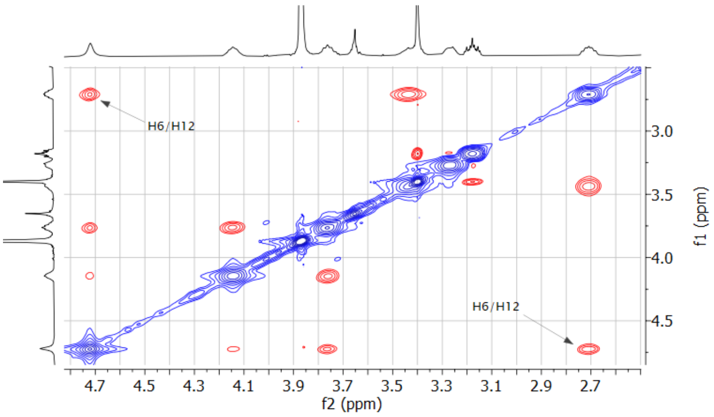

UV spectrum of 6-*O*-ethylzephyranine E (4) in MeOH

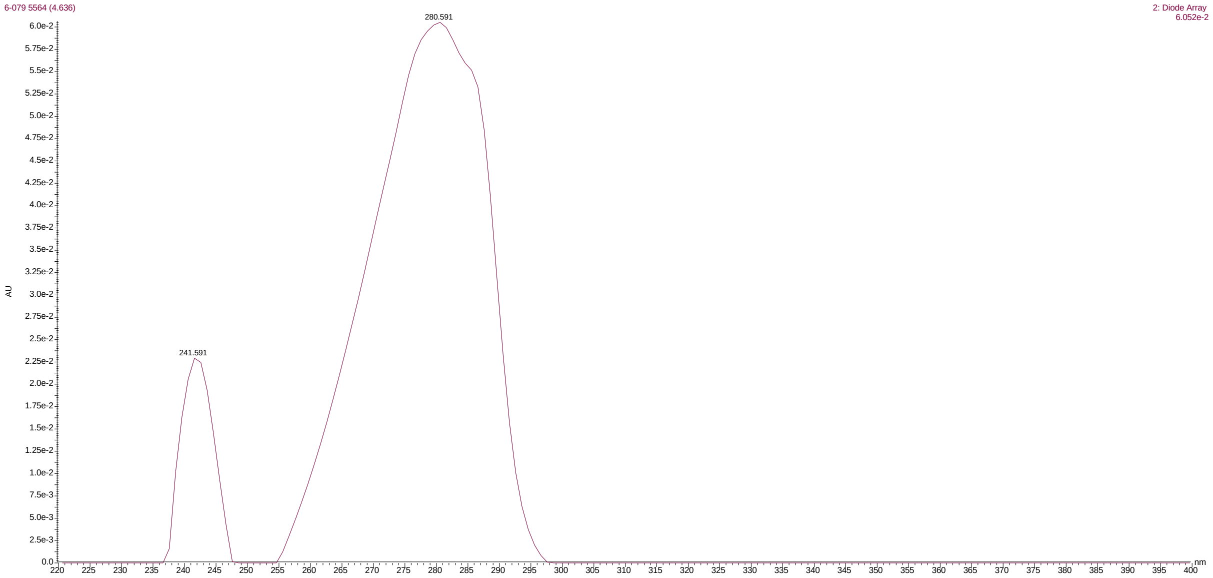

ECD spectra of 6-*O*-ethylzephyranine E (4), zephyranine C (8), zephyranine E (9), and zephyranine F (10) in MeOH

Different spectral ranges (200–400 nm and 220–400 nm) showing the visual difference.

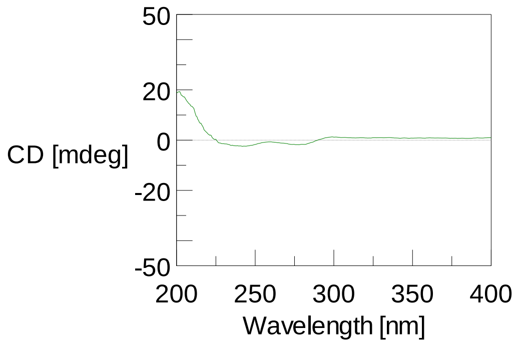

6-*O*-ethylzephyranine E (4) [200–400 nm]

ECD (c 0.1 mg/ml, MeOH)

$\Delta\varepsilon_{202} = +20.29$ $[\theta]_{202} = +66916$

$\Delta\varepsilon_{242} = -2.60$ $[\theta]_{242} = -8574$

$\Delta\varepsilon_{276} = -1.93$ $[\theta]_{276} = -6365$

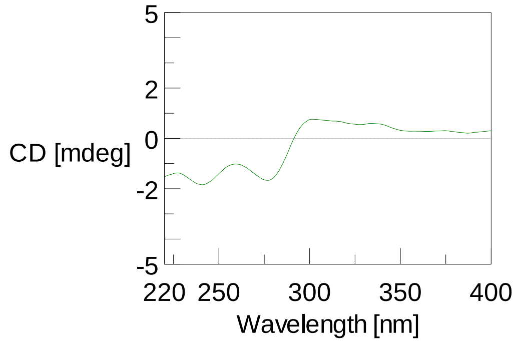

6-*O*-ethylzephyranine E (4) [220–400 nm]

ECD (c 0.1 mg/ml, MeOH)

$\Delta\varepsilon_{241} = -1.94$ $[\theta]_{241} = -6398$

$\Delta\varepsilon_{277} = -1.76$ $[\theta]_{277} = -5804$

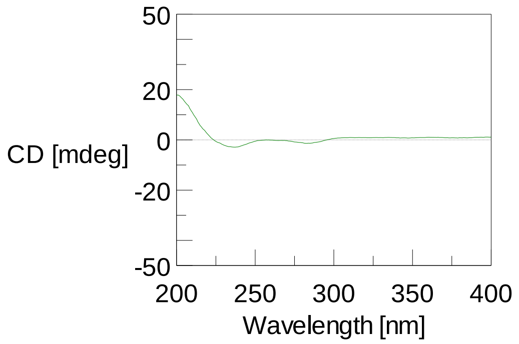

zephyranine E (9) [200–400 nm]

ECD (c 0.1 mg/ml, MeOH)

$\Delta\varepsilon_{200} = +17.29$ $[\theta]_{200} = +57037$

$\Delta\varepsilon_{237} = -2.81$ $[\theta]_{237} = -9256$

$\Delta\varepsilon_{283} = -1.32$ $[\theta]_{283} = -4340$

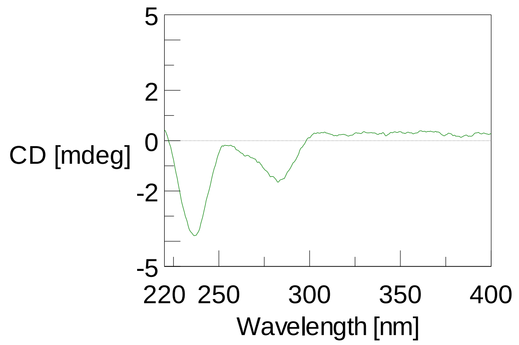

zephyranine E (9) [220–400 nm]

ECD (c 0.1 mg/ml, MeOH)

$\Delta\varepsilon_{237} = -3.64$ $[\theta]_{237} = -12001$

$\Delta\varepsilon_{283} = -1.58$ $[\theta]_{283} = -5202$

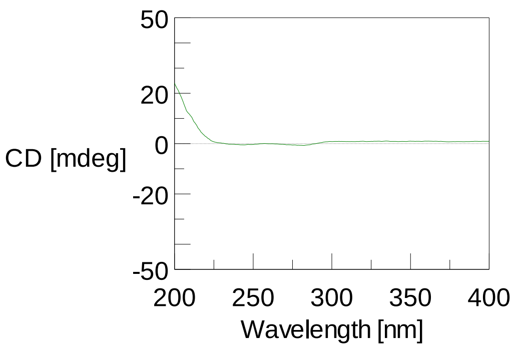

zephyranine F (10) [200–400 nm]

ECD (c 0.1 mg/ml, MeOH)

$\Delta\varepsilon_{200} = +24.37$ $[\theta]_{200} = +80373$

$\Delta\varepsilon_{244} = -0.53$ $[\theta]_{244} = -1743$

$\Delta\varepsilon_{281} = -0.73$ $[\theta]_{281} = -2413$

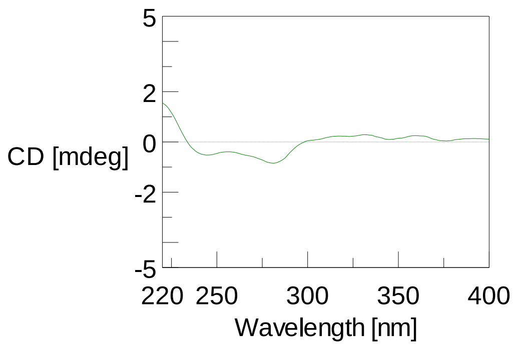

zephyranine F (10) [220–400 nm]

ECD (c 0.1 mg/ml, MeOH)

$\Delta\varepsilon_{244} = -0.83$ $[\theta]_{244} = -2748$

$\Delta\varepsilon_{281} = -0.85$ $[\theta]_{281} = -2815$

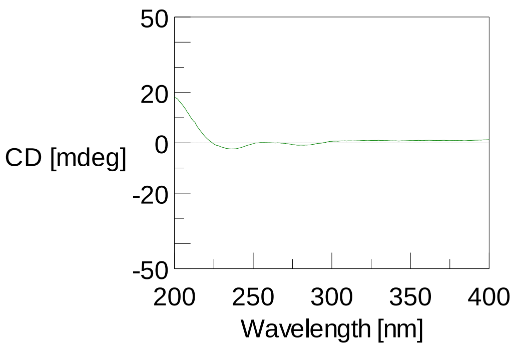

zephyranine C (8) [200–400 nm]

ECD (c 0.1 mg/ml, MeOH)

$\Delta\varepsilon_{200} = +16.91$ $[\theta]_{200} = +55752$

$\Delta\varepsilon_{236} = -2.25$ $[\theta]_{236} = -7415$

$\Delta\varepsilon_{279} = -0.87$ $[\theta]_{279} = -2868$

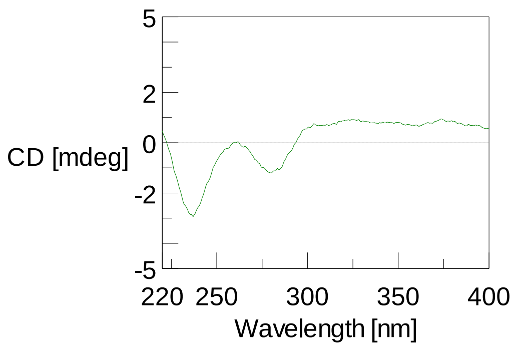

zephyranine C (8) [220–400 nm]

ECD (c 0.1 mg/ml, MeOH)

$\Delta\varepsilon_{237} = -2.72$ $[\theta]_{237} = -8911$

$\Delta\varepsilon_{280} = -1.12$ $[\theta]_{280} = -3692$

HRESIMS spectrum of eugenine (5)

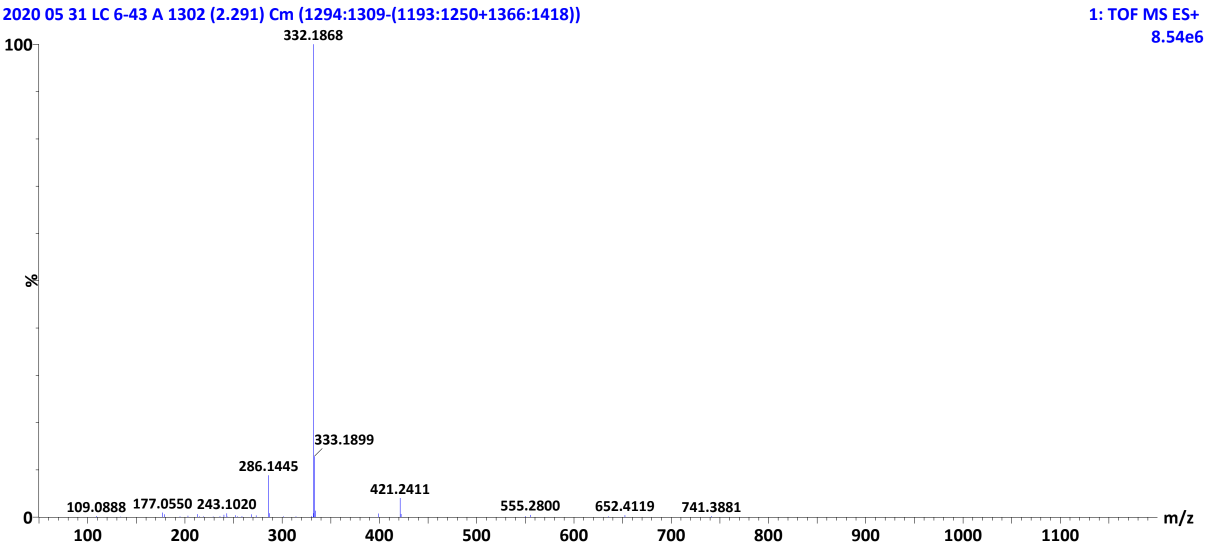

^{1}H NMR spectrum (500 MHz) of eugenine (5) in CD_3OD

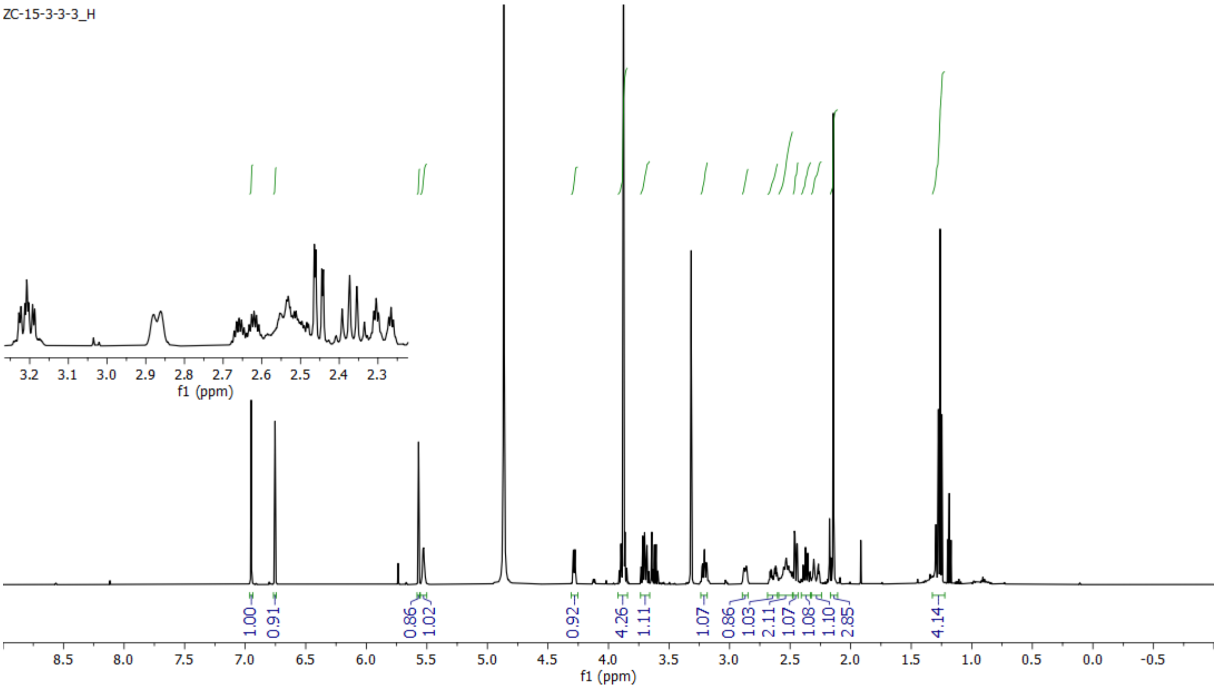

^{13}C NMR spectrum (125.7 MHz) of eugenine (5) in CD_3OD

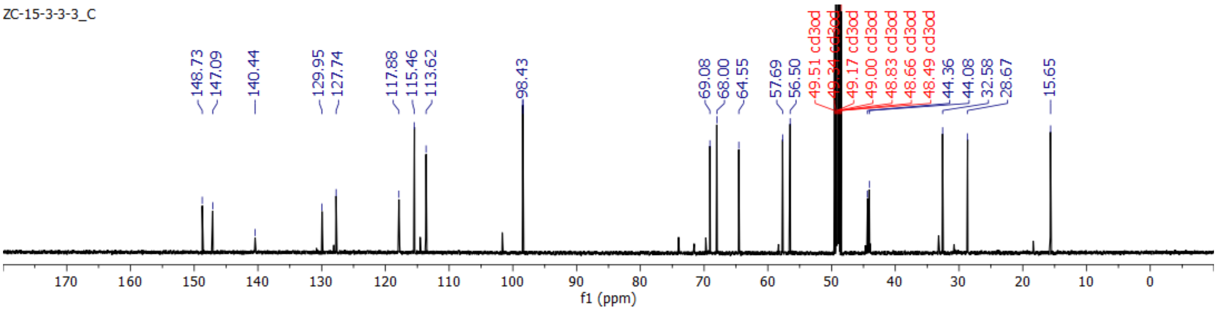

HSQC spectrum of eugenine (5) in CD_3OD

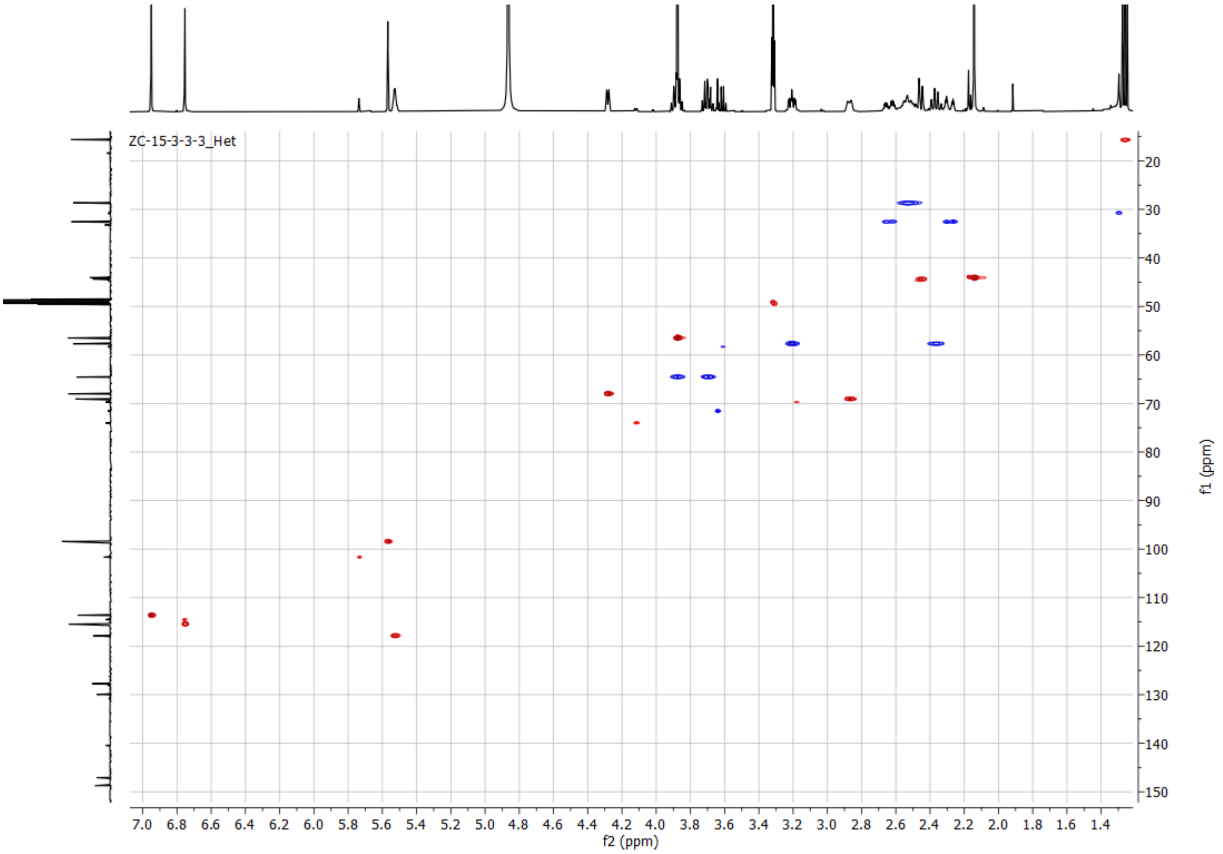

COSY spectrum of eugenine (5) in CD_3OD

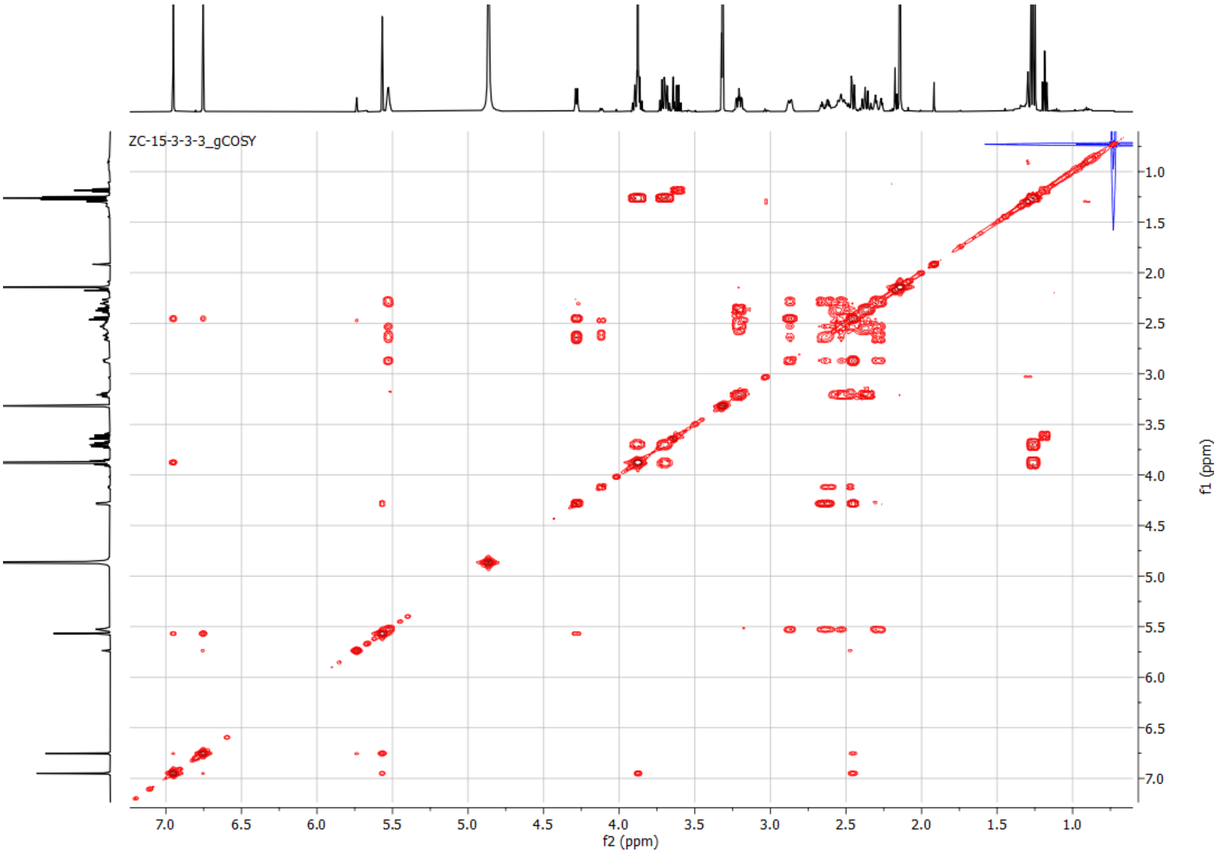

H2BC spectrum of eugenine (5) in CD_3OD

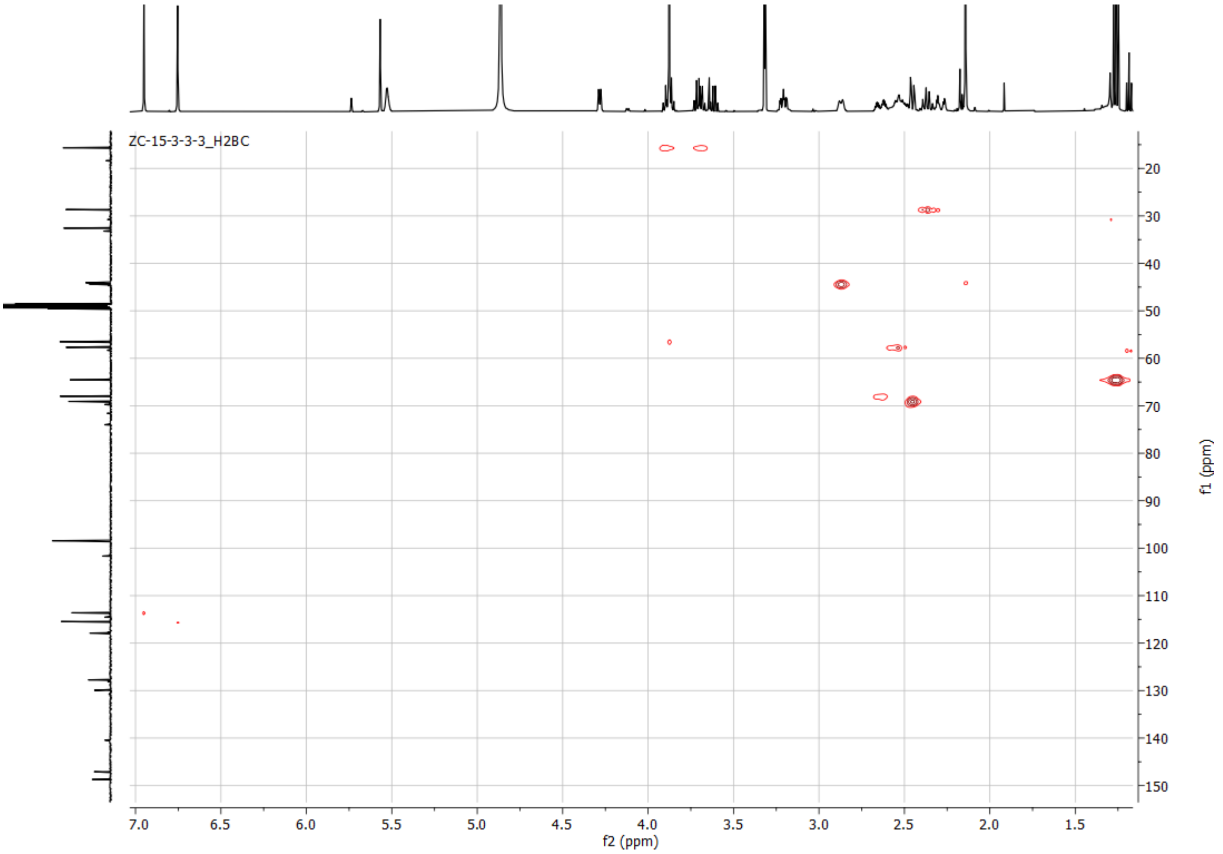

HMBC spectrum of eugenine (5) in CD_3OD

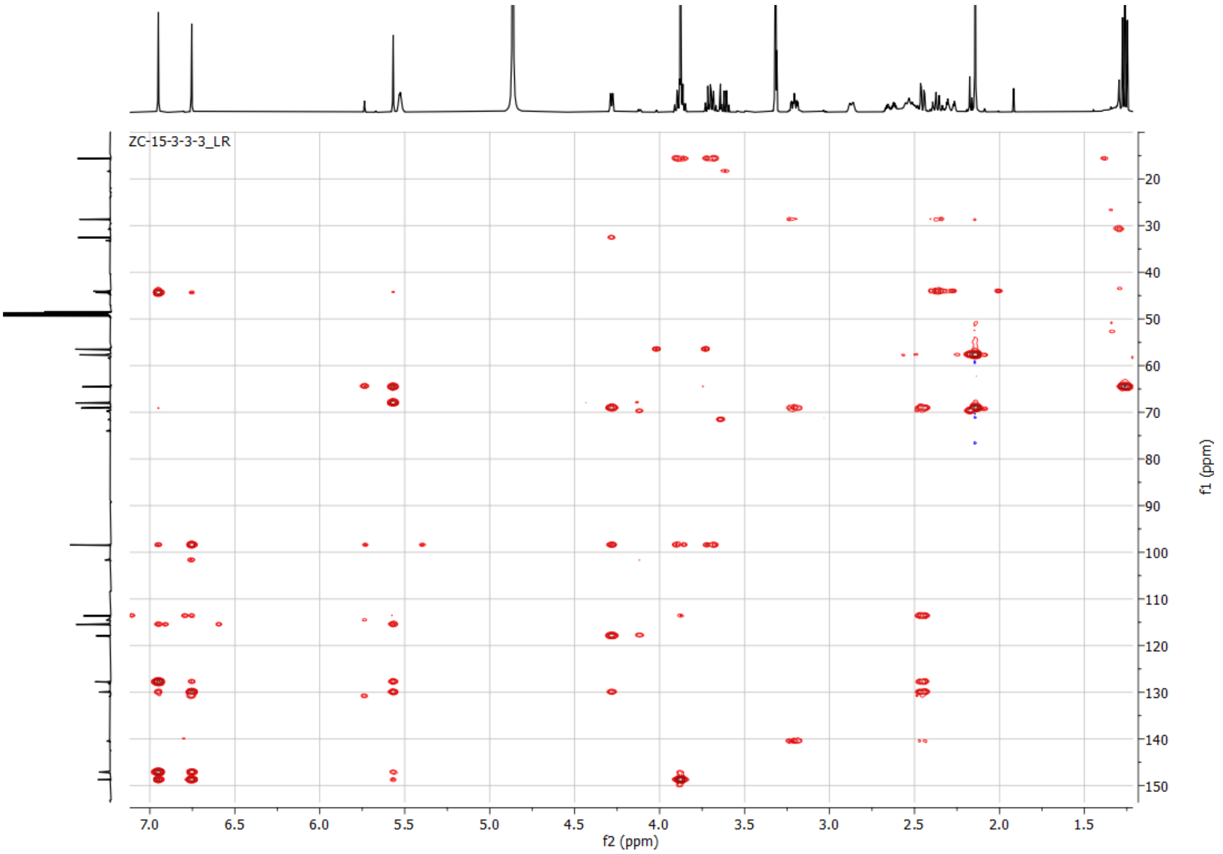

NOESY spectrum of eugenine (5) in CD_3OD

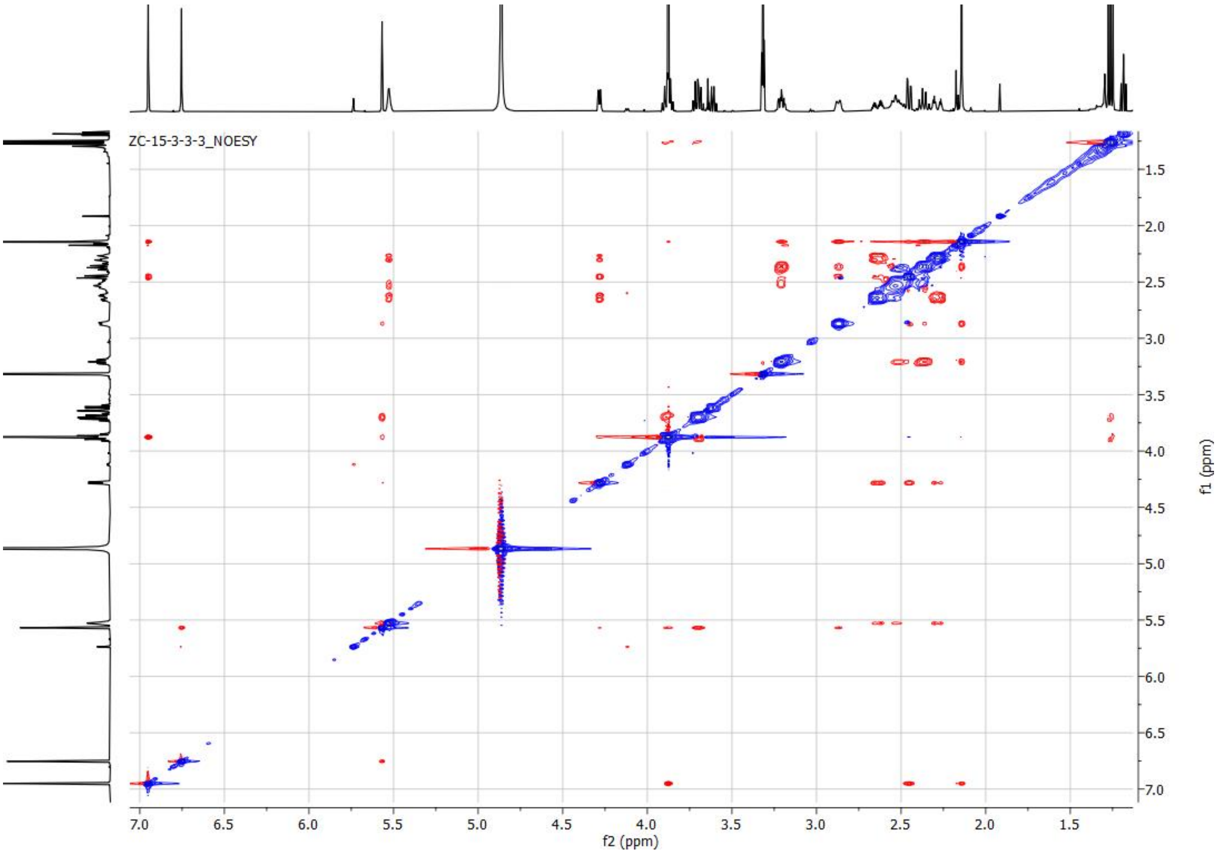

Experiment with eugenine (5) and Pirkel´s reagent in $CDCl_3$

(lower ^{1}H NMR spectrum after reagent addition)

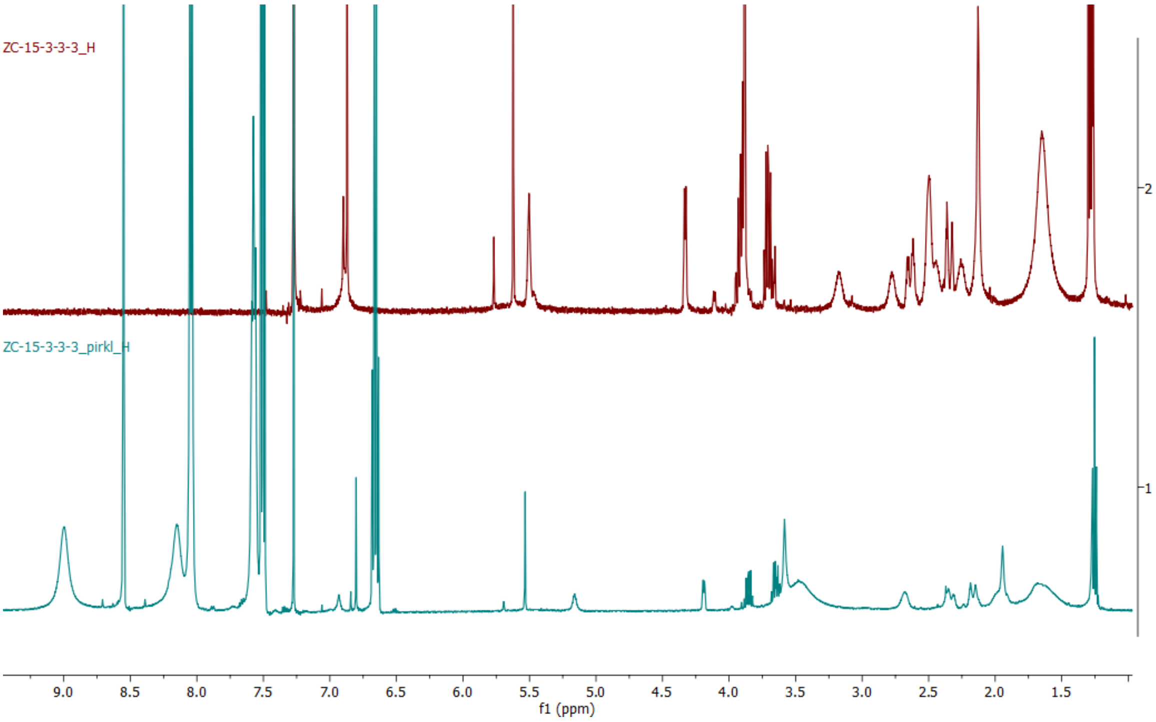

UV spectrum of eugenine (5) in MeOH

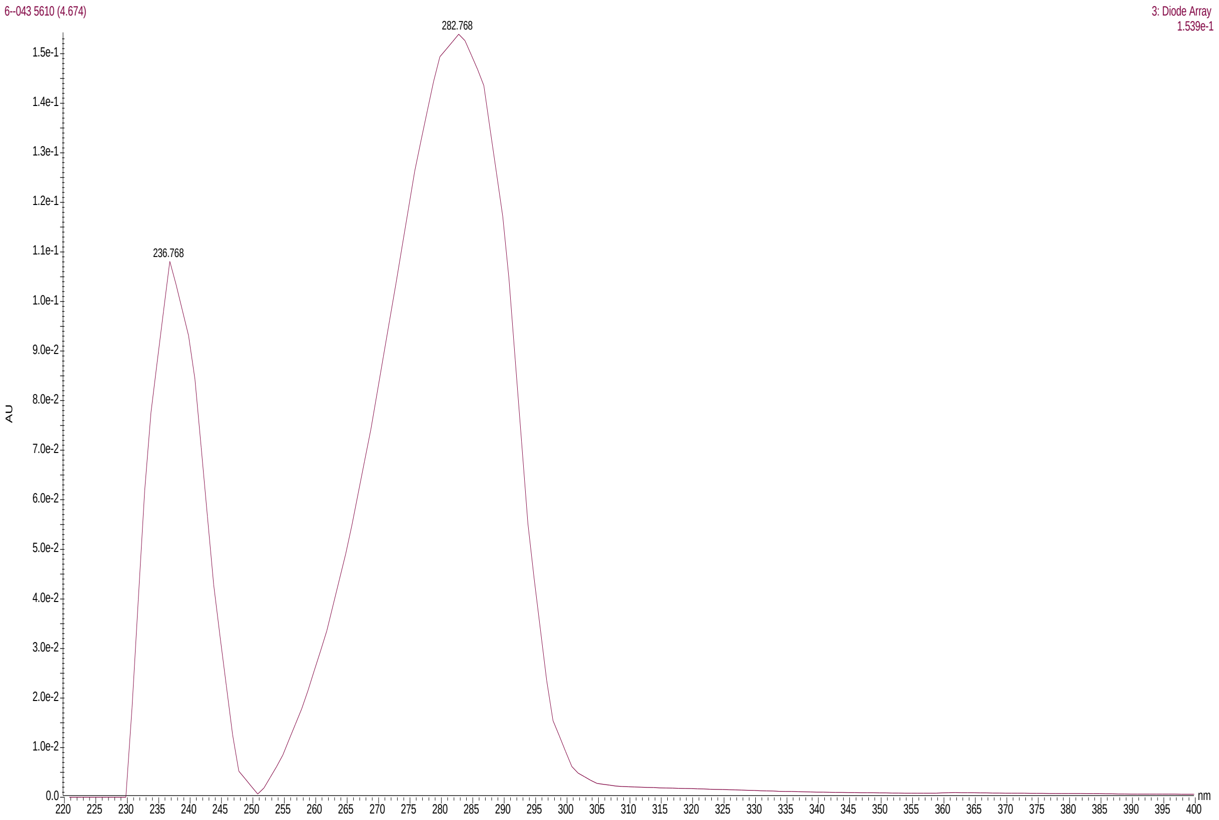

HRESIMS spectrum of narcissidine (6)

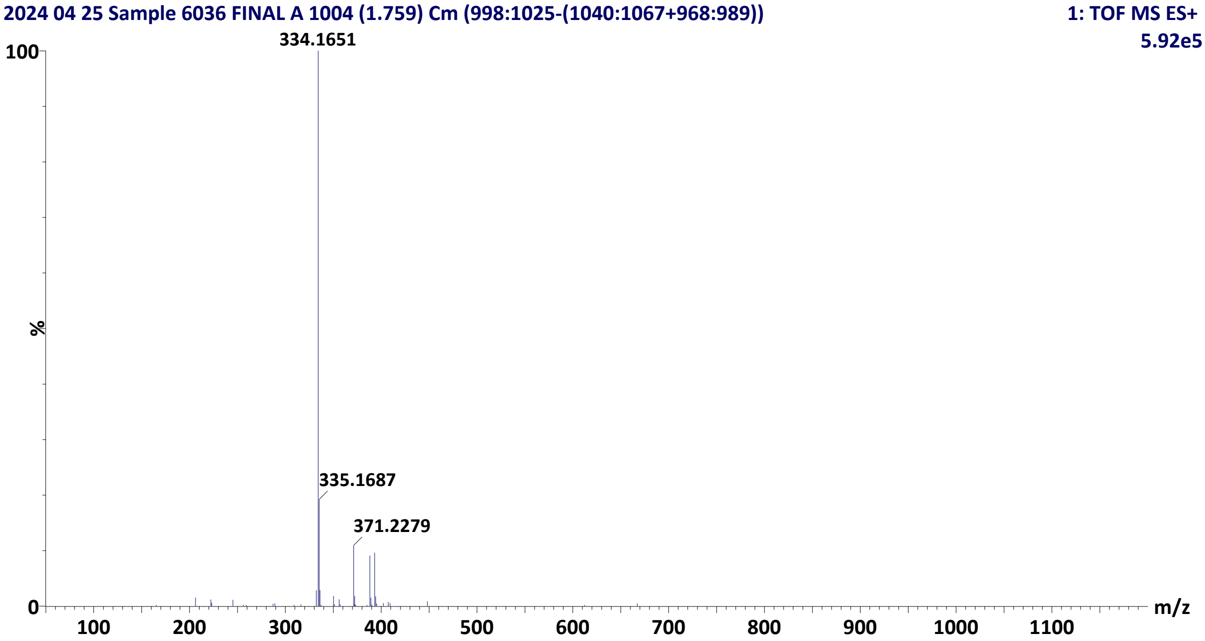

^{1}H NMR spectrum (500 MHz) of narcissidine (6) in $CDCl_3$

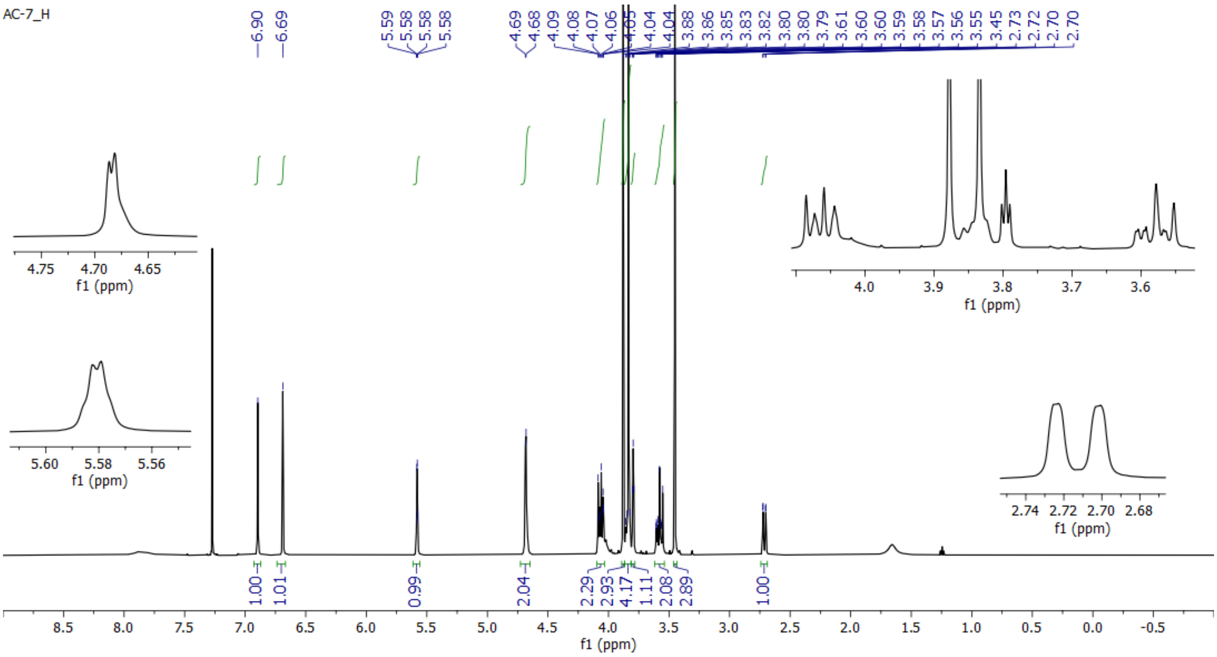

^{13}C NMR spectrum (125.7 MHz) of narcissidine (6) in $CDCl_3$

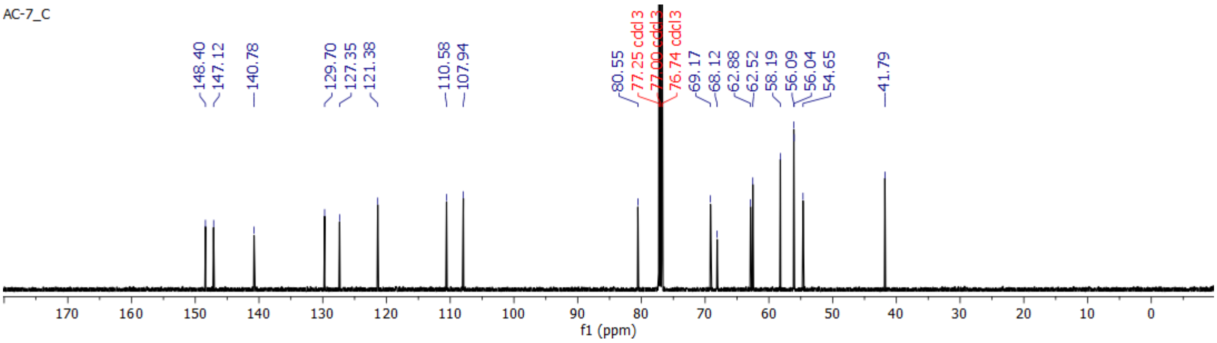

HSQC spectrum of narcissidine (6) in $CDCl_3$

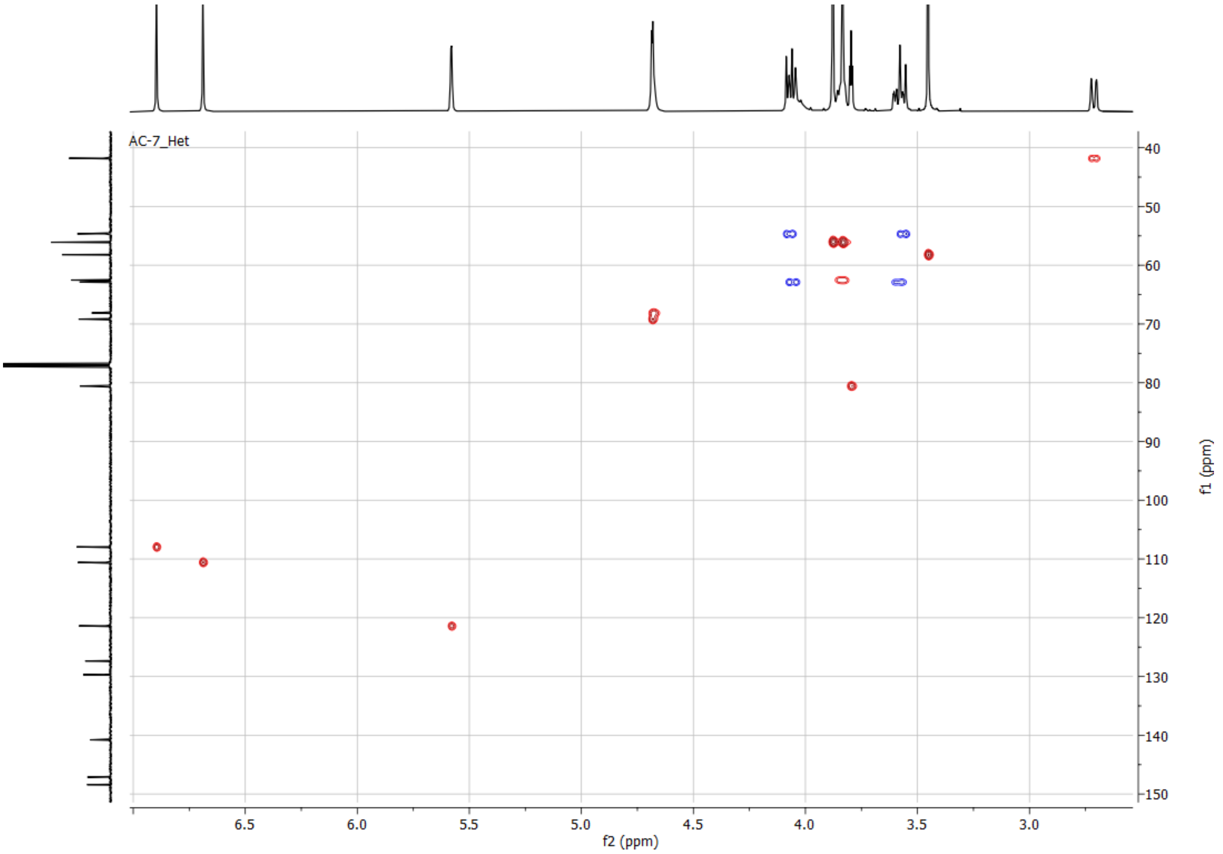

^{1}H NMR spectrum (500 MHz) of narcissidine (6) in CD_3OD

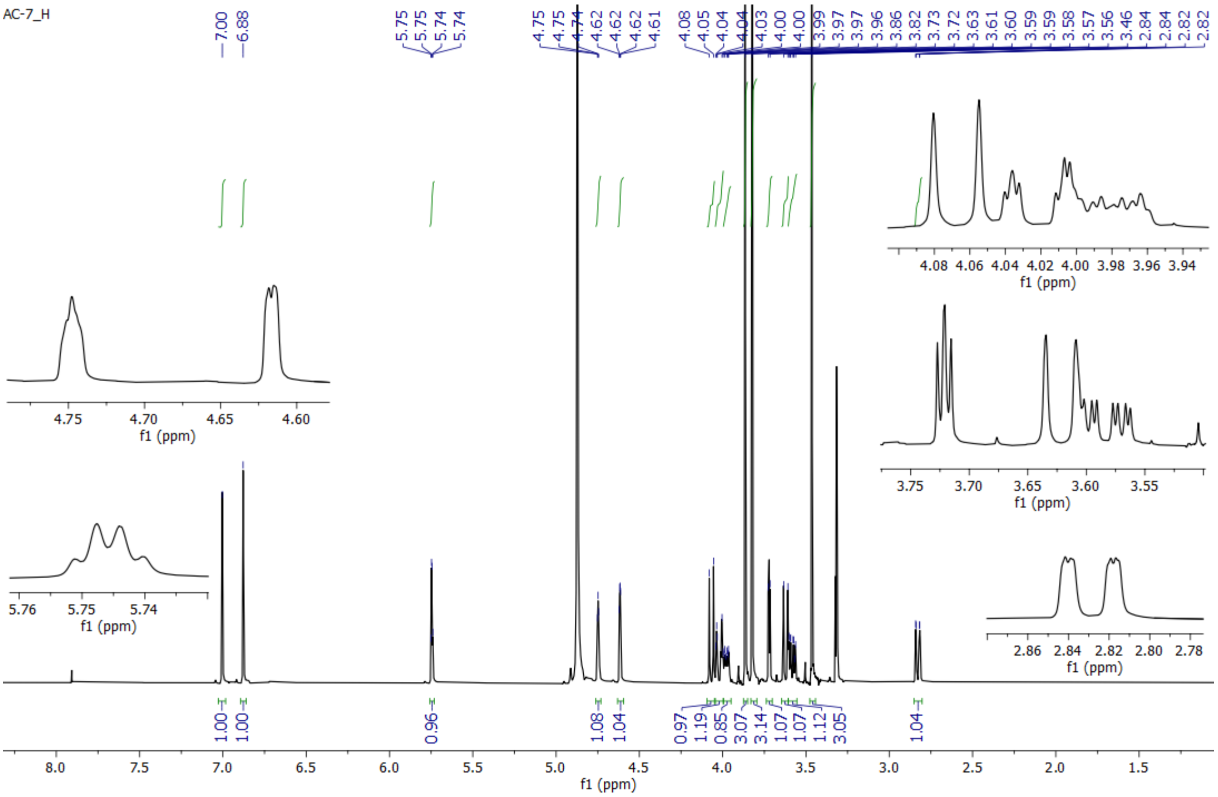

^{13}C NMR spectrum (125.7 MHz) of narcissidine (6) in CD_3OD

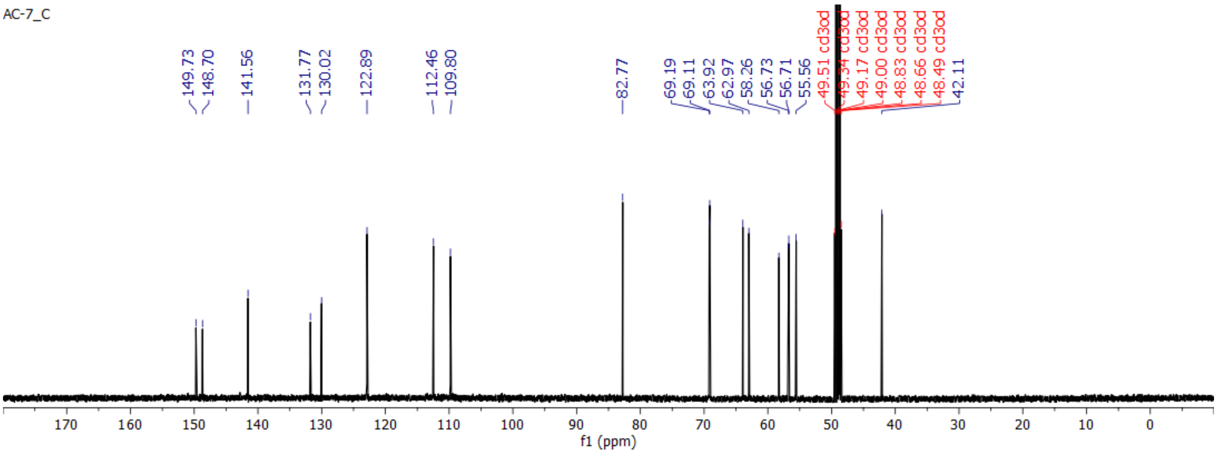

HSQC spectrum of narcissidine (6) in CD_3OD

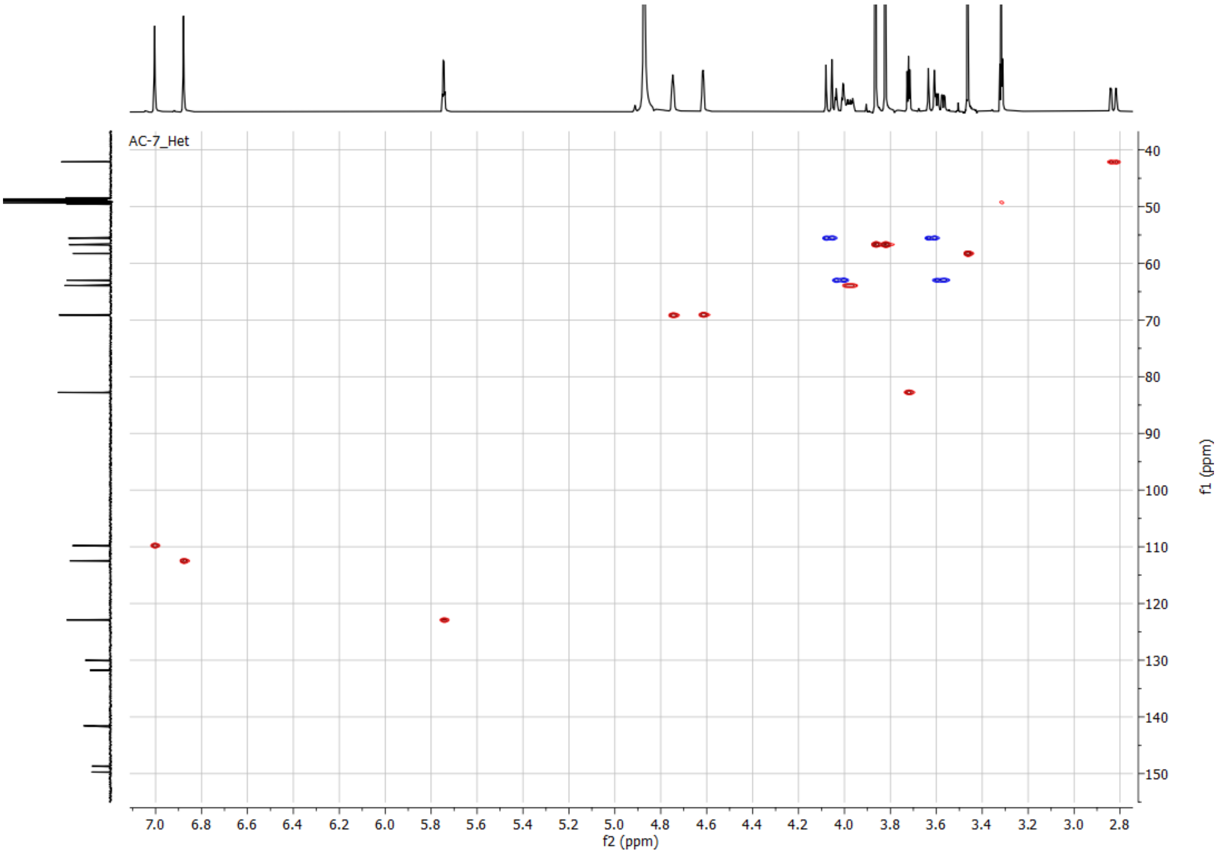

COSY spectrum of narcissidine (6) in CD_3OD

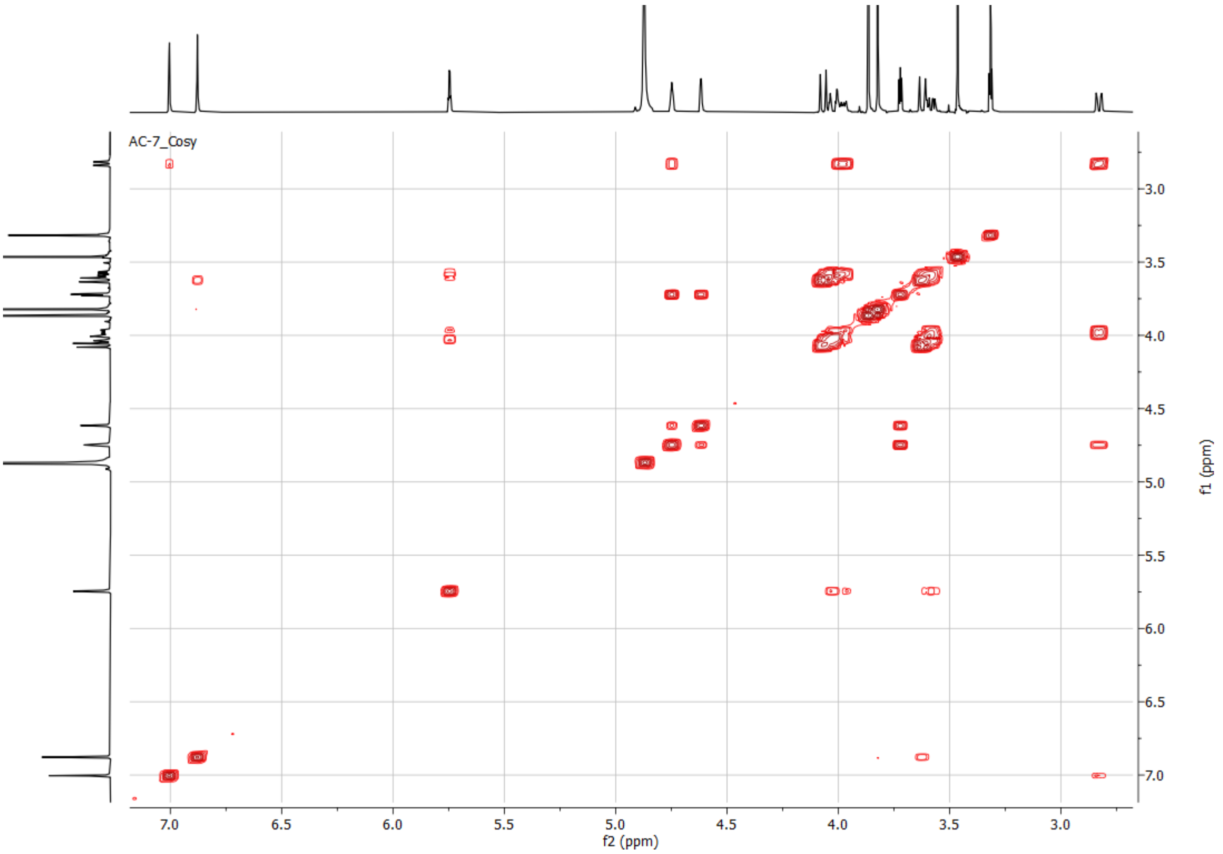

H2BC spectrum of narcissidine (6) in CD_3OD

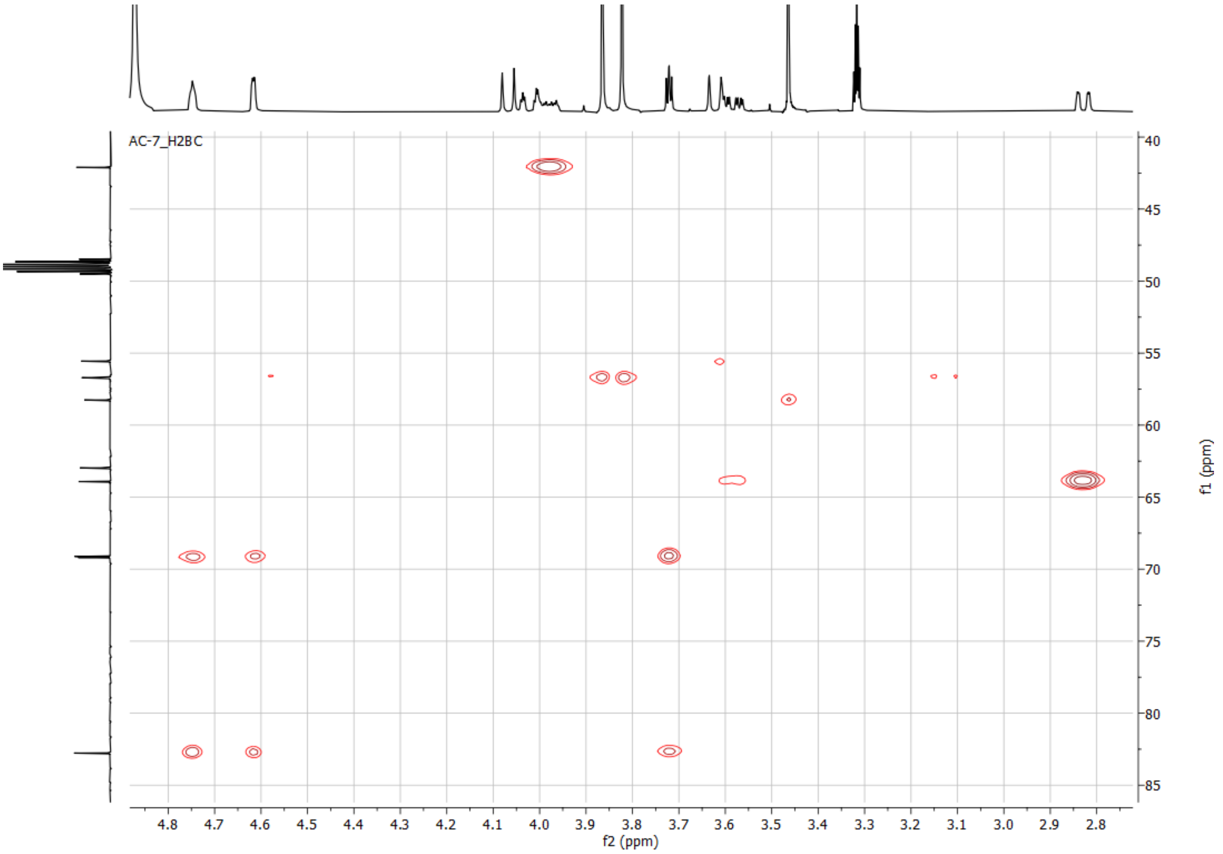

HMBC spectrum of narcissidine (6) in CD_3OD

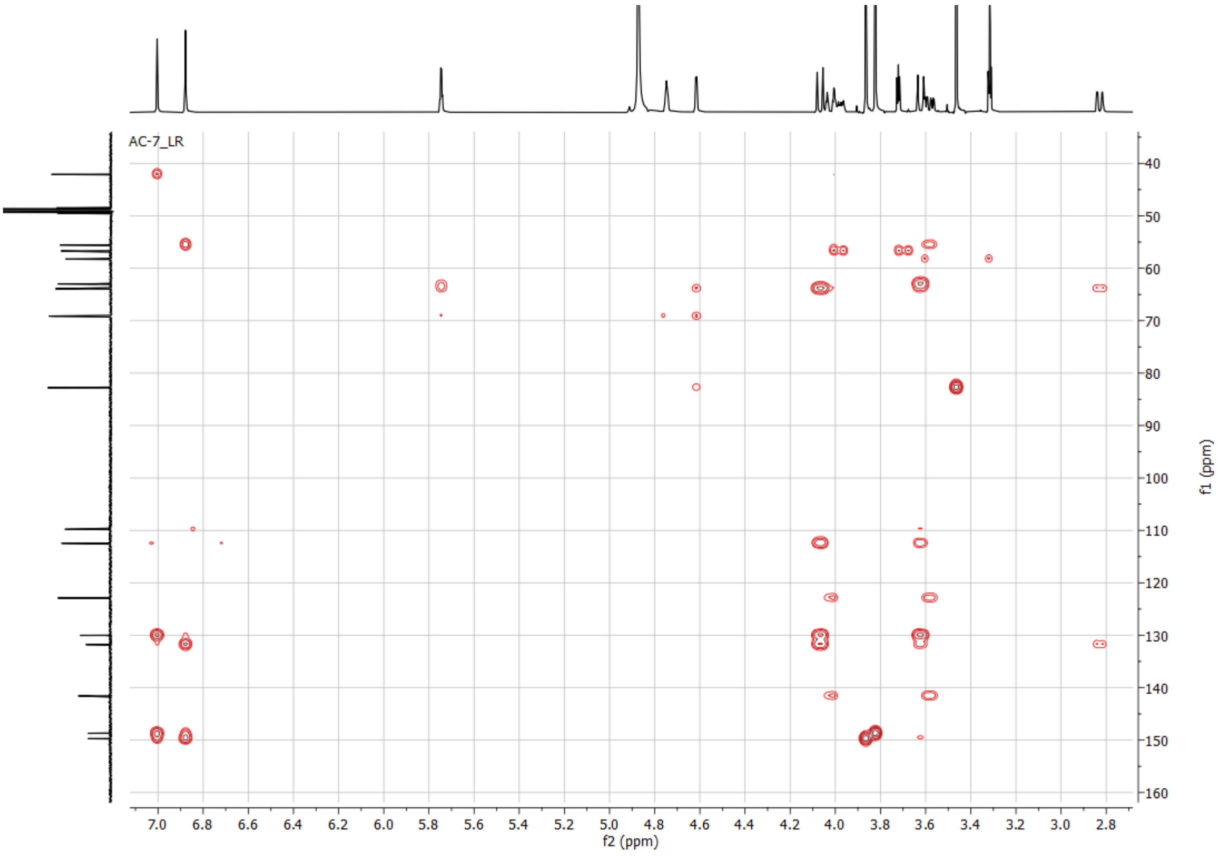

NOESY spectrum of narcissidine (6) in CD_3OD

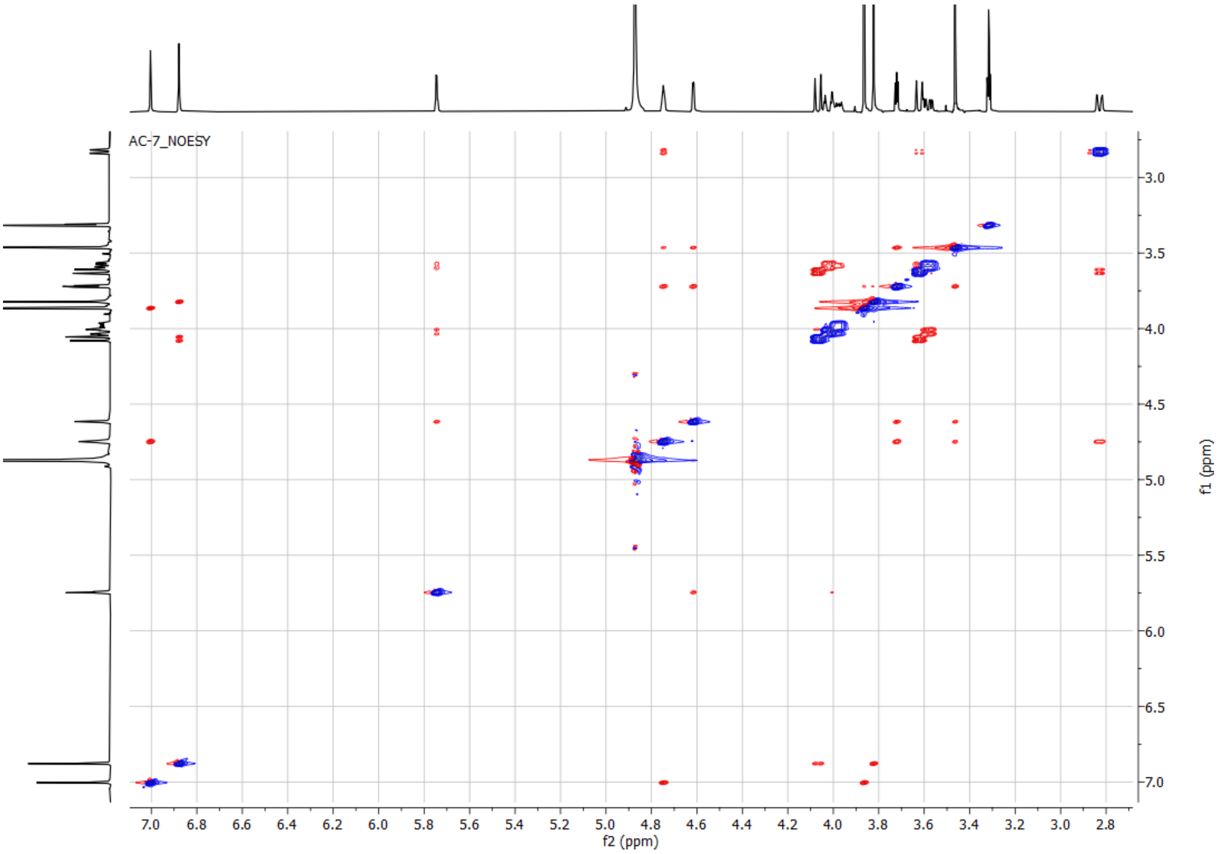

UV spectrum of narcissidine (6) in MeOH

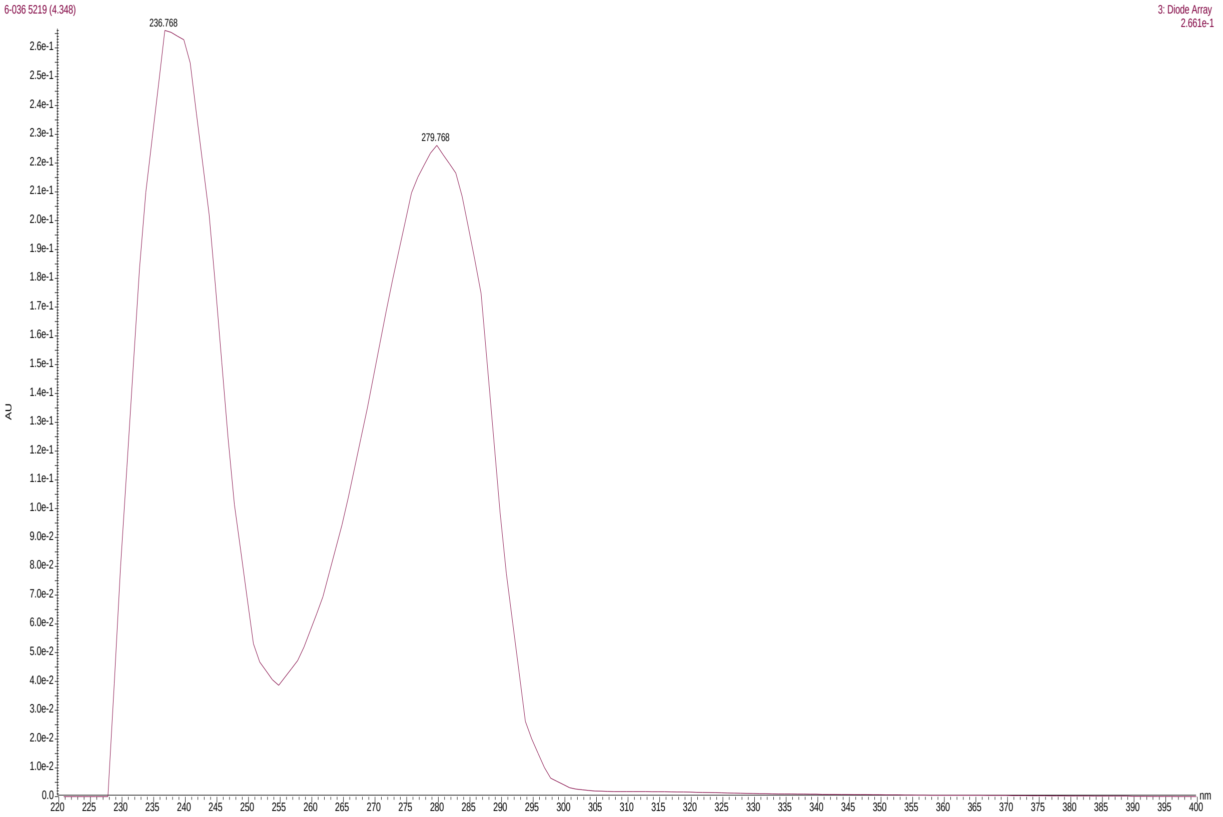

ECD spectrum of narcissidine (6) in MeOH

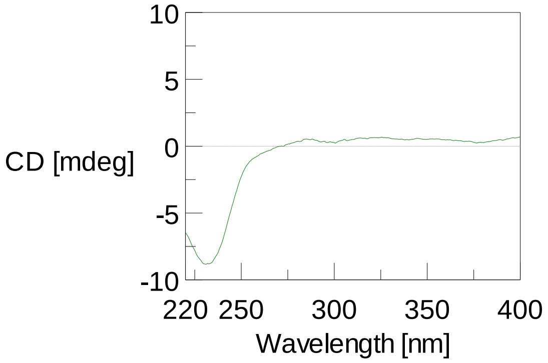

ECD (c 0.1 mg/ml, MeOH) [220–400 nm]

$\Delta\varepsilon_{231} = -8.92$ $[\theta]_{231} = -29418$

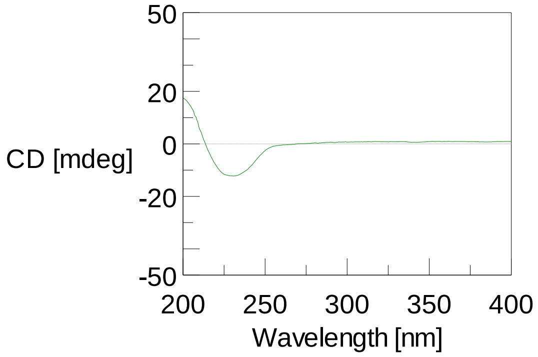

ECD (c 0.1 mg/ml, MeOH) [200–400 nm]

$\Delta\varepsilon_{200} = +17.80$ $[\theta]_{200} = +58702$

$\Delta\varepsilon_{231} = -12.35$ $[\theta]_{231} = -40730$

Table S3. Narcissidine: A comparison of NMR data measured in CD_3OD, $CDCl_3$, and those found in literature

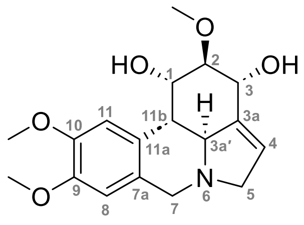

6

No.	**6**[a] δ_C	**6**[a] δ_H	**6**[b] δ_C	**6**[b] δ_H	**6**[b] [3] δ_H	**6**[b] [4] δ_C	**6**[b] [4] δ_H
1	69.2	4.79–4.73, m	68.1	4.70–4.65, m	4.66, m	67.9	4.70, m
2	82.8	3.72, dd (3.2, 2.6)	80.6	3.80, dd (3.2, 2.4)	4.18–3.36, m	80.0	3.81, t (3.0)
3	69.1	4.62, dd (2.6, 1.3)	69.2	4.70–4.65, m	4.66, m	68.9	4.69, d (3.0)
3a	141.6		140.8				
3a′	63.9	4.00–3.95, m	62.5	3.86–3.82, m [c]	4.18–3.36, m	62.0	3.87, m
4	122.9	5.75–5.73, m	121.4	5.59–5.57, m	5.56, m	120.9	5.61, m
5	63.0	4.05–4.00, m<br>3.58, ddd (14.2, 5.3, 2.1)	62.9	4.07–4.04, m [c]<br>3.58, ddd (14.5, 5.7, 2.0) [c]	4.18–3.36, m	62.1	4.07, dt (14.5, 2.0)<br>3.60, ddd (14.5, 5.5, 2.0)
7	55.6	4.07, d (12.9)<br>3.62, d (12.9)	54.6	4.07, d (13.0)<br>3.56, d (13.0)	4.09, d (13.0)<br>3.54, d (13.0)	54.0	4.10, d (13.0)<br>3.59, d (13.0)
7a	130.0		127.4				
8	112.5	6.88, s	110.6	6.69, s	6.68, s	110.2	6.70, s
9	148.7		147.1				
10	149.7		148.4				
11	109.8	7.00, s	107.9	6.90, s	6.88, s	107.3	6.90, s
11a	131.8		129.7				
11b	42.1	2.83, ddd (11.2, 1.8, 0.8)	41.8	2.71, dd (11.1, 1.4)	2.70, d (11.0)	41.2	2.74, dd (11.0, 2.0)
2-OCH_3	58.3	3.46, s	58.2	3.45, s	3.44, s	57.6	3.46, s
9-OCH_3	56.7	3.82, s	56.1	3.83, s [c]	3.82, s	55.6	3.84, s
10-OCH_3	56.7	3.86, s	56.0	3.88, s	3.86, s	55.6	3.89, s
$[\alpha]_D$		–56 ° (*c* 0.10, MeOH, 23 °C)			–23 ° (*c* 0.58, $CHCl_3$, 22 °C)		-

[a] CD_3OD, [b] $CDCl_3$, [c] overlapped

Data summary for compounds structurally related to 3, 4, 9, and 10

In the beginning, hippeastidine, which could be considered as the parent molecule of **10**, was first described in 1978 by Pacheco and co-workers (mp, IR, UV, low-resolution NMR, MS, [α]) when working with *Hippeastrum ananuca* Phil. (currently accepted as *Rhodophiala ananuca* (Phil.) Traub).[5] Later, its X-ray analysis was published.[6] Long after that, in 2013, a paper reported the isolation of hippeastidine from *Zephyranthes robusta* Baker (currently recognized as *Habranthus robustus* Herb.) with MS, NMR, and optical rotation data.[7] However, the optical rotation was given as $[\alpha]_D^{25}$ +330 (*c* 0.1, $CHCl_3$), which was quite different from the rotation that reported by Pacheco ($[\alpha]_D^{25}$ −6.9 (*c* 2.3, $CHCl_3$)). In the following year, de Andrade and co-workers described the isolation of aulicine, whose structure was elucidated by X-ray and NMR, among other techniques, resulting in the same structure as already described for hippeastidine in the above mentioned articles.[8] The justification for giving the molecule a new name was based on inconclusive literature data. When comparing everything known, the only article that deviates is the one from Kulhánková.[7] Not only the optical rotation, but also the NMR chemical shifts differ. In general, the data published in 1978 and 2014 seem like a match. Another paper reporting hippeastidine derivatives contributed to this confusion when compounds 6-hydroxyhippeastidine and 10-deoxy-6-hydroxyhippeastidine were published in 2014.[9] The relative configuration was established from NMR data. Interestingly, the authors described the impossibility of registering any Cotton effect in CD analysis and optical rotation was not measured. Hence, we found a solution for this. We noticed a significant visual difference when the samples were measured within the 200–400 nm and 220–400 nm ranges (see S23–S24). The most distinct maximum is usually between 200–210 nm, which is not a diagnostic maximum, and what is worse, it reduces the resolution of the other maxima, which are analytically valid. So, the spectrum may appear to have no Cotton effect. Valid maxima of Amaryllidaceae alkaloids of this type are found in the region of 240 nm and 280 nm. Thus, we

recommend analysis on the 220–400 nm scale to avoid false negative results. All the facts are summarized in **Figure S1**.

not isolated in our study, but named as hippeastidine

Pacheco et al., 1978
Watson et al., 1982
NMR, IR, UV, MS, m.p.
$[\alpha]_D$ −6.9 (*c* 2.3, $CHCl_3$)
X-ray
hippeastidine

de Andrade et al., 2014
NMR, MS, IR, UV
$[\alpha]_D$ −2.3 (*c* 0.4, $CHCl_3$)
CD $[\Theta]^{20}_{\lambda}$: $[\Theta]_{255}$ +1043, $[\Theta]_{279}$ −768
X-ray
aulicine

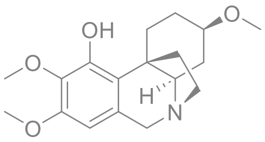

Kulhánková et al., 2013
NMR, MS, m.p.
$[\alpha]_D$ +330 (*c* 0.1, $CHCl_3$)
configuration not discussed
but given
hippeastidine

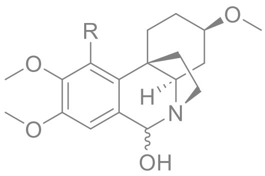

Shitara et al., 2014
NMR, MS, IR, UV, CD (no Cotton effect)
relative configuration proposed
10-deoxy-6α-hydroxyhippeastidine R=H
6α/β-hydroxyhippeastidine R=OH

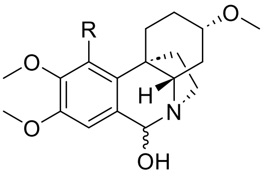

*mixture of 6-epimers
dr 75:25 (6*S* in excess)

Zhan et al., 2023
NMR, MS, CD - calculated and experimental, UV, IR

(**9**) **zephyranine E R=H**
$[\alpha]_D^{25}$ +23 (*c* 0.8, CH_3OH)
CD_{MeOH} $[\Theta]_{\lambda}$: $[\Theta]_{208}$ +15368, $[\Theta]_{233}$ −6530, $[\Theta]_{281}$ −3034
Our study:
$[\alpha]_D^{23}$ +67 (*c* 0.2, $CHCl_3$)
CD_{MeOH} $[\Theta]_{\lambda}$: $[\Theta]_{237}$ −12001, $[\Theta]_{283}$ −5202

(**10**) **zephyranine F R=OH**
$[\alpha]_D^{25}$ +21 (*c* 2.3, CH_3OH)
CD_{MeOH} $[\Theta]_{\lambda}$: $[\Theta]_{211}$ +19821, $[\Theta]_{233}$ −3660, $[\Theta]_{280}$ −1682
Our study:
$[\alpha]_D^{23}$ +61 (*c* 0.2, $CHCl_3$)
CD_{MeOH} $[\Theta]_{\lambda}$: $[\Theta]_{244}$ −2748, $[\Theta]_{281}$ −2815

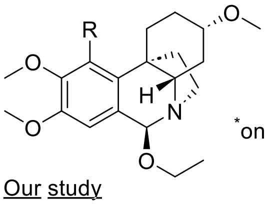

*only 6*S*-epimer

Our study

(**3**) **6-*O*-ethylzephyranine F R=OH**
$[\alpha]_D^{25}$ +44 (*c* 0.1, CH_3OH)

(**4**) **6-*O*-ethylzephyranine E R=H**
$[\alpha]_D^{25}$ +47 (*c* 0.2, CH_3OH)
CD_{MeOH} $[\Theta]_{\lambda}$: $[\Theta]_{241}$ −6398, $[\Theta]_{277}$ −5804

Figure S1. Data summary for compounds structurally related to **3**, **4**, **9**, and **10**. Stereochemistry of compounds adopted from cited articles.[5-10]

Table S4. Screening of compounds **1–8** for their cholinesterase inhibition

**compd**	**IC_{50} *h*AChE [μM]**	**IC_{50} *h*BChE [μM]**
**1**	> 100	> 100
**2**	> 100	> 100
**3**	> 100	> 100
**4**	> 100	> 100
**5**	> 100	> 100
**6**	> 100	> 100
**7**	> 100	> 100
**8**	> 100	> 100
**galanthamine***	1.72 ± 0.12	42.3 ± 1.3
**huperzine A***	0.033 ± 0.001	> 100
**eserine***	0.063 ± 0.005	0.13 ± 0.01

* a positive control

References

(1) Bohno, M.; Sugie, K.; Imase, H.; Yusof, Y. B.; Oishi, T.; Chida, N. Total Synthesis of Amaryllidaceae Alkaloids, (+)-Vittatine and (+)-Haemanthamine, Starting from D-glucose. *Tetrahedron* **2007**, *63*, 6977–6989. DOI: 10.1016/j.tet.2007.05.041.

(2) Pigni, N. B.; Ríos-Ruiz, S.; Martínez-Francés, V.; Nair, J. J.; Viladomat, F.; Codina, C.; Bastida, J. Alkaloids from *Narcissus serotinus*. *J. Nat. Prod.* **2012**, *75*, 1643–1647. DOI: 10.1021/np3003595.

(3) Kihara, M.; Ozaki, T.; Kobayashi, S.; Shingu, T. Alkaloidal Constituents of *Leucojum autumnale* L. (Amaryllidaceae). *Chem. Pharm. Bull.* **1995**, *43*, 318–320. DOI: 10.1248/cpb.43.318.

(4) Bradshaw, J.; Butina, D.; Dunn, A. J.; Green, R. H.; Hajek, M.; Jones, M. M.; Lindon, J. C.; Sidebottom, P. J. A Rapid and Facile Method for the Dereplication of Purified Natural Products. *J. Nat. Prod.* **2001**, *64*, 1541–1544. DOI: 10.1021/np010284g.

(5) Pacheco, P.; Silva, M.; Steglich, W.; Watson, W. H. Alkaloids of Chilean Amaryllidaceae I. Hippeastidine and epi-Homolycorine Two Novel Alkaloids. *Rev. Latinoam. Quim.* **1978**, *9*, 28–32.

(6) Watson, W. H.; Zabel, V.; Silva, M.; Pacheco, P. Hippeastidine, C17H23O4N. *Cryst. Struct. Commun.* **1982**, *11*, 157–162.

(7) Kulhánková, A.; Cahlíková, L.; Novák, Z.; Macáková, K.; Kuneš, J.; Opletal, L. Alkaloids from *Zephyranthes robusta* BAKER and their Acetylcholinesterase- and Butyrylcholinesterase-inhibitory Activity. *Chem. Biodivers.* **2013**, *10*, 1120–1127. DOI: 10.1002/cbdv.201200144.

(8) de Andrade, J. P.; Guo, Y.; Font-Bardia, M.; Calvet, T.; Dutilh, J.; Viladomat, F.; Codina, C.; Nair, J. J.; Zuanazzi, J. A. S.; Bastida, J. Crinine-type Alkaloids from *Hippeastrum aulicum* and *H. calyptratum*. *Phytochemistry* **2014**, *103*, 188–195. DOI: 10.1016/j.phytochem.2014.03.007.

(9) Shitara, N.; Hirasawa, Y.; Hasumi, S.; Sasaki, T.; Matsumoto, M.; Wong, C. P.; Kaneda, T.; Asakawa, Y.; Morita, H. Four New Amaryllidaceae Alkaloids from *Zephyranthes candida*. *J. Nat. Med.* **2014**, *68*, 610–614. DOI: 10.1007/s11418-014-0819-y.

(10) Zhan, G.; Gao, B.; Zhou, J.; Liu, T.; Zheng, G.; Jin, Z.; Yao, G. Structurally Diverse Alkaloids with Nine Frameworks from *Zephyranthes candida* and their Acetylcholinesterase Inhibitory and Anti-inflammatory Activities. *Phytochemistry* **2023**, *207*, 113564. DOI: 10.1016/j.phytochem.2022.113564.
